# Analysis of Plasma Cell-Free DNA by Ultradeep Sequencing in Patients With Stages I to III Colorectal Cancer

**DOI:** 10.1001/jamaoncol.2019.0528

**Published:** 2019-05-09

**Authors:** Thomas Reinert, Tenna Vesterman Henriksen, Emil Christensen, Shruti Sharma, Raheleh Salari, Himanshu Sethi, Michael Knudsen, Iver Nordentoft, Hsin-Ta Wu, Antony S. Tin, Mads Heilskov Rasmussen, Søren Vang, Svetlana Shchegrova, Amanda Frydendahl Boll Johansen, Ramya Srinivasan, Zoe Assaf, Mustafa Balcioglu, Alexander Olson, Scott Dashner, Dina Hafez, Samantha Navarro, Shruti Goel, Matthew Rabinowitz, Paul Billings, Styrmir Sigurjonsson, Lars Dyrskjøt, Ryan Swenerton, Alexey Aleshin, Søren Laurberg, Anders Husted Madsen, Anne-Sofie Kannerup, Katrine Stribolt, Søren Palmelund Krag, Lene H. Iversen, Kåre Gotschalck Sunesen, Cheng-Ho Jimmy Lin, Bernhard G. Zimmermann, Claus Lindbjerg Andersen

**Affiliations:** 1 Department of Molecular Medicine, Aarhus University Hospital, Aarhus, Denmark; 2 Natera Inc, San Carlos, California; 3 Department of Surgery, Aarhus University Hospital, Aarhus, Denmark; 4 Department of Surgery, Regional Hospital Herning, Herning, Denmark; 5 Department of Surgery, Regional Hospital Randers, Randers, Denmark; 6 Department of Pathology, Regional Hospital Randers, Randers, Denmark; 7 Department of Pathology, Aarhus University Hospital, Aarhus, Denmark

## Abstract

**Importance:**

Novel sensitive methods for detection and monitoring of residual disease can improve postoperative risk stratification with implications for patient selection for adjuvant chemotherapy (ACT), ACT duration, intensity of radiologic surveillance, and, ultimately, outcome for patients with colorectal cancer (CRC).

**Objective:**

To investigate the association of circulating tumor DNA (ctDNA) with recurrence using longitudinal data from ultradeep sequencing of plasma cell-free DNA in patients with CRC before and after surgery, during and after ACT, and during surveillance.

**Design, Setting, and Participants:**

In this prospective, multicenter cohort study, ctDNA was quantified in the preoperative and postoperative settings of stages I to III CRC by personalized multiplex, polymerase chain reaction–based, next-generation sequencing. The study enrolled 130 patients at the surgical departments of Aarhus University Hospital, Randers Hospital, and Herning Hospital in Denmark from May 1, 2014, to January 31, 2017. Plasma samples (n = 829) were collected before surgery, postoperatively at day 30, and every third month for up to 3 years.

**Main Outcomes and Measures:**

Outcomes were ctDNA measurement, clinical recurrence, and recurrence-free survival.

**Results:**

A total of 130 patients with stages I to III CRC (mean [SD] age, 67.9 [10.1] years; 74 [56.9%] male) were enrolled in the study; 5 patients discontinued participation, leaving 125 patients for analysis. Preoperatively, ctDNA was detectable in 108 of 122 patients (88.5%). After definitive treatment, longitudinal ctDNA analysis identified 14 of 16 relapses (87.5%). At postoperative day 30, ctDNA-positive patients were 7 times more likely to relapse than ctDNA-negative patients (hazard ratio [HR], 7.2; 95% CI, 2.7-19.0; *P* < .001). Similarly, shortly after ACT ctDNA-positive patients were 17 times (HR, 17.5; 95% CI, 5.4-56.5; *P* < .001) more likely to relapse. All 7 patients who were ctDNA positive after ACT experienced relapse. Monitoring during and after ACT indicated that 3 of the 10 ctDNA-positive patients (30.0%) were cleared by ACT. During surveillance after definitive therapy, ctDNA-positive patients were more than 40 times more likely to experience disease recurrence than ctDNA-negative patients (HR, 43.5; 95% CI, 9.8-193.5 *P* < .001). In all multivariate analyses, ctDNA status was independently associated with relapse after adjusting for known clinicopathologic risk factors. Serial ctDNA analyses revealed disease recurrence up to 16.5 months ahead of standard-of-care radiologic imaging (mean, 8.7 months; range, 0.8-16.5 months). Actionable mutations were identified in 81.8% of the ctDNA-positive relapse samples.

**Conclusions and Relevance:**

Circulating tumor DNA analysis can potentially change the postoperative management of CRC by enabling risk stratification, ACT monitoring, and early relapse detection.

## Key Points

**Question:**

Does analysis of longitudinal data from circulating tumor DNA enable residual disease detection and risk-stratified postoperative management of stages I-III colorectal cancer?

**Findings:**

In this cohort study of 125 patients and 795 plasma samples from Denmark, circulating tumor DNA was associated with relapse as were current identified risk factors, both before and after adjuvant therapy and during long-term surveillance. Furthermore, longitudinal circulating tumor DNA data analysis enabled early relapse detection and assessment of adjuvant chemotherapy effectiveness.

**Meaning:**

Analysis of longitudinal data from circulating tumor DNA may have implications for postoperative management of colorectal cancer that includes guiding adjuvant chemotherapy patient selection, guiding adjuvant chemotherapy duration optimization, and enabling earlier detection of clinical relapse.

## Teaser

This cohort study investigates the use of longitudinal data from ultradeep sequencing of cell-free DNA in Danish patients with colorectal cancer.

## Introduction

With 1.3 million newly diagnosed cases each year, colorectal cancer (CRC) is the third most common cancer worldwide and the second leading cause of cancer-related deaths.^1^ Despite improved surgery, implementation of screening, and advances in treatment regimens, the 5-year mortality rate for patients with CRC remains high at approximately 40%, thereby representing a significant global health burden.^2,3^

The current standard of care for patients with CRC includes surgical resection of the tumor followed by adjuvant chemotherapy (ACT) in selected patients.^4,5^ Most patients with stage II CRC are not treated with ACT; however, approximately 10% to 15% have residual disease after surgery.^3^ Identification of this patient population and treatment with ACT could potentially reduce their risk of recurrence. Conversely, most patients with stage III CRC receive ACT^6^ despite more than 50% being cured by surgery.^7,8^ Furthermore, approximately 30% of the ACT-treated patients with stage III CRC experience recurrence, making them candidates for additional therapy.^3,9^ Thus, improved tools to identify the patient population who would benefit from ACT are greatly needed.

Early diagnosis of recurrent disease is another significant unmet clinical need in CRC. After completion of definitive treatment, surveillance is recommended to detect recurrence sufficiently early for potentially curative surgery.^4,5,10^ Despite surveillance, many recurrence events are detected late, and only 10% to 20% of metachronous metastases are treated with curative intent.^11,12^ Therefore, there is a need for better biomarkers that can detect patients at high risk of recurrence, thereby enabling appropriate follow-up and therapeutic strategies for early recurrence detection and curative treatment.^13^

Circulating tumor DNA (ctDNA) has emerged as a promising noninvasive biomarker for longitudinal assessment of a tumor throughout disease management. In CRC, there are multiple indications for which ctDNA can assist with clinical decision making.^14,15,16,17,18^

We report results from a prospective and observational biomarker study in patients with stages I to III CRC with an aim to demonstrate that postoperative detection of ctDNA is associated with residual disease and high relapse risk and that longitudinal analysis enables residual disease monitoring throughout the disease course. Using a personalized, tumor-specific, multiplex polymerase chain reaction (PCR)–based next-generation sequencing (NGS) method for ctDNA detection, we demonstrate that ctDNA is detected preoperatively in patients with CRC and that postoperative ctDNA analysis enables monitoring of ACT treatment effectiveness, detection of residual disease before and after ACT treatment, early detection of recurrence, and detection of actionable mutations.

## Methods

This prospective, multicenter study recruited patients with stages I to III CRC from May 1, 2014, to January 31, 2017, at the surgical departments of Aarhus University Hospital, Randers Hospital, and Herning Hospital in Denmark. Tumor tissue was collected at surgery. Blood samples (n = 829) were collected before surgery (up to 14 days preoperatively) and at postoperative day 30 (ie, sample drawn up to 14 days before or after day 30) and then at every third month until death, patient withdrawal from the study, or month 36, whichever came first. Data on postsurgery clinical intervention and other clinicopathologic information were collected for all patients (eTable 1 in the Supplement). All patients received treatment and follow-up in compliance with the national guidelines defined by the Danish Colorectal Cancer Group. The ctDNA analyses were performed retrospectively by Natera Inc, with analysts blinded to patient outcome and sample order. Neither treating clinicians nor patients were informed about the ctDNA results. Methodologic details are available in eMethods 1 to 5 in the Supplement. The study was approved by the Committees on Biomedical Research Ethics in the Central Region of Denmark and was performed in accordance with the Declaration of Helsinki.^19^ All participants provided written informed consent.

### Multiplex PCR-Based NGS of Plasma Cell-Free DNA

On the basis of tumor whole-exome sequencing, 16 high-ranked patient-specific somatic single-nucleotide variants and short indels were selected for each patient. Multiplex PCR primer pairs for the chosen set of variants were generated as previously described.^20^ Cell-free DNA was extracted from a median of 8.5 mL (interquartile range, 7.5-9-5 mL) of plasma. Universal libraries were created by end repair, A-tailing, and ligation with custom adapters, as previously described.^20^ Next, libraries were amplified by multiplex PCR, barcoded, pooled, and sequenced on an NGS sequencing platform (HiSeq 2500 system, Illumina Inc). Plasma samples with at least 2 variants detected were defined as ctDNA positive. For details, see eMethods 2 through 9, eResults 1, and eTable 2 in the Supplement.

### Statistical Analysis

The primary outcome measure was recurrence-free survival (RFS) assessed by standard radiologic criteria. Recurrence-free survival was measured from the date of surgery to the verified first radiologic recurrence (local or distant) or death as a result of CRC and was censored at last follow-up or non-CRC–related death. Patients with no follow-up were excluded from the study. Survival analysis was performed using the Kaplan-Meier method. Cox proportional hazards regression analysis was used to assess the association of ctDNA and carcinoembryonic antigen (CEA) with RFS. Multivariate analysis was performed with clinical variables that were statistically significant in univariate analysis. The proportional hazards assumption was tested by a global test of the Schoenfeld residuals. All *P* values were based on 2-sided testing, and differences were considered significant at *P* ≤ .05. Statistical analysis was performed using Stata IC/12.1 software (StataCorp) and R statistical software, version 2.4 for Windows (R Foundation for Statistical Computing).

## Results

**Figure 1.**
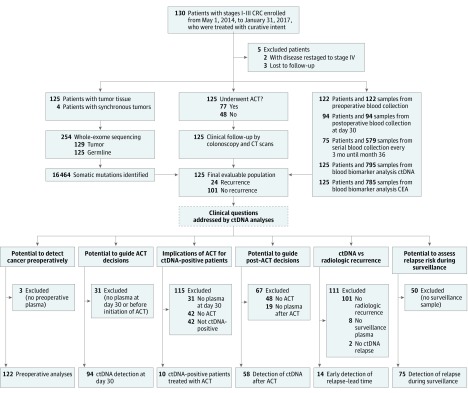
Patient Enrollment, Sample Collection, and Definition of the Patient Subgroups Used to Address the Defined Clinical Questions ACT indicates adjuvant chemotherapy; CEA, carcinoembryonic antigen; CRC, colorectal cancer; CT, computed tomography; and ctDNA, circulating tumor DNA.

A total of 130 patients with International Union Against Cancer stages I to III CRC (mean [SD] age, 67.9 [10.1] years; 74 [56.9%] male) were enrolled in the study. Patient enrollment and study overview are presented in Figure 1. Five patients were subsequently excluded because they were lost to follow-up (n = 3) or reclassified as having stage IV disease. Patient characteristics and demographics are detailed in eTable 3 in the Supplement. Whole-exome sequencing of tumor and matched germline DNA was used to identify somatic mutations (eFigure 1 and eTable 4 in the Supplement). Tumor-specific multiplex PCR assay panels that targeted 16 mutations were designed for each patient. Ultradeep multiplex PCR–based NGS (median target coverage, >105 000 reads) (eFigure 2 in the Supplement) was used to analyze and quantify ctDNA in 795 plasma samples from 125 patients with a median follow-up of 12.5 months (range, 1.4-38.5 months) (Figure 1). Detailed information regarding ctDNA results and dynamics for all 125 patients are listed in eTable 5 and shown in eFigure 3 in the Supplement. During this period, 24 patients (19.2%) experienced radiologic recurrence (eTable 3 in the Supplement).

### Preoperative Detection of ctDNA 

In the 122 baseline preoperative plasma samples, ctDNA was detected in 108 of 122 samples (88.5%), with a sensitivity of 40% for stage I disease, 92% for stage II disease, and 90% for stage III disease (eFigure 4 in the Supplement). By contrast, CEA was detected in only 53 of 122 samples (43.3%) (eFigure 4 in the Supplement).

### Association of ctDNA Status at Postoperative Day 30 With Risk of Recurrence

**Figure 2.**
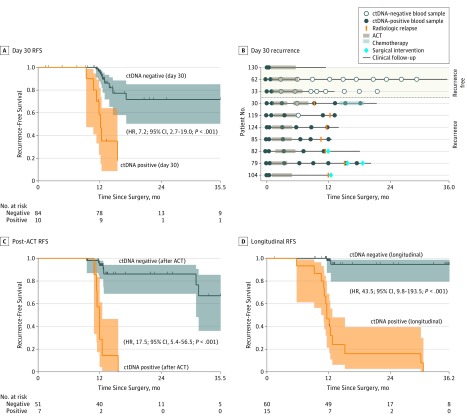
Preoperative and Postoperative Circulating Tumor DNA (ctDNA) Monitoring in Patients With Colorectal Cancer (CRC) A, Kaplan-Meier estimates of recurrence-free survival (RFS) for 94 patients with stages I to III CRC stratified by postoperative day 30 ctDNA status. The 3 censored ctDNA-positive patients were all treated with adjuvant chemotherapy (ACT) and were likely cured by this treatment (see patients 33, 62, and 130 in B). B, Recurrence rate and longitudinal ctDNA status in ctDNA-positive patients receiving ACT. C, Kaplan-Meier estimates of RFS for 58 ACT-treated patients, stratified by ctDNA status at first post-ACT visit. D, Kaplan-Meier estimates of RFS for 75 patients with longitudinal samples, stratified by longitudinal post–definitive-treatment ctDNA status. A patient was classified as testing positive if 1 or more plasma samples after definitive treatment was ctDNA positive. The Kaplan-Meier plots were halted when the proportion of patients in follow-up was less than 10%. Shaded areas in the Kaplan-Meier plots indicate 95% CIs. HR indicates hazard ratio.

To assess whether ctDNA status is associated with residual disease and future recurrence, ctDNA analysis was performed on postoperative plasma samples. Plasma collected at day 30, before the start of ACT, was available for 94 patients. Of these patients, 84 (89.4%) were ctDNA negative, and 10 (10.6%) were positive for ctDNA (eFigure 5 in the Supplement). These ctDNA-positive patients had a significantly higher recurrence rate (70.0%, [7 of 10 patients]; 95% CI, 34.2%-93.1%) compared with those who were ctDNA negative after surgery (11.9% [10 of 84]; 95% CI, 6.3%-20.1%). The presence of ctDNA was associated with a markedly reduced RFS compared with ctDNA-negative patients (hazard ratio [HR], 7.2; 95% CI, 2.7-19.0; *P* < .001) (Figure 2A). In a multivariate logistic regression model, including ctDNA status and known risk factors, such as stage and lymphovascular invasion, ctDNA status was the only significant prognostic factor associated with RFS (eTable 6 in the Supplement). A subset of the patients were treated with ACT (n = 52), but even for this subset, ctDNA positivity was associated with a high risk of recurrence (HR, 7.1; 95% CI 2.2-22.0; *P* < .001) (eFigure 6 in the Supplement). The relapse rate for ctDNA-negative patients was 12%, independent of whether they were treated with ACT (5 of 42 patients) or not (5 of 42).

### Association of ACT With ctDNA Clearance

Although randomized studies^21,22,23,24^ have found that ACT can reduce the overall recurrence rate of stage III CRC, it is currently unknown whether ACT is specifically associated with the prevention of recurrences among the high-risk ctDNA-positive subfraction. The 10 patients who were positive for ctDNA at day 30 were all subsequently treated with ACT (Figure 2B). Of these, 7 (70.0%) relapsed, whereas 3 (30.0%) were still disease free at the end of follow-up, indicating an association between ACT and residual disease clearance in a subfraction of ctDNA-positive patients. Consistent with the association between ACT and residual disease elimination, disease-free patients with available longitudinal plasma samples had complete clearance of ctDNA during therapy and remained ctDNA negative for the duration of the study. Conversely, the 6 patients with disease recurrence who had available longitudinal plasma samples remained ctDNA positive during ACT or regained ctDNA-positive status shortly after completion of ACT.

###  Use of Longitudinal ctDNA Monitoring to Assess ACT Treatment Effectiveness

Longitudinally collected blood samples were available for 8 of 10 patients who were ctDNA positive before the start of ACT, which afforded us a unique opportunity to observe the changes in ctDNA levels during treatment. The ctDNA was cleared in 4 of 8 patients (50.0%) (Figure 2B), whereas in the remaining 4 patients ctDNA status remained positive throughout treatment. Strikingly, all 4 patients who did not clear ctDNA experienced disease recurrence, indicating that residual ctDNA is associated with ACT failing to eliminate the residual disease. Of the 4 patients who cleared ctDNA during treatment, 2 remained ctDNA negative in all post-ACT samples and consistently have not experienced disease recurrence, whereas the other 2 patients regained ctDNA positivity shortly after treatment and relapsed (Figure 2B).

###  Association of ctDNA Status After ACT With Risk of Recurrence

Because 100% of the patients who did not clear ctDNA during ACT subsequently experienced disease relapse, we hypothesized that ctDNA analysis of the first blood sample drawn after ACT can be used to identify a subgroup of patients with continued residual disease who could benefit from further treatment. Of the 58 patients with post-ACT blood samples, 7 of the 7 ctDNA-positive patients (100%; 95% CI, 59%-100%) relapsed. In comparison, of the 51 ctDNA-negative patients, 7 (13.7%) relapsed (95% CI, 6.3%-26.1%; Fisher exact test, *P* < .001). Univariate analysis showed that ctDNA status was significantly associated with recurrence (HR, 17.5; 95% CI, 5.4-56.5; *P* < .001) (Figure 2C). In a multivariate logistic regression model, including ctDNA status and risk factors, such as stage, lymphovascular invasion, and microradical resection status (eTable 7 in the Supplement), ctDNA status was the only significant factor.

### Association of Longitudinal ctDNA Analysis With Patient Outcome 

Serial ctDNA analysis during surveillance after definitive treatment of the 75 patients with longitudinal collected plasma samples identified relapse with 88% sensitivity and 98% specificity. Strikingly, 14 of the 15 ctDNA-positive patients (93.3%) experienced disease recurrence compared with 2 of the 60 ctDNA-negative patients (3.3%) (Fisher exact test, *P* < .001). The ctDNA-positive patients had a markedly reduced RFS (HR, 43.5; 95% CI, 9.8-193.5 *P* < .001) (Figure 2D).

The disease course and longitudinal ctDNA results are shown in eFigure 7 in the Supplement for all 75 patients. The serial ctDNA analysis missed 2 metastatic relapses (patients 20 and 24) (eFigure 7 in the Supplement). Whole-exome sequencing of the 2 missed metastases nevertheless confirmed the presence of the mutations used for plasma screening (eTable 8 and eResults 2 in the Supplement). Longitudinal CEA analysis of this same population identified relapse with a sensitivity of 69% and specificity of 64% (eFigure 8 in the Supplement). In multivariable analysis, ctDNA was the only factor significantly associated with RFS (HR, 39.9; 95% CI, 7.5-211.0; *P* < .001) (eTable 9 in the Supplement).

**Figure 3.**
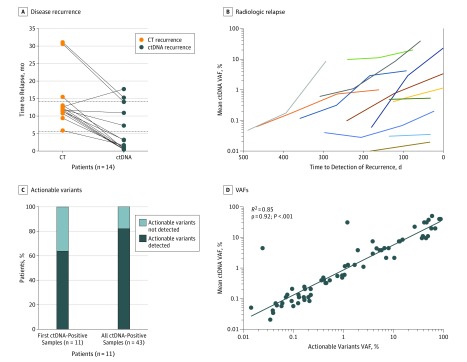
Association of Circulating Tumor DNA (ctDNA) Analysis With Early Detection of Relapse and Detection of Clinical Actionable Mutations A, Comparison of time to relapse by ctDNA and standard-of-care computed tomography (CT). The mean time from surgery to relapse detection was 5.5 months (range, 0.4-17.7 months) for ctDNA and 14.2 months (range, 5.9-31.1 months for CT). Dashed lines indicate mean time in months of recurrence based on CT and ctDNA. B, For all patients with relapsing disease, the ctDNA levels in plasma increased over time from ctDNA detection to radiologic response. Early time points before and during adjuvant chemotherapy were omitted. Each colored curve represents data from a different patient. C, Fraction of recurrence in ctDNA-positive patients with actionable mutations detected in plasma. D, The actionable variants occurred with variant allele frequencies (VAFs) similar to the nonactionable variants. Association between the mean ctDNA VAF and the VAF of the actionable mutations is shown.

The mean lead time from ctDNA detection in plasma to relapse detection by standard-of-care computed tomography was 8.7 months (range, 0.8-16.5 months) (Wilcoxon signed rank test; *P* < .001) (Figure 3A); by contrast, CEA revealed no lead time (eFigure 9 in the Supplement). From ctDNA detection after curative intended treatment and until radiologic relapse detection, plasma samples remained ctDNA positive. We observed an increase in the ctDNA variant allele frequency in all patients, up to 300-fold (median, 5; 95% CI, 1.4-174.0), indicating that the tumor burden often increased notably while the patients awaited radiologic detection of the relapse (Figure 3B).

### ctDNA Analysis of Clinically Actionable Mutations

Having shown that longitudinal ctDNA analysis enables detection of micrometastatic disease months before radiologic relapse, we next investigated whether the ctDNA analyses in parallel could inform about the presence of potentially actionable mutations at this early recurrence time point. We identified 11 patients with disease recurrence, available longitudinal samples, and clinically actionable mutations identified by primary tumor whole-exome sequencing (eTable 10 in the Supplement). As a proof-of-concept analysis, additional multiplex PCR panels targeting the actionable mutations were designed and applied to the longitudinal samples. For 7 of the 11 patients (63.6%), an actionable mutation was detected already in the first ctDNA-positive sample; when all ctDNA-positive samples were analyzed, 9 of the 11 patients (81.8%) had actionable mutations (Figure 3C). We observed a significant correlation (Spearman ρ = 0.92; *R*^2^ = 0.85; *P* < .001) between the mean ctDNA and actionable mutation allele frequencies (Figure 3D).

## Discussion

The preoperative and postoperative results presented in this study are in accordance with and expand on results presented previously.^14,15,25^ We found that longitudinal ctDNA analysis in patients with stages I to III CRC can effectively detect and monitor changes in tumor burden throughout the clinical disease course. Specifically, we show that ctDNA serves as a robust biomarker for (1) postoperative and post-ACT risk stratification, (2) monitoring ACT effectiveness, (3) detection of clinical actionable mutations, and (4) early detection of recurrence. These observations have important and potential paradigm-changing implications for the future of postoperative management of CRC (eFigure 10 in the Supplement) and lay the foundation for future intervention trials to investigate the clinical benefits of ctDNA-guided management.

In the preoperative context, we found detection rates that are similar to previous studies,^25,26,27^ confirming our ctDNA detection technique. The reliability and reproducibility of the technique were further supported by comparing the ctDNA status of the serial plasma samples and the clinical disease course. Blood samples drawn after curative treatment were expected to test negative for ctDNA. Consistent with this theory, 455 of the 456 postoperative serial blood samples (99.8%) from patients without disease relapse were ctDNA negative (eFigure 7 in the Supplement). By contrast, the serial analysis of the 16 patients with disease relapse detected ctDNA in 14 patients (87.5%) (eFigure 7 in the Supplement). Furthermore, the serial samples were persistently positive. Only in cases of clinical intervention did the ctDNA status change from positive to negative (eg, patients 75 and 119) (eFigure 7 in the Supplement).

Currently, decision making for ACT treatment is based on risk stratification by stage and clinical risk factors. We found that in multivariate analysis ctDNA status (among stage, CEA, and other high-risk factors) was the only significant factor associated with recurrence. This suggests that ctDNA analysis may be a better tool for identifying high risk patients. Hence, in the future, it may be possible to use ctDNA-analyses to identify a ctDNA positive subgroup of patients with stages I and II disease who could potentially benefit from ACT (eFigure 10 in the Supplement, trial 1). We and others are currently conducting trials to assess the clinical benefit of ctDNA-based patient selection in this setting (eg, IMPROVE-IT [Intervention Trial Implementing Noninvasive Circulating Tumor DNA Analysis to Optimize the Operative and Postoperative Treatment for Patients With Colorectal Cancer]^28^ and Circulating Tumour DNA [ctDNA] Analysis Informing Adjuvant Chemotherapy in Stage II Colon Cancer^29^). We also found that ctDNA-negative patients have similar low risk of relapsing, independent of whether or not ACT was administered. Hence, in the future, it may be possible to withhold ACT from ctDNA-negative but clinically high-risk patients (those with stage III disease), with a minimal alteration in their relapse risk (eFigure 10 in the Supplement). This patient group could be offered active ctDNA-based surveillance instead of ACT, thus sparing the many patients who are cured by surgery alone from the toxic effects of chemotherapy. In addition, in the post-ACT setting, where there are no current risk markers, we demonstrate that ctDNA analysis identifies patients who still have residual disease. This population may benefit from intensified therapeutic treatment.

We also found that longitudinal ctDNA monitoring before, during, and after ACT provides a patient-level measurement of ACT effectiveness. The 30% of patients who cleared ctDNA and remained negative in all subsequent samples stayed disease free throughout the study. Thus, our study provides first-line evidence that ACT can reduce the risk of recurrence in ctDNA-positive patients. This risk reduction is similar to that estimated when ACT is given to all patients with stage III colon cancers.^21,22,23,24^ We found that all patients who did not clear ctDNA had disease relapse within a year of completion of ACT. In addition, all patients with only transient clearance of ctDNA also experienced relapse. These findings need to be further validated with larger studies. Future clinical trials that incorporate ctDNA clearance in the study design may allow for patient-level, real-time measurement of therapy effectiveness.

In the postoperative context, ctDNA monitoring showed a significant improvement in relapse detection compared with standard-of-care radiologic imaging, demonstrating a significant lead time of 8.7 months (*P* < .001). Of importance, while patients were awaiting radiologic detection, their ctDNA levels increased 5-fold, indicating that tumor burden increases markedly during the 8.7 months of lead time. Current guidelines recommend surveillance after curative CRC surgery.^4,5,10^ Nevertheless, most relapse events are detected too late to be eligible for curative intervention.^11^ The early detection of residual disease by ctDNA analysis may provide an opportunity for earlier radiologic detection (eFigure 10 in the Supplement); potentially, ctDNA status can be used to guide the frequency of radiologic imaging, the optimal scheduling for which is still debated.^12,30^ In addition to detecting residual disease months before radiologic relapse, we also found that ctDNA could inform about the presence of potentially actionable mutations. In the future, ctDNA analysis may allow earlier implementation of targeted therapies in the recurrence setting.

### Limitations

There are potential limitations to our study, including the modest sample size of patients with recurrent CRC and the analysis of multiple patient subsets. In any case, the consistency of the results in the serial analyses documents the robustness, reproducibility, and reliability of the reported findings.

## Conclusions

Our results suggest many potentially paradigm-changing clinical applications of ctDNA in CRC and provide a framework for future clinical trials to investigate the clinical benefits of ctDNA-guided disease management.

## Notes

Supplement. **eMethods 1.** Carcinoembryonic Antigen (CEA) Analysis **eMethods 2.** Sample Collection and DNA Extractions **eMethods 3.** Whole Exome Sequencing (WES) **eMethods 4.** Blood Collection and Plasma Isolation **eMethods 5.** Cell-Free DNA Extraction and Quantification **eMethods 6.** Plasma DNA Libraries and Plasma Multiplex PCR NGS Workflow **eMethods 7.** Plasma Variant Calling **eMethods 8.** Quality Control and Signatera RUO Workflow **eMethods 9.** Time Requirement **eResults 1.** Performance Estimates of the Assay Based on Number of Mutations for Assay Design **eResults 2.** Molecular Profiling of Metastases in Low Shedders **eTable 1.** Clinicopathological Patient Characteristics **eTable 2.** Performance Estimates of the Assay Based on Number of Mutations for Assay Design **eTable 3.** Patient Characteristics and Demographics of Eligible Patients **eTable 4.** Summary of Samples and WES Information **eTable 5.** ctDNA Results for All 795 Plasma Samples **eTable 6.** Recurrence-Free Survival Analysis by Clinicopathological Variables and Post-op ctDNA Status at Day 30 **eTable 7.** Recurrence-Free Survival Analysis by Clinicopathological Variables, Post-op ctDNA, and Post-op CEA Status at First Timepoint Post-ACT **eTable 8.** Matched Tumor and Metastatic WES **eTable 9.** Recurrence-Free Survival Analysis by Clinicopathological Variables and Post-op ctDNA and CEA Status in Surveillance Samples **eTable 10.** Patients With Actionable Mutations Detected in the Primary Tumor **eFigure 1.** Summary of Clinical, Histopathological and Molecular Parameters for All 125 Patients **eFigure 2.** Quality Control Metrics for cfDNA Sequencing Using Multiplex PCR NGS. **eFigure 3.** Detailed ctDNA Results and Disease Course Information **eFigure 4.** Pre-operative Detection of ctDNA and CEA in 122 Stage I-III CRC Patients **eFigure 5.** ctDNA Profiling Results From the 94 Patients Included in the Day 30 ctDNA Analysis **eFigure 6.** ctDNA Profiling Results of the ACT Treated Fraction of Patients (n = 52) Included in the Day 30 ctDNA Analysis **eFigure 7.** ctDNA Profiling Results From the 75 Patients Included in the Longitudinal Post-Definitive-Treatment ctDNA Analysis **eFigure 8.** CEA Profiling Results From the 75 Patients Included in the Post-Definitive-Treatment ctDNA Surveillance Analysis **eFigure 9.** Comparison of Time to Recurrence by CEA and Standard-of-Care CT Imaging **eFigure 10.** Clinical Trial Proposals for Investigating the Clinical Benefit of ctDNA-Guided Post-Operative Management of CRC Patients **eReferences**
