# Supplementary material for: Analysis of Plasma Cell-Free DNA by Ultradeep Sequencing in Patients With Stages I to III Colorectal Cancer
Source: JAMA Oncol. 2019 May 9;5(8):1124–31. doi: 10.1001/jamaoncol.2019.0528 (PMC6512280; doi:10.1001/jamaoncol.2019.0528)
Supplement: Supplement. — eMethods 1. Carcinoembryonic Antigen (CEA) Analysis eMethods 2. Sample Collection and DNA Extractions eMethods 3. Whole Exome Sequencing (WES) eMethods 4. Blood Collection and Plasma Isolation eMethods 5. Cell-Free DNA Extraction and Quantification eMethods 6. Plasma DNA Libraries and Plasma Multiplex PCR NGS Workflow eMethods 7. Plasma Variant Calling eMethods 8. Quality Control and Signatera RUO Workflow eMethods 9. Time Requirement eResults 1. Performance Estimates of the Assay Based on Number of Mutations for Assay Design eResults 2. Molecular Profiling of Metastases in Low Shedders eTable 1. Clinicopathological Patient Characteristics eTable 2. Performance Estimates of the Assay Based on Number of Mutations for Assay Design eTable 3. Patient Characteristics and Demographics of Eligible Patients eTable 4. Summary of Samples and WES Information eTable 5. ctDNA Results for All 795 Plasma Samples eTable 6. Recurrence-Free Survival Analysis by Clinicopathological Variables and Post-op ctDNA Status at Day 30 eTable 7. Recurrence-Free Survival Analysis by Clinicopathological Variables, Post-op ctDNA, and Post-op CEA Status at First Timepoint Post-ACT eTable 8. Matched Tumor and Metastatic WES eTable 9. Recurrence-Free Survival Analysis by Clinicopathological Variables and Post-op ctDNA and CEA Status in Surveillance Samples eTable 10. Patients With Actionable Mutations Detected in the Primary Tumor eFigure 1. Summary of Clinical, Histopathological and Molecular Parameters for All 125 Patients eFigure 2. Quality Control Metrics for cfDNA Sequencing Using Multiplex PCR NGS. eFigure 3. Detailed ctDNA Results and Disease Course Information eFigure 4. Pre-operative Detection of ctDNA and CEA in 122 Stage I-III CRC Patients eFigure 5. ctDNA Profiling Results From the 94 Patients Included in the Day 30 ctDNA Analysis eFigure 6. ctDNA Profiling Results of the ACT Treated Fraction of Patients (n = 52) Included in the Day 30 ctDNA Analysis eFigure 7. ctDNA Profiling Results Fro [file jamaoncol-5-1124-s001.pdf]

## Supplementary Online Content

Reinert T, Henriksen TV, Christensen E, et al. Analysis of plasma cell-free DNA by ultradeep sequencing in patients with stages I to III colorectal cancer. *JAMA Oncol.* Published online May 9, 2019. doi:10.1001/jamaoncol.2019.0528

**eMethods 1.** Carcinoembryonic Antigen (CEA) Analysis
**eMethods 2.** Sample Collection and DNA Extractions
**eMethods 3.** Whole Exome Sequencing (WES)
**eMethods 4.** Blood Collection and Plasma Isolation
**eMethods 5.** Cell-Free DNA Extraction and Quantification
**eMethods 6.** Plasma DNA Libraries and Plasma Multiplex PCR NGS Workflow
**eMethods 7.** Plasma Variant Calling
**eMethods 8.** Quality Control and Signatera RUO Workflow
**eMethods 9.** Time Requirement
**eResults 1.** Performance Estimates of the Assay Based on Number of Mutations for Assay Design
**eResults 2.** Molecular Profiling of Metastases in Low Shedders
**eTable 1.** Clinicopathological Patient Characteristics
**eTable 2.** Performance Estimates of the Assay Based on Number of Mutations for Assay Design
**eTable 3.** Patient Characteristics and Demographics of Eligible Patients
**eTable 4.** Summary of Samples and WES Information
**eTable 5.** ctDNA Results for All 795 Plasma Samples
**eTable 6.** Recurrence-Free Survival Analysis by Clinicopathological Variables and Post-op ctDNA Status at Day 30
**eTable 7.** Recurrence-Free Survival Analysis by Clinicopathological Variables, Post-op ctDNA, and Post-op CEA Status at First Timepoint Post-ACT
**eTable 8.** Matched Tumor and Metastatic WES
**eTable 9.** Recurrence-Free Survival Analysis by Clinicopathological Variables and Post-op ctDNA and CEA Status in Surveillance Samples
**eTable 10.** Patients With Actionable Mutations Detected in the Primary Tumor
**eFigure 1.** Summary of Clinical, Histopathological, and Molecular Parameters for All 125 Patients
**eFigure 2.** Quality Control Metrics for cfDNA Sequencing Using Multiplex PCR NGS.
**eFigure 3.** Detailed ctDNA Results and Disease Course Information
**eFigure 4.** Pre-operative Detection of ctDNA and CEA in 122 Stage I-III CRC Patients
**eFigure 5.** ctDNA Profiling Results From the 94 Patients Included in the Day 30 ctDNA Analysis
**eFigure 6.** ctDNA Profiling Results of the ACT Treated Fraction of Patients (n=52) Included in the Day 30 ctDNA Analysis
**eFigure 7.** ctDNA Profiling Results From the 75 Patients Included in the Longitudinal Post-Definitive-Treatment ctDNA Analysis
**eFigure 8.** CEA Profiling Results From the 75 Patients Included in the Post-Definitive-Treatment ctDNA Surveillance Analysis
**eFigure 9.** Comparison of Time to Recurrence by CEA and Standard-of-Care CT Imaging
**eFigure 10.** Clinical Trial Proposals for Investigating the Clinical Benefit of ctDNA-Guided Post-Operative Management of CRC Patients
**eReferences**

This supplementary material has been provided by the authors to give readers additional information about their work.

© 2019 Reinert T et al. *JAMA Oncology.*

**eMethods 1. Carcinoembryonic Antigen (CEA) Analysis**
CEA analysis was performed on a Cobas e601 platform (Roche), according to the manufacturer's recommendations using 500 µL serum. The threshold levels were set to 4.0 µg/L and 6.0 µg/L for non-smokers and smokers, respectively, as recommended by the analysing hospital. A person who had not smoked for 8 weeks before sample collection was considered a former smoker.

**eMethods 2. Sample Collection and DNA Extractions**
Tumor tissue was collected from all patients, either as fresh frozen (n=102) or as formalin fixed and paraffin embedded tissue (FFPE) (n=27). Four patients presented with synchronous CRCs; from these patients, tissues from both tumors were collected. From three patients with relapse, metastatic tissue was also collected. Constitutional DNA matching all patients was extracted from peripheral blood leukocytes.
Primary fresh frozen or formalin fixed paraffin embedded (FFPE) tissue samples were estimated to have a median pathological tumor cellularity of 50% (range 20-90%). DNA was extracted using the Puregene DNA purification kit (Gentra Systems) or using the QiAamp DNA FFPE tissue kit (Qiagen).

**eMethods 3. Whole Exome Sequencing (WES)**
WES was performed by the Department of Molecular Medicine on matched tumor DNA (derived from primary fresh frozen and FFPE tissue) and buffy coat DNA (eTable 4) as previously described [1]. Libraries of tumor and matching germline DNA were prepared using 100-500 ng DNA and captured by SeqCapEZ MedExomePlusV1_hg19 panel (Roche) with a total (primary and capture) target size of 72 megabases. The MedExomePlusV1_hg19 panel was customized with 1042 SNP sites located throughout the genome. FastQ files were prepared using bcl2fastq2 (v2.20.0.422) and quality checked using FastQC (v0.11.5). Adapters were removed using Trim Galore! (v 0.4.1) The trimmed tumor and germline samples were treated according to the GATK (v3.7) best practices. Reads were mapped to the hg19 reference genome using bwa-mem (v0.7.12) and PCR duplicates were marked for filtering in the downstream analysis using Picard MarkDuplicates (v2.0.1). Local realignment around Indels and recalibration of base quality scores were then performed using GATK IndelRealigner and GATK BaseRecalibrator, respectively. As a quality control for all samples captured by the MedExomePlus panel, tumor and germline alignments were checked using allele counts for 1042 ID SNP sites. Briefly, genotype analysis of the 1042 fingerprint SNP sites served as a control for DNA contamination or sample and/or barcode mix-ups. The analysis was performed as previously described.[2] Samples were flagged and eliminated in situations where the average minor allele frequency at homozygous sites in a patient-matched normal was observed to be >1%. Samples with more than 55% heterozygous SNP sites were eliminated as such percentages indicate large-scale contamination of DNA from another individual. [2] SNVs and Indels were called using MuTect2 with information from COSMIC (v84) and dbSNP (v138). Default settings were applied, however, the threshold for maximum alternate alleles in the germline was raised. A custom filter selecting variants only vastly more present in the tumor and in regions with low noise, was subsequently applied as described earlier [3]. Furthermore, variants identified, but filtered, using MuTect2 were rescued if identified with high confidence using VarScan2 (2.4.1). All variants passing the applied filters were subjected to analysis on the activity of mutational signatures. Variants were initially loaded into a VRanges object and the sequence context was subsequently extracted using the SomaticSignatures R package [4,5]. De novo extraction of mutation signatures was not applied due to the size of the cohort. The mutational profiles identified in our samples were instead projected onto the known COSMIC signatures using the MutationalPatterns R package [6,7]. We identified signatures 1, 6 and 10, as previously reported for CRC[8], but also a strong activity of signature 15 and 18 in a subset of samples.

**eMethods 4. Blood Collection and Plasma Isolation**
Blood samples were collected in K2-EDTA 10 ml tubes (Becton Dickinson) at Aarhus University Hospital. All samples were processed within 2 hours of collection by double centrifugation of the blood at RT, first for 10 minutes at 3000g, followed by centrifugation of plasma for 10 minutes at 3000g. Plasma was aliquoted into 5 mL cryotubes and stored at −80°C.

**eMethods 5. Cell-Free DNA Extraction and Quantification**
Up to 10 ml of plasma per case was used for this study (range, 2–10 mL; median 8.5 mL) and cfDNA was extracted using the QIAamp Circulating Nucleic Acid kit (Qiagen) and eluted into 50 µL DNA Suspension Buffer (Sigma). Each cfDNA sample was quantified by Quant-iT High Sensitivity dsDNA Assay Kit (Invitrogen). In 125 patients, cfDNA was isolated from a total of 795 serial plasma samples.

© 2019 Reinert T et al. *JAMA Oncology.*

**eMethods 6. Plasma DNA Libraries and Plasma Multiplex-PCR NGS Workflow**

Up to 66 ng (20,000 genome equivalents) of cfDNA from each plasma sample was used as input into library preparation. The cfDNA was end-repaired, A-tailed, and ligated with custom adapters, as previously described [9]. The purified ligation product was amplified (20 cycles) and purified using Ampure XP beads (Agencourt/Beckman Coulter). Patient-specific somatic variants were identified by analyses of primary tumour and matched normal WES samples for all patients. Clonality of variants was inferred based on the estimated proportion of cancer cells harboring the variant as described in McGranahan et al [10]. Note that clonality inference from samples with low tumor cell fraction is limited due to a fairly flat distribution of variant allele frequency. Observed VAF in tissue and sequence context of variants were used to prioritize somatic SNVs and short INDELs identified for each tumour expected to have a low plasma background mutation rate. The Signatera amplicon design pipeline was used to generate PCR primer pairs for the given set of variants. Variant prioritization takes into account several factors such as clonality, allele fractions, and SNV type. Design of the 16-plex PCR assays is based on Natera's optimized set of parameters which includes selecting non-interacting set of primer-pairs. Each variant gets its own PCR primers and amplicon in order to ensure targets aren't sharing reads. For each patient, 16 highly ranked compatible amplicons were selected for the custom patient-specific panel. The PCR primers were ordered from Integrated DNA Technologies. An aliquot of each library was used as input into the associated patient-specific 16-plex PCR reaction. Samples were amplified using the patient-specific assay and barcoded, followed by product pooling. Sequencing was performed on an Illumina HiSeq 2500 Rapid Run with 50 cycles of paired-end reads using the Illumina Paired End v2 kit with an average read depth of >105,000X per amplicon. All paired-end reads were merged using Pear software[11]. Bases that did not match in forward and reverse reads or that had a low quality score were filtered out to minimize sequencing errors. Merged reads were mapped to the hg19 reference genome with Novoalign version 2.3.4 (http://www.novocraft.com/). Amplicons with less than 5000x sequencing coverage were excluded from analyses and samples with less than 8 passing amplicons failed sequencing coverage QC.

**eMethods 7. Plasma Variant Calling**

A large set of negative control samples (~1000) were pre-processed to build a background error model. For each target variant using mutant and reference alleles depth of read, a confidence score was calculated on the basis of the error model, as described in Abbosh et al. 2017 [9]. As previously, a plasma sample with at least 2 variants with a confidence score above a predefined algorithm threshold was defined as ctDNA positive [8]. Signatera technology allows confident detection down to 0.03% VAF and lower VAF levels are also observable in many cases [12].

**eMethods 8. Quality Control and Signatera RUO Workflow**

For the Signatera RUO plasma workflow, a strictly-controlled semi-automated lab process was performed by trained personnel with signed-off SOPs and witnessing. The process, reagent, and equipment information were captured electronically and uploaded to a database with built-in integrity checks. A total out of 795 plasma samples 793 (99%) passed our sample QC process. In order to track sample integrity, SNP tracers (45 frequent SNPs) were used to measure concordance between a patient's samples. A genotyping concordance score was calculated for all plasma samples by comparing to their matched normal tissue genotypes. Samples were considered to be from different patients if less than 85% of their SNPs showed identical genotype calls. The genotype calls are produced by Natera proprietary algorithm that fits a distribution to observed reads, calculates the probabilities of a particular genotype, and assigns the highest probability genotype to a SNP. A total of 421 sequencing samples including all plasma samples from recurrence patients were tested by SNP tracer to check the concordance between the plasma sample and its corresponding tissue biopsy. All but one plasma sample passed the concordance QC (eFigure 2).

**eMethods 9. Time Requirement**

The turnaround time for Signatera is broken down into the first blood collection timepoint and all subsequent blood collection timepoints. The first blood collection timepoint entails the following steps: a) tumor tissue and whole blood-based personalized assay design and b) plasma processing, ctDNA analysis, and reporting. The turnaround time for timepoint 1 is less than 4 weeks. All subsequent blood collection timepoints involve only the plasma processing, ctDNA analysis, and reporting, the turnaround time for which is 1-2 weeks. Detailed steps for each of the timepoints are listed below.

**Timepoint 1**

The clinician will be provided with a blood collection kit (composed of two Streck blood tubes and an EDTA tube) simultaneously with a tissue collection kit. The tissue collection kit and EDTA tube will be used in Step 1. Plasma from the two Streck tubes gets isolated and cfDNA is extracted. cfDNA gets used in Step 2.

© 2019 Reinert T et al. *JAMA Oncology.*

**Step 1**
The clinician is expected to send in the patient's tumor block or FFPE slides from surgery/biopsy along with their blood tubes. It takes approximately 2.5 weeks from the time of tissue block or slides receipt for the following steps: tumor tissue block or slide pathology (sectioning, slide processing), DNA extraction from both tumor FFPE slides and matching whole blood, whole exome sequencing (WES) of tumor and matched normal DNA, WES analysis, variant selection and personalized assay design, primer ordering, primer receipt, and primer pooling. This patient-specific Signatera primer pool/assay is stored and gets accessioned when subsequent blood samples from the same patient are received for ctDNA testing.

**Step 2**
As soon as the personalized assay has been designed in step 1, the cfDNA can be made into a universal adapter library. An aliquot of the library is combined with the patient-specific multiplex primer pool for the mPCR assay where only the targets of interest are amplified. The mPCR amplicon product is barcoded, pooled, and sequenced. Sequencing data goes through QC followed by the ctDNA calling algorithm. The report is then issued.

**Subsequent blood collection timepoints**
For all subsequent blood collection timepoints, the clinician is sent only the blood collection kit with the 2 Streck tubes. Plasma from the two Streck tubes gets isolated and cfDNA extracted. This cfDNA then gets used as described above in Step 2 with the patient-specific Signatera assay (originally designed in Step 1)."

© 2019 Reinert T et al. *JAMA Oncology.*

**eResults 1. Performance Estimates of the Assay Based on Number of Mutations for Assay Design**

To demonstrate the effect of the number of mutations tracked by the patient-specific assay on the sensitivity of ctDNA detection, we performed a simulation analysis by using smaller subsets of mutations from the patient-specific assays run in this study, and recalculated the sensitivity of ctDNA detection for pre-surgery samples (n=122) and relapse patients (n=16). Smaller number of mutations (<16) were chosen following the same prioritization and variant selection strategy as employed for the original 16-plex assays. Simulation results for ctDNA detection when using 2,4,8, or 16 of tumor mutations in the customized assays is presented in eTable 2; as expected, the sensitivity of the assay improves with an increase in the number of personalized mutations included in the assay.

**eResults 2. Molecular Profiling of Metastases in Low Shedders**

For two recurrence patients (ID 20 and 24), longitudinal analysis detected no ctDNA post-op (eFigure 7). We analyzed the same amount of plasma for these two patients, as for the other patients. A possible sample swap can be rejected after SNP tracer analysis, which confirmed that for each patient, the tumor, buffy coat and plasma samples came from the same individual. We performed WES of the metastatic recurrence lesions for the two patients, and confirmed that the mutations selected for plasma profiling were present in the metastases (eTable 8). WES was also performed on the metastases from patient 77. For this patient, the ctDNA longitudinal analysis did not detect ctDNA until after recurrence had been detected by radiological imaging (eFigure 7). Again, WES confirmed that the mutations selected for plasma profiling were present in the metastasis. Accordingly, we conclude that the reason for our negative post-op findings was ctDNA levels below detection level and not that the selected markers were non-informative.

© 2019 Reinert T et al. *JAMA Oncology.*

## eTable 1. Clinicopathological Patient Characteristics

| Patient ID | Age | Gender | Primary tumor site | Primary tumor diameter , mm | UICC stage | TNMV | ACT | First relapse after OP month | Relapse site | Relapse treatment | MSS/MSI status | Perineu ral invasio n | Number of lymph nodes in resected specimen | Microscopic radical resection | Ileus | Anastomotic leakage | Tumor perforation | WHO perform ance status |
|---|---|---|---|---|---|---|---|---|---|---|---|---|---|---|---|---|---|---|
| 1 | 68 | Male | Colon | 52 | II | T3N0MxV0 | No | NA[a] | | | MSS | No | 41 | Yes | No | No | No | 1 |
| 2 | 70 | Male | Colon | 54 | II | T3N0MxV0 | No | NA | | | MSS | Yes | 31 | Yes | No | No | No | 1 |
| 3 | 67 | Female | Colon | 32 | II | T3N0MxV0 | No | NA | | | MSS | No | 24 | Yes | No | No | No | 1 |
| 4 | 64 | Male | Colon | 35 | III | T3N1MxV0 | Yes | NA | | | MSS | No | 39 | Yes | N/A[d] | No | No | 1 |
| 5 | 77 | Male | Colon | 70 | II | T3N0MxV0 | No | NA | | | MSS | No | 34 | Yes | No | No | No | 1 |
| 6 | 75 | Male | Colon | 65 | II | T3N0MxV0 | No | NA | | | MSS | No | 23 | Yes | No | No | No | 1 |
| 7 | 70 | Male | Colon | 29 | II | T3N0M0V0 | No | NA | | | MSS | No | 28 | Yes | No | No | No | 1 |
| 8 | 65 | Female | Colon | 42 | III | T3N1M0V0 | Yes | NA | | | MSS | No | 32 | Yes | N/A | No | No | 1 |
| 9 | 50 | Male | Colon | 100 | II | T3N0M0V0 | Yes | NA | | | MSS | No | 51 | Yes | No | N/A[d] | No | 1 |
| 10 | 50 | Female | Colon | 15 | III | T3N1M0V0 | Yes | NA | | | MSS | No | 27 | Yes | N/A | No | No | 1 |
| 11 | 70 | Male | Colon | 40 | III | T3N2M0V1 | Yes | NA | | | MSS | Yes | 27 | Yes | N/A | No | No | 1 |
| 12 | 67 | Female | Colon | 50 | II | T3N0M0V0 | No | NA | | | MSS | No | 41 | Yes | No | No | No | 1 |
| 13 | 66 | Female | Colon | 40 | II | T4N0MxV0 | Yes | NA | | | MSS | Yes | 27 | Yes | No | No | No | 1 |
| 14 | 68 | Female | Colon | 63 | II | T3N0MxV0 | No | NA | | | MSI | Yes | 28 | Yes | No | No | No | 1 |
| 15 | 48 | Male | Colon | 42 | III | T3N2MxV0 | Yes | NA | | | MSS | Yes | 38 | Yes | N/A | No | No | 1 |
| 16 | 68 | Female | Colon | 50 | II | T3N0MxV0 | No | NA | | | MSS | Yes | 28 | Yes | No | No | No | 1 |
| 18 | 67 | Male | Colon | 45 | III | T2N2MxV0 | Yes | 12 | Liver | Surgery | MSS | Yes | 21 | No | N/A | No | No | 1 |
| 19 | 69 | Female | Colon | 58 | II | T3N0M0V0 | No | NA | | | MSI | Yes | 28 | Yes | No | No | No | 2 |
| 20 | 73 | Female | Rectal | 55 | III | T3N1M0V2 | Yes | 12 | Lung | Surgery | MSS | Yes | 21 | Yes | N/A | No | No | 1 |
| 21 | 68 | Male | Colon | 55 | III | T3N1M0V0 | Yes | NA | | | MSS | Yes | 26 | Yes | N/A | No | No | 1 |
| 22 | 67 | Female | Rectal | 60 | III | T3N1M0V1 | Yes | NA | | | MSS | Yes | 36 | Yes | N/A | No | No | 1 |
| 23 | 69 | Male | Rectal | 53 | III | T4N1M0V1 | Yes | NA | | | MSS | Yes | 41 | Yes | N/A | No | No | 1 |
| 24 | 82 | Male | Colon | 77 | III | T3N1M0V1 | Yes | 13 | Liver | RFA liver | MSS | Yes | 33 | Yes | N/A | No | No | 1 |
| 25 | 72 | Female | Colon | 95 | II | T3N0M0V0 | No | NA | | | MSI | Yes | 62 | Yes | No | No | No | 1 |
| 26 | 66 | Male | Colon | 82 | III | T4N1M0V0 | Yes | NA | | | MSS | Yes | 42 | Yes | N/A | No | No | 1 |
| 27 | 68 | Male | Rectal | 37 | III | T3N1M0V0 | Yes | NA | | | MSS | Yes | 27 | Yes | N/A | No | No | 1 |
| 28 | 46 | Female | Colon | 66 | III | T4N2M0V1 | Yes | 31 | Multiple | Chemotherapy | MSS | Yes | 32 | Yes | N/A | No | No | 1 |
| 29 | 65 | Female | Rectal | 37 | III | T3N1M0V1 | Yes | 12 | Lung | Chemotherapy | MSS | Yes | 15 | No | N/A | No | No | 1 |
| 30 | 52 | Female | Colon | 47 | III | T3N2M0V2 | Yes | 9 | Bone | Chemotherapy | MSS | Yes | 28 | No | N/A | No | No | 1 |

© 2019 Reinert T et al. *JAMA Oncology.*

| eTable 1. Clinicopathological Patient Characteristics | | | | | | | | | | | | | | | | | | |
|---|---|---|---|---|---|---|---|---|---|---|---|---|---|---|---|---|---|---|
| **Patient ID** | **Age** | **Gender** | **Primary tumor site** | **Primary tumor diameter, mm** | **UICC stage** | **TNMV** | **ACT** | **First relapse after OP month** | **Relapse site** | **Relapse treatment** | **MSS/MSI status** | **Perineural invasion** | **Number of lymph nodes in resected specimen** | **Microscopic radical resection** | **Ileus** | **Anastomotic leakage** | **Tumor perforation** | **WHO performance status** |
| 31 | 54 | Male | Colon | 25 | III | T3N1M0V0 | Yes | NA | | | MSS | Yes | 28 | Yes | N/A | No | No | 1 |
| 33 | 47 | Male | Colon | 170 | III | T3N1M0V1 | Yes | NA | | | MSI | NA | 81 | Yes | N/A | No | No | 1 |
| 34 | 69 | Male | Colon | 37 | II | T3N0M0V1 | Yes | 31 | Liver | Palliative | MSS | Yes | 35 | Yes | No | No | No | 1 |
| 35 | 68 | Male | Colon | 38 | III | T3N1M0V0 | Yes | NA | | | MSS | Yes | 21 | Yes | N/A | No | No | 1 |
| 36 | 70 | Male | Colon | 47 | III | T4N2M0V1 | Yes | NA | | | MSS | Yes | 37 | Yes | N/A | No | No | 1 |
| 37 | 75 | Male | Colon | 46 | II | T3N0M0V0 | No | 6 | Liver | RFA liver | MSS | Yes | 30 | Yes | No | No | No | 1 |
| 38 | 49 | Female | Colon | 41 | II | T3N0M0V0 | No | NA | | | MSI | Yes | 27 | Yes | No | No | No | 1 |
| 39 | 74 | Male | Colon | 115 | III | T4N2M0V1 | Yes | NA | | | MSS | Yes | 27 | No | N/A | No | No | 1 |
| 40 | 69 | Male | Colon | 85 | III | T3N2M0V1 | Yes | NA | | | MSS | Yes | 23 | No | N/A | No | No | 1 |
| 41 | 70 | Male | Colon | 67 | II | T3N0M0V0 | No | NA | | | MSS | Yes | 35 | Yes | No | No | No | 1 |
| 42 | 60 | Male | Colon | 73 | III | T4N2MxV1 | Yes | 14 | Carcinosis | Chemotherapy | MSS | Yes | 36 | No | N/A | No | No | 1 |
| 43 | 48 | Male | Rectal | 51 | II | T3N0MxV0 | No | NA | | | MSS | No | 40 | Yes | Yes | No | No | 1 |
| 44 | 76 | Female | Colon | 70 | II | T4N0M0V0 | Yes | NA | | | MSS | No | 19 | Yes | No | No | No | 1 |
| 45 | 83 | Female | Colon | 40 | II | T3N0MxV0 | No | NA | | | MSI | No | 27 | Yes | No | No | No | 2 |
| 46 | 72 | Female | Colon | 70 | III | T4N2MxV1 | Yes | NA | | | MSI | No | 37 | No | N/A | No | Yes | 2 |
| 47 | 73 | Female | Colon | 7 | II | T3N0MxV0 | No | NA | | | MSS | No | 37 | Yes | No | No | No | 2 |
| 48 | 91 | Female | Colon | 55 | III | T3N1M0V1 | No | NA | | | MSI | | 32 | Yes | N/A | No | No | 2 |
| 49 | 80 | Female | Colon | 20 | III | T4N1M0V0 | Yes | NA | | | MSS | Yes | 19 | No | N/A | No | No | 1 |
| 50 | 64 | Male | Colon | 83 | II | T4N0M0V2 | Yes | NA | | | MSS | Yes | 40 | Yes | No | No | No | 1 |
| 51 | 60 | Male | Colon | 70 | II | T3N0M0V0 | No | NA | | | MSS | Yes | 40 | Yes | No | Yes | No | 1 |
| 52 | 81 | Male | Colon | 30 | II | T3N0M0V0 | No | NA | | | MSS | No | 17 | Yes | No | No | No | 2 |
| 53 | 80 | Female | Colon | 32 | III | T3N1M0V0 | No | NA | | | MSS | Yes | 18 | No | N/A | No | No | 2 |
| 54 | 66 | Male | Colon | 40 | III | T3N1M0V0 | No | NA | | | MSS | Yes | 17 | Yes | N/A | Yes | No | NA |
| 55 | 78 | Male | Colon | 40 | II | T3N0M0V0 | No | NA | | | MSS | Yes | 18 | Yes | No | No | No | 2 |
| 57 | 50 | Male | Colon | 45 | II | T3N0M0V0 | No | NA | | | MSS | Yes | 32 | Yes | No | No | No | 1 |
| 58 | 77 | Male | Colon | 52 | II | T4N0M0V0 | No | NA | | | MSI | Yes | 24 | Yes | No | No | No | 1 |
| 59 | 51 | Female | Colon | 20 | I | T2N0M0V0 | No | NA | | | MSS | No | 16 | Yes | N/A | No | No | 1 |
| 60 | 70 | Female | Colon | 32 | II | T3N0M0V0 | No | NA | | | MSS | Yes | 14 | Yes | No | No | No | 1 |
| 61 | 67 | Male | Colon | 24 | II | T3N0M0V0 | No | NA | | | MSS | Yes | 20 | Yes | No | No | No | 1 |
| 62 | 55 | Male | Colon | 30 | III | T4N1M0V1 | Yes | NA | | | MSS | No | 19 | Yes | N/A | No | No | 1 |

© 2019 Reinert T et al. *JAMA Oncology.*

## eTable 1. Clinicopathological Patient Characteristics

| Patient ID | Age | Gender | Primary tumor site | Primary tumor diameter, mm | UICC stage | TNMV | ACT | First relapse after OP month | Relapse site | Relapse treatment | MSS/MSI status | Perineural invasion | Number of lymph nodes in resected specimen | Microscopic radical resection | Ileus | Anastomotic leakage | Tumor perforation | WHO performance status |
|---|---|---|---|---|---|---|---|---|---|---|---|---|---|---|---|---|---|---|
| 63 | 82 | Male | Colon | 40 | III | T3N1MxV0 | No | NA | | | MSI | No | 27 | Yes | N/A | No | No | 1 |
| 64 | 71 | Female | Colon | 75 | III | T3N1MxV1 | Yes | NA | | | MSS | No | 19 | Yes | N/A | No | No | 1 |
| 65 | 72 | Male | Colon | 55 | III | T4N1MxV1 | Yes | NA | | | MSS | Yes | 31 | No | N/A | No | No | 1 |
| 66 | 72 | Male | Colon | 90 | III | T3N2MxV0 | Yes | NA | | | MSS | No | 49 | Yes | N/A | No | No | 1 |
| 67 | 71 | Male | Colon | 35 | III | T4N1MxV0 | Yes | NA | | | MSS | No | 31 | Yes | N/A | No | No | 2 |
| 68 | 83 | Female | Colon | 75 | III | T3N2MxV1 | No | 13 | Carcinosis | Chemotherapy | MSS | No | 24 | Yes | N/A | No | No | 1 |
| 69 | 56 | Female | Colon | 25 | III | T3N1MxV1 | Yes | NA | | | MSS | No | 18 | Yes | N/A | No | No | 2 |
| 70 | 66 | Male | Colon | 20 | III | T3N2MxV0 | Yes | NA | | | MSS | No | 37 | Yes | N/A | No | No | 1 |
| 71 | 77 | Male | Colon | 90 | III | T3N1MxV0 | Yes | NA | | | MSI | No | 26 | Yes | N/A | No | No | 2 |
| 72 | 49 | Female | Colon | 41 | III | T3N1MxV0 | Yes | NA | | | MSS | No | 16 | Yes | N/A | No | No | 1 |
| 73 | 43 | Female | Colon | 64 | III | T4N2MxV1 | Yes | NA | | | MSS | No | 30 | Yes | N/A | No | No | 1 |
| 74 | 72 | Male | Colon | 18 | III | T3N2MxV0 | Yes | NA | | | MSS | No | 25 | Yes | N/A | No | No | 2 |
| 75 | 48 | Female | Colon | 74 | III | T3N1MxV0 | Yes | 11 | Liver | Surgery | MSS | No | 48 | Yes | N/A | No | No | 2 |
| 76 | 75 | Male | Colon | 73 | III | T3N2MxV0 | Yes | NA | | | MSS | No | 26 | Yes | N/A | No | No | 2 |
| 77 | 71 | Female | Colon | 52 | III | T3N1MxV1 | Yes | 13 | Lung | Surgery | MSS | Yes | 280 | Yes | N/A | No | No | 2 |
| 78 | 64 | Female | Colon | 50 | III | T4N2MxV0 | Yes | NA | | | MSS | No | 23 | Yes | N/A | No | No | 1 |
| 79 | 50 | Female | Colon | 40 | III | T3N1MxV0 | Yes | 15 | Liver | Surgery/RFA | MSS | No | 20 | Yes | N/A | No | No | 1 |
| 80 | 73 | Female | Colon | 82 | III | T4N1MxV0 | Yes | NA | | | MSS | No | 47 | Yes | N/A | No | No | 1 |
| 81 | 58 | Female | Colon | 23 | III | T3N2MxV0 | Yes | NA | | | MSS | No | 17 | Yes | N/A | No | No | 1 |
| 82 | 50 | Female | Colon | 30 | III | T3N2M0V1 | Yes | 11 | LN[b] (throat) | Surgery | MSS | Yes | 29 | Yes | N/A | No | No | NA |
| 83 | 62 | Female | Colon | 70 | III | T3N2MxV0 | Yes | NA | | | MSS | Yes | 23 | Yes | N/A | No | No | 2 |
| 84 | 65 | Female | Colon | 100 | III | T3N1MxV2 | Yes | NA | | | MSS | No | 24 | Yes | N/A | No | No | 1 |
| 85 | 61 | Female | Colon | 35 | III | T3N2MxV0 | Yes | 11 | Liver & lung | None | MSS | No | 27 | Yes | N/A | No | No | 1 |
| 86 | 79 | Male | Colon | 90 | III | T3N2MxV1 | Yes | NA | | | MSS | No | 30 | Yes | N/A | No | No | 1 |
| 87 | 70 | Male | Colon | 145 | III | T3N1MxV2 | Yes | NA | | | MSS | No | 17 | Yes | N/A | No | No | 3 |
| 88 | 64 | Male | Colon | 60 | III | T3N2MxV1 | Yes | NA | | | MSS | No | 47 | Yes | N/A | No | No | 1 |
| 89 | 75 | Male | Colon | 40 | III | T3N2MxV0 | No | 17 | Lung | Surgery | MSS | No | 12 | Yes | N/A | No | No | 3 |
| 90 | 87 | Female | Colon | 62 | III | T3N1MxV0 | No | NA | | | MSS | | 21 | No | Yes | No | No | 3 |
| 91 | 69 | Male | Colon | 70 | III | T3N1MxV0 | Yes | NA | | | MSS | No | 15 | Yes | N/A | No | No | 2 |
| 92 | 73 | Female | Colon | 100 | III | T4N2MxV0 | Yes | 14 | Local | Surgery | MSI | No | 18 | No | N/A | No | No | 2 |

© 2019 Reinert T et al. *JAMA Oncology.*

## eTable 1. Clinicopathological Patient Characteristics

| Patient ID | Age | Gender | Primary tumor site | Primary tumor diameter, mm | UICC stage | TNMV | ACT | First relapse after OP month | Relapse site | Relapse treatment | MSS/MSI status | Perineural invasion | Number of lymph nodes in resected specimen | Microscopic radical resection | Ileus | Anastomotic leakage | Tumor perforation | WHO performance status |
|---|---|---|---|---|---|---|---|---|---|---|---|---|---|---|---|---|---|---|
| 93 | 81 | Male | Colon | 80 | III | T4N2MxV1 | Yes | NA | | | MSS | No | 28 | Yes | N/A | No | No | 2 |
| 95 | 70 | Male | Colon | 30 | I | T2N0MxV0 | No | NA | | | MSS | No | 27 | Yes | N/A | No | No | 1 |
| 96 | 64 | Male | Colon | 40 | III | T4N2M0V1 | Yes | NA | | | MSS | Yes | 33 | Yes | N/A | No | No | 1 |
| 97 | 67 | Female | Colon | 60 | II | T4N0MxV0 | Yes | NA | | | MSS | No | 15 | Yes | No | No | No | 1 |
| 98 | 78 | Male | Colon | 30 | II | T3N0MxV0 | No | NA | | | MSI | No | 15 | Yes | Yes | No | No | 2 |
| 99 | 79 | Male | Colon | 40 | III | T4N2MxV0 | No | 15 | Multiple | Palliative | MSS | No | 30 | Yes | N/A | No | No | 1 |
| 100 | 59 | Female | Colon | 100 | II | T4N0MxV1 | No | NA | | | MSS | Yes | 12 | Yes | No | No | No | 2 |
| 101 | 72 | Male | Colon | 40 | II | T3N0MxV2 | No | NA | | | MSS | No | 34 | Yes | No | No | No | 2 |
| 102 | 69 | Male | Colon | 35 | I | T2N0MxV0 | No | NA | | | MSS | No | 13 | Yes | N/A | No | No | 1 |
| 103 | 70 | Male | Colon | 100 | III | T3N1MxV1 | Yes | 12 | Multiple | None | MSS | No | 38 | No | N/A | No | No | 3 |
| 104 | 50 | Male | Colon | 30 | III | T3N1MxV2 | Yes | 12 | Liver | RFA liver | MSS | No | 24 | NA | N/A | No | No | NA |
| 105 | 72 | Male | Colon | 70 | II | T3N0MxV1 | No | NA | | | MSS | No | 36 | Yes | No | No | No | 2 |
| 106 | 70 | Female | Colon | 25 | I | T2N0MxV0 | No | NA | | | MSI | No | 21 | Yes | N/A | No | No | 1 |
| 107 | 75 | Male | Colon | 30 | III | T3N2MxV0 | Yes | NA | | | MSS | No | 17 | Yes | N/A | No | No | 1 |
| 108 | 72 | Female | Colon | 61 | II | T3N0MxV0 | No | 12 | Liver | RFA liver | MSS | No | 12 | Yes | No | No | No | 3 |
| 109 | 74 | Male | Colon | 50 | II | T3N0MxV0 | No | NA | | | MSS | Yes | 24 | Yes | No | No | No | 2 |
| 110 | 67 | Male | Colon | 35 | I | T2N0MxV0 | No | NA | | | MSS | No | 20 | Yes | N/A | No | No | 2 |
| 111 | 52 | Male | Colon | 45 | III | T3N2M0V1 | Yes | NA | | | MSS | Yes | 31 | No | N/A | No | Yes | 1 |
| 112 | 61 | Male | Colon | 40 | III | T3N1MxV1 | Yes | NA | | | MSS | Yes | 25 | No | N/A | No | No | 1 |
| 113 | 78 | Male | Colon | 70 | III | T3N1MxV1 | No | NA | | | MSS | No | 22 | Yes | N/A | No | No | 1 |
| 114 | 79 | Male | Colon | 60 | III | T3N1MxV0 | Yes | NA | | | MSS | | 33 | Yes | N/A | No | No | 1 |
| 115 | 78 | Male | Colon | 35 | III | T3N2M0Vx | Yes | NA | | | MSS | No | 24 | No | N/A | No | No | 2 |
| 116 | 75 | Female | Colon | 70 | III | T4N1MxV0 | Yes | NA | | | MSS | No | 31 | Yes | N/A | No | No | 1 |
| 117 | 84 | Female | Colon | 56 | III | T3N1MxV0 | Yes | NA | | | MSS | No | 19 | No | N/A | No | No | 2 |
| 118 | 69 | Male | Colon | 80 | III | T4N2MxV0 | Yes | NA | | | MSI | No | 25 | Yes | N/A | No | No | 1 |
| 119 | 69 | Female | Colon | 60 | III | T4N1MxV1 | Yes | 12 | Local | HIPEC | MSS | Yes | 29 | No | N/A | No | Yes | 1 |
| 120 | 61 | Female | Colon | 50 | III | T3N2MxV1 | Yes | NA | | | MSI | No | 31 | Yes | N/A | No | No | 1 |
| 121 | 81 | Female | Colon | 20 | III | T3N1MxV0 | No | NA | | | MSS | No | 14 | Yes | N/A | No | No | 2 |
| 122 | 76 | Male | Colon | 50 | III | T3N1MxV1 | Yes | NA | | | MSS | Yes | 34 | Yes | N/A | No | No | 1 |
| 123 | 59 | Male | Colon | 50 | III | T4N1MxV0 | Yes | NA | | | MSS | Yes | 24 | Yes | N/A | No | No | 1 |

© 2019 Reinert T et al. *JAMA Oncology.*

## eTable 1. Clinicopathological Patient Characteristics

| Patient ID | Age | Gender | Primary tumor site | Primary tumor diameter, mm | UICC stage | TNMV | ACT | First relapse after OP month | Relapse site | Relapse treatment | MSS/MSI status | Perineural invasion | Number of lymph nodes in resected specimen | Microscopic radical resection | Ileus | Anastomotic leakage | Tumor perforation | WHO performance status |
|---|---|---|---|---|---|---|---|---|---|---|---|---|---|---|---|---|---|---|
| 124 | 71 | Female | Colon | 47 | III | T3N2MxV2 | Yes | 12 | Multiple | Palliative | MSS | No | 41 | NA | N/A | No | No | 2 |
| 125 | 78 | Female | Colon | 40 | II | T4N0MxV0 | No | 12 | Lung | Palliative | MSS | No | 31 | Yes | No | No | No | 1 |
| 126 | 71 | Male | Colon | 60 | II | T3N0MxV0 | No | NA | | | MSS | No | 36 | Yes | No | No | No | 1 |
| 127 | 67 | Male | Colon | 30 | II | T3N0MxV0 | No | NA | | | MSI | No | 23 | Yes | No | No | No | 1 |
| 128 | 51 | Male | Colon | 8 | III | T4N1M0V0 | Yes | NA | | | MSI | Yes | 54 | NA | N/A | No | Yes | 2 |
| 130 | 66 | Male | Colon | 60 | III | T4N2M0V0 | Yes | NA | | | MSS | Yes | 19 | Yes | N/A | No | No | 1 |

[a]Not applicable
[b]Lymph node
[c]Hyperthermic intraperitoneal chemotherapy
[d]Not available

© 2019 Reinert T et al. *JAMA Oncology.*

| eTable 2. Performance Estimates of the Assay Based on Number of Mutations for Assay Design | | | | |
|---|---|---|---|---|
| | **Tracking 2 mutations** | **Tracking 4 mutations** | **Tracking 8 mutations** | **Tracking 16 mutations (as reported in this study)** |
| Relapse prediction (out of n=16 patients) | 7 (44%) | 13 (81%) | 13 (81%) | 14 (88%) |
| Pre-operation ctDNA positive samples (out of n=122) | 58 (48%) | 82 (67%) | 96 (79%) | 108 (89%) |

© 2019 Reinert T et al. *JAMA Oncology.*

| eTable 3. Patient Characteristics and Demographics of Eligible Patients | | |
|---|---|---|
| Patients, n | | 125 |
| Cancers, n | | 129[a] |
| Age (years), median (range) | | 69.9 (43.3-91) |
| Gender, n (%) | Female | 52 (41.6) |
| | Male | 73 (58.4) |
| Imaging follow-up (months), median (range) | | 12.5, (1.4-38.5) |
| Location, n (%) | Colon | 119 (95.2) |
| | Rectum | 6 (4.8) |
| Pathological UICC stage, n (%) | I | 5 (4) |
| | II | 39 (31.2) |
| | III | 81 (64.8) |
| Histological type, n (%) | Adenocarcinoma | 115 (92) |
| | Mucinous carcinoma | 10 (8.7) |
| Histological grade, n (%) | Moderately differentiated | 96 (76.8) |
| | Poorly differentiated | 19 (15.2) |
| | ND | 10 (8) |
| Adj. therapy by UICC stage, n (%) | I | 0 (0.0) |
| | II | 6 (15.3) |
| | III | 71 (87.7) |
| | Total | 77 (61.6) |
| Relapse by UICC stage, n (%) | I | 0 (0.0) |
| | II | 4 (10.2) |
| | III | 20 (24.7) |
| | IV | 0 (0.0) |
| | Total | 24 (19.2) |
| Relapse site, n (%)[b] | Distant | 23 (95.8) |
| | Local | 1 (4.2) |
| MSS/MSI status, n (%) | MSS | 109 (87.2) |
| | MSI | 20 (16) |
| Smoking, n (%) | Never | 52 (41.6) |
| | Former | 57 (45.6) |
| | Current | 16 (12.8) |

[a]Four patients with synchronous cancers. Details available in eTable 3
[b]Details available in eTable 1

© 2019 Reinert T et al. *JAMA Oncology.*

| eTable 4. Summary of Samples and WES Information. | | | | | | | | | |
|---|---|---|---|---|---|---|---|---|---|
| Patient ID | Source of DNA | Sample type[b] | Input, ng | WES coverage | Ti/Tv ratio | Median insert size (bp) | Percentage bases on target | Capture fold enrichment | # of variants |
| 1 | Primary tumor | FF | 500 | 100.8 | 2.2 | 213 | 53.6 | 41.1 | 261 |
| 2 | Primary tumor | FF | 500 | 94.3 | 2.2 | 233 | 52.9 | 40.2 | 318 |
| 3 | Primary tumor | FF | 500 | 101.8 | 2.2 | 229 | 59 | 40 | 366 |
| 4 | Primary tumor | FF | 500 | 98.4 | 2.2 | 228 | 58.5 | 40 | 462 |
| 5 | Primary tumor | FF | 500 | 167.8 | 2.2 | 237 | 57.5 | 39.1 | 509 |
| 6 | Primary tumor | FF | 500 | 93 | 2.2 | 229 | 52.9 | 40.4 | 388 |
| 7 | Primary tumor | FF | 500 | 91.2 | 2.2 | 244 | 52.5 | 39.4 | 422 |
| 8 | Primary tumor | FF | 500 | 89.9 | 2.3 | 235 | 60.2 | 39.6 | 469 |
| 9 | Primary tumor | FF | 500 | 91.3 | 1.8 | 238 | 52.3 | 39.6 | 11342 |
| 10 | Primary tumor | FF | 500 | 140.4 | 2.2 | 204 | 62.8 | 40.5 | 387 |
| 11 | Primary tumor | FF | 500 | 104.3 | 2.3 | 224 | 66.6 | 40.5 | 448 |
| 12 | Primary tumor | FF | 500 | 106 | 2.3 | 235 | 58.2 | 39.6 | 594 |
| 13 | Primary tumor | FF | 500 | 47.2 | 2.3 | 244 | 65.6 | 37.9 | 593 |
| 14 | Primary tumor | FF | 500 | 106.6 | 2.2 | 239 | 59.9 | 39.4 | 14532 |
| 15 | Primary tumor | FF | 500 | 105.2 | 2.2 | 224 | 58.7 | 40.3 | 366 |
| 16 | Primary tumor | FF | 250 | 122.7 | 2.2 | 208 | 60.3 | 41.3 | 259 |
| 18 | Primary tumor | FF | 500 | 98 | 2.3 | 206 | 68.2 | 41.6 | 448 |
| 19 | Primary tumor | FF | 500 | 100.3 | 2.2 | 237 | 59.8 | 39.5 | 15574 |
| 20 | Primary tumor | FF | 500 | 112.2 | 2.2 | 233 | 65.4 | 39.8 | 428 |
| 20 | Metastasis | FFPE | 500 | 97.5 | 2.2 | 229 | 63.3 | 40 | 435 |
| 21 | Primary tumor | FF | 500 | 117 | 2.3 | 262 | 59.7 | 37.2 | 436 |
| 22 | Primary tumor | FF | 500 | 102.2 | 2.2 | 219 | 58.9 | 40.5 | 423 |
| 23 | Primary tumor | FF | 500 | 104.9 | 2.3 | 212 | 67.6 | 41.3 | 326 |
| 24 | Primary tumor | FF | 500 | 46.5 | 2.3 | 232 | 67.2 | 39.8 | 536 |
| 24 | Metastasis | FFPE | 500 | 107.4 | 2.3 | 162 | 64.2 | 45.1 | 645 |
| 25 | Primary tumor | FF | 500 | 106.1 | 2.1 | 247 | 59.1 | 38.8 | 7116 |
| 26 | Primary tumor | FF | 500 | 150.9 | 2.2 | 218 | 63.5 | 40.6 | 501 |
| 27 | Primary tumor | FF | 500 | 149.2 | 2.3 | 226 | 63.8 | 40.2 | 355 |
| 28 | Primary tumor | FF | 500 | 129.2 | 2.3 | 216 | 64.2 | 40.9 | 492 |
| 29 | Primary tumor | FF | 500 | 86.1 | 2.3 | 219 | 64.5 | 40.8 | 368 |
| 30 | Primary tumor | FF | 500 | 222 | 2.2 | 244 | 56.4 | 39.1 | 589 |
| 31 | Primary tumor | FFPE | 500 | 123.6 | 2.2 | 210 | 26.3 | 41.4 | 380 |
| 33 (S1)a | Primary tumor | FF | 500 | 87.7 | 2.2 | 226 | 64.2 | 40.3 | 7628 |
| 33 (S2)a | Primary tumor | FFPE | 500 | 65.5 | 2.2 | 164 | 42.5 | 44.4 | 5881 |

© 2019 Reinert T et al. *JAMA Oncology.*

| eTable 4. Summary of Samples and WES Information. | | | | | | | | | |
|---|---|---|---|---|---|---|---|---|---|
| **Patient ID** | **Source of DNA** | **Sample type[b]** | **Input, ng** | **WES coverage** | **Ti/Tv ratio** | **Median insert size (bp)** | **Percentage bases on target** | **Capture fold enrichment** | **# of variants** |
| 34 | Primary tumor | FF | 500 | 90.4 | 2.3 | 256 | 59.1 | 38.3 | 261 |
| 35 | Primary tumor | FF | 500 | 186.3 | 2.2 | 245 | 57.4 | 38.7 | 708 |
| 36 | Primary tumor | FF | 500 | 184.4 | 2 | 242 | 57 | 39 | 395 |
| 37 | Primary tumor | FF | 500 | 87.8 | 2.3 | 227 | 58.9 | 40 | 397 |
| 38 | Primary tumor | FF | 500 | 86.9 | 2.2 | 235 | 58.7 | 39.5 | 4657 |
| 39 | Primary tumor | FF | 500 | 179.4 | 2.2 | 242 | 57.7 | 39.1 | 594 |
| 40 | Primary tumor | FF | 500 | 86.4 | 2.3 | 221 | 63.2 | 40.5 | 558 |
| 41 | Primary tumor | FF | 500 | 88.4 | 2.3 | 216 | 63.6 | 40.8 | 347 |
| 42 | Primary tumor | FF | 500 | 94.9 | 2.2 | 200 | 62.2 | 41.9 | 361 |
| 43 | Primary tumor | FF | 500 | 121.3 | 2.2 | 227 | 53.1 | 40.5 | 401 |
| 44 | Primary tumor | FF | 500 | 103.8 | 2.3 | 228 | 61.4 | 40.1 | 434 |
| 45 | Primary tumor | FF | 500 | 107.6 | 2.2 | 228 | 64.4 | 40.2 | 6265 |
| 46 | Primary tumor | FF | 500 | 99 | 2.2 | 254 | 60.1 | 38.7 | 5943 |
| 47 | Primary tumor | FF | 500 | 94.4 | 2.3 | 209 | 65.3 | 41.5 | 551 |
| 48 (S1)a | Primary tumor | FF | 500 | 95 | 2.1 | 253 | 60.7 | 38.8 | 7014 |
| 48 (S2)a | Primary tumor | FFPE | 500 | 108.5 | 2.1 | 197 | 45.6 | 42.3 | 6276 |
| 49 | Primary tumor | FF | 200 | 127.9 | 2 | 228 | 60.1 | 40.1 | 308 |
| 50 | Primary tumor | FF | 500 | 93.1 | 2.3 | 225 | 61.2 | 40.3 | 323 |
| 51 | Primary tumor | FFPE | 500 | 130.7 | 2.2 | 219 | 45.3 | 41 | 525 |
| 52 | Primary tumor | FFPE | 500 | 113 | 2.3 | 187 | 46.2 | 43.5 | 393 |
| 53 | Primary tumor | FF | 500 | 93.7 | 2.3 | 226 | 64.3 | 40.3 | 286 |
| 54 | Primary tumor | FF | 500 | 113.6 | 2.3 | 228 | 66.3 | 40.1 | 479 |
| 55 | Primary tumor | FF | 500 | 161 | 2.2 | 258 | 57 | 38.1 | 342 |
| 57 | Primary tumor | FF | 500 | 85 | 1.8 | 227 | 64.4 | 40.2 | 20961 |
| 58 | Primary tumor | FF | 500 | 79.4 | 2.2 | 224 | 64.4 | 40.4 | 5804 |
| 59 | Primary tumor | FF | 250 | 127.1 | 2.2 | 214 | 59.6 | 40.9 | 410 |
| 60 | Primary tumor | FFPE | 500 | 119.1 | 2.2 | 199 | 45.6 | 42.4 | 314 |
| 61 | Primary tumor | FF | 500 | 93.6 | 2.2 | 237 | 60.6 | 39.7 | 423 |
| 62 | Primary tumor | FFPE | 500 | 120.2 | 2.3 | 149 | 38.8 | 46 | 586 |
| 63 | Primary tumor | FF | 100 | 116.6 | 2.2 | 231 | 61.8 | 38.9 | 7314 |
| 64 | Primary tumor | FF | 500 | 109 | 2.3 | 231 | 65.7 | 39.9 | 404 |
| 65 | Primary tumor | FFPE | 500 | 99.4 | 2.3 | 192 | 42.3 | 42.8 | 501 |
| 66 | Primary tumor | FF | 500 | 105.2 | 2.3 | 234 | 65.8 | 39.8 | 477 |
| 67 | Primary tumor | FF | 500 | 82.1 | 2.3 | 220 | 64.1 | 40.6 | 487 |
| 68 | Primary tumor | FF | 500 | 102 | 2.3 | 233 | 65.8 | 39.8 | 373 |

© 2019 Reinert T et al. *JAMA Oncology.*

| eTable 4. Summary of Samples and WES Information. | | | | | | | | | |
|---|---|---|---|---|---|---|---|---|---|
| Patient ID | Source of DNA | Sample type[b] | Input, ng | WES coverage | Ti/Tv ratio | Median insert size (bp) | Percentage bases on target | Capture fold enrichment | # of variants |
| 69 | Primary tumor | FF | 500 | 97.3 | 2.3 | 219 | 63.2 | 40.6 | 413 |
| 70 | Primary tumor | FFPE | 500 | 150.4 | 2.2 | 159 | 35.1 | 45.4 | 483 |
| 71 | Primary tumor | FF | 500 | 160.4 | 2.1 | 310 | 55.2 | 35.4 | 6173 |
| 72 | Primary tumor | FF | 500 | 126.8 | 2.3 | 229 | 66.1 | 40.1 | 309 |
| 73 | Primary tumor | FF | 500 | 90.3 | 2.3 | 222 | 64.5 | 40.6 | 432 |
| 74 | Primary tumor | FF | 500 | 127.3 | 2.2 | 207 | 61.7 | 41.3 | 1076 |
| 75 | Primary tumor | FF | 500 | 108.8 | 2.2 | 239 | 58.9 | 39.4 | 683 |
| 76 | Primary tumor | FF | 250 | 111.8 | 2.2 | 202 | 59 | 41.5 | 533 |
| 77 | Primary tumor | FF | 500 | 124.5 | 2.2 | 233 | 52.5 | 40.1 | 476 |
| 77 | Metastasis | FFPE | 500 | 118.8 | 2.3 | 196 | 63.8 | 42.7 | 744 |
| 78 | Primary tumor | FF | 500 | 94.2 | 2.3 | 219 | 67.2 | 41 | 328 |
| 79 | Primary tumor | FF | 500 | 101.2 | 2.3 | 221 | 66.9 | 40.9 | 263 |
| 80 | Primary tumor | FF | 500 | 93.8 | 2.3 | 243 | 60 | 39.2 | 646 |
| 81 | Primary tumor | FF | 500 | 101.8 | 2.3 | 227 | 59.4 | 40.2 | 295 |
| 82 | Primary tumor | FF | 500 | 219.9 | 2.2 | 237 | 59.1 | 39.5 | 415 |
| 83 | Primary tumor | FFPE | 200 | 38.6 | 2.4 | 136 | 39.6 | 46.7 | 609 |
| 84 | Primary tumor | FFPE | 500 | 69.2 | 2.3 | 182 | 41.5 | 43.6 | 371 |
| 85 | Primary tumor | FF | 500 | 107.6 | 2.3 | 219 | 59.2 | 40.6 | 397 |
| 86 | Primary tumor | FF | 200 | 116.2 | 2.2 | 192 | 60.2 | 41.6 | 630 |
| 87 | Primary tumor | FF | 500 | 120 | 2.2 | 222 | 52.7 | 40.8 | 477 |
| 88 | Primary tumor | FF | 500 | 192.4 | 2.2 | 233 | 57.5 | 39.7 | 571 |
| 89 | Primary tumor | FF | 500 | 91.7 | 2.3 | 235 | 61.6 | 39.9 | 611 |
| 90 (S1)a | Primary tumor | FFPE | 500 | 94.6 | 2.2 | 187 | 26.8 | 43.4 | 408 |
| 90 (S2)a | Primary tumor | FFPE | 500 | 70.8 | 2.1 | 171 | 68.8 | 44.4 | 480 |
| 91 | Primary tumor | FF | 500 | 82.8 | 2.3 | 225 | 64.1 | 40.4 | 489 |
| 92 | Primary tumor | FF | 500 | 132.1 | 2.1 | 226 | 61.5 | 40.5 | 6444 |
| 93 | Primary tumor | FFPE | 500 | 118.4 | 2.2 | 209 | 45.4 | 41.7 | 479 |
| 95 | Primary tumor | FF | 100 | 148.6 | 2.2 | 223 | 60.8 | 40.5 | 597 |
| 96 | Primary tumor | FFPE | 500 | 48.3 | 2.4 | 158 | 64.6 | 45.3 | 426 |
| 97 | Primary tumor | FFPE | 500 | 73.2 | 2.3 | 157 | 25.1 | 45.4 | 298 |
| 98 | Primary tumor | FFPE | 500 | 96.8 | 2.2 | 196 | 26.6 | 42.4 | 4659 |
| 99 | Primary tumor | FFPE | 500 | 125.1 | 2.2 | 226 | 44.6 | 40.5 | 503 |
| 100 | Primary tumor | FF | 500 | 81.5 | 2.3 | 199 | 64.7 | 41.8 | 467 |
| 101 | Primary tumor | FF | 500 | 88.1 | 2.3 | 190 | 64.5 | 42.3 | 359 |
| 102 | Primary tumor | FFPE | 500 | 127.7 | 2.3 | 167 | 44.8 | 45 | 432 |

© 2019 Reinert T et al. *JAMA Oncology.*

| eTable 4. Summary of Samples and WES Information. | | | | | | | | | |
|---|---|---|---|---|---|---|---|---|---|
| Patient ID | Source of DNA | Sample type[b] | Input, ng | WES coverage | Ti/Tv ratio | Median insert size (bp) | Percentage bases on target | Capture fold enrichment | # of variants |
| 103 | Primary tumor | FF | 500 | 169.3 | 2.2 | 227 | 57.8 | 40 | 596 |
| 104 | Primary tumor | FF | 500 | 99.9 | 2.2 | 242 | 52 | 39.6 | 247 |
| 105 | Primary tumor | FF | 100 | 383.4 | 2.2 | 222 | 60.6 | 40.1 | 884 |
| 106 | Primary tumor | FF | 500 | 83.2 | 2.2 | 217 | 64 | 40.8 | 4515 |
| 107 | Primary tumor | FFPE | 500 | 99.5 | 2.3 | 183 | 46.1 | 43.4 | 412 |
| 108 | Primary tumor | FF | 500 | 98.5 | 2.3 | 224 | 63.1 | 40.4 | 686 |
| 109 | Primary tumor | FF | 500 | 168.7 | 2.2 | 236 | 57.6 | 39.2 | 285 |
| 110 | Primary tumor | FF | 500 | 85.8 | 2.3 | 210 | 64.1 | 41.3 | 364 |
| 111 | Primary tumor | FFPE | 500 | 54.5 | 2.3 | 135 | 29.8 | 47.1 | 1019 |
| 112 | Primary tumor | FF | 500 | 86.5 | 2.3 | 216 | 67.7 | 40.9 | 315 |
| 113 | Primary tumor | FFPE | 500 | 92.2 | 2.3 | 133 | 32 | 47.6 | 604 |
| 114 (S1)a | Primary tumor | FF | 500 | 80.7 | 2.3 | 243 | 60.3 | 39.2 | 432 |
| 114 (S2)a | Primary tumor | FF | 500 | 98.6 | 2.3 | 248 | 59 | 38.7 | 603 |
| 115 | Primary tumor | FF | 500 | 179.6 | 2.2 | 244 | 57.8 | 39.1 | 683 |
| 116 | Primary tumor | FF | 500 | 97.6 | 2.3 | 239 | 57.8 | 39.5 | 436 |
| 117 | Primary tumor | FF | 500 | 91.5 | 2.2 | 228 | 54.8 | 40.7 | 602 |
| 118 | Primary tumor | FF | 500 | 94.4 | 2.2 | 218 | 66.9 | 41 | 6636 |
| 119 | Primary tumor | FFPE | 500 | 66 | 2.3 | 144 | 41 | 46.6 | 642 |
| 120 | Primary tumor | FF | 500 | 80.4 | 2.2 | 204 | 65.4 | 41.7 | 5666 |
| 121 | Primary tumor | FFPE | 500 | 94.1 | 2.3 | 137 | 35.1 | 47.7 | 697 |
| 122 | Primary tumor | FFPE | 500 | 61.2 | 2.3 | 143 | 44.6 | 46.4 | 457 |
| 123 | Primary tumor | FF | 500 | 176.9 | 2.2 | 236 | 58.1 | 39.3 | 525 |
| 124 | Primary tumor | FF | 250 | 137.2 | 2.2 | 220 | 59.5 | 40.5 | 642 |
| 125 | Primary tumor | FF | 500 | 119.6 | 2 | 214 | 61.7 | 41.1 | 492 |
| 126 | Primary tumor | FF | 500 | 102.6 | 2.2 | 225 | 54 | 40.7 | 345 |
| 127 | Primary tumor | FFPE | 500 | 83.1 | 2.2 | 136 | 32.9 | 47 | 7599 |
| 128 | Primary tumor | FFPE | 500 | 72.3 | 2.3 | 155 | 46.7 | 45.6 | 5622 |
| 130 | Primary tumor | FF | 500 | 88.8 | 2.3 | 217 | 65.2 | 40.9 | 452 |
| 1 | Buffy coat | NA | 500 | 41.9 | 2.1 | 247 | 64.7 | 37.5 | NA[c] |
| 2 | Buffy coat | NA | 500 | 36.5 | 2.3 | 244 | 64.9 | 37.6 | NA |
| 3 | Buffy coat | NA | 500 | 45.3 | 2.3 | 232 | 67.5 | 39.9 | NA |
| 4 | Buffy coat | NA | 500 | 45.5 | 2.3 | 240 | 66.7 | 39.2 | NA |
| 5 | Buffy coat | NA | 500 | 40.9 | 2.3 | 231 | 69.1 | 39.7 | NA |
| 6 | Buffy coat | NA | 500 | 46.1 | 2.3 | 238 | 67.2 | 39.4 | NA |
| 7 | Buffy coat | NA | 500 | 40.8 | 2.3 | 243 | 66.3 | 38.2 | NA |

© 2019 Reinert T et al. *JAMA Oncology.*

| eTable 4. Summary of Samples and WES Information. | | | | | | | | | |
|---|---|---|---|---|---|---|---|---|---|
| Patient ID | Source of DNA | Sample type[b] | Input, ng | WES coverage | Ti/Tv ratio | Median insert size (bp) | Percentage bases on target | Capture fold enrichment | # of variants |
| 8 | Buffy coat | NA | 500 | 43.2 | 2.3 | 223 | 68.6 | 40.5 | NA |
| 9 | Buffy coat | NA | 500 | 36.1 | 2.3 | 248 | 65.2 | 37.5 | NA |
| 10 | Buffy coat | NA | 500 | 41.8 | 2.3 | 230 | 69.6 | 39.9 | NA |
| 11 | Buffy coat | NA | 500 | 39.4 | 2.3 | 249 | 66.5 | 38.6 | NA |
| 12 | Buffy coat | NA | 500 | 40.8 | 2.3 | 246 | 65.1 | 37.7 | NA |
| 13 | Buffy coat | NA | 500 | 97 | 2.2 | 229 | 52.7 | 40.2 | NA |
| 14 | Buffy coat | NA | 500 | 61.3 | 2.3 | 248 | 64.9 | 37.6 | NA |
| 15 | Buffy coat | NA | 500 | 39.2 | 2.3 | 238 | 67.1 | 39.3 | NA |
| 16 | Buffy coat | NA | 500 | 92.2 | 2.3 | 267 | 51.5 | 36.3 | NA |
| 18 | Buffy coat | NA | 500 | 49.4 | 2.3 | 235 | 66.9 | 39.7 | NA |
| 19 | Buffy coat | NA | 500 | 37 | 2.3 | 243 | 52.8 | 37.6 | NA |
| 20 | Buffy coat | NA | 500 | 44.7 | 2.3 | 244 | 64.9 | 38.6 | NA |
| 20 | Buffy coat | NA | 500 | 44.7 | 2.3 | 244 | 64.9 | 38.6 | NA |
| 21 | Buffy coat | NA | 500 | 56.5 | 2.3 | 237 | 65.9 | 39.1 | NA |
| 22 | Buffy coat | NA | 500 | 54.9 | 2.3 | 234 | 66.2 | 39.4 | NA |
| 23 | Buffy coat | NA | 500 | 43.1 | 2.3 | 236 | 67.4 | 39.6 | NA |
| 24 | Buffy coat | NA | 500 | 104.2 | 2.3 | 220 | 66.6 | 40.8 | NA |
| 24 | Buffy coat | NA | 500 | 104.2 | 2.3 | 220 | 66.6 | 40.8 | NA |
| 25 | Buffy coat | NA | 500 | 43.6 | 2.3 | 253 | 52.7 | 37 | NA |
| 26 | Buffy coat | NA | 500 | 46.5 | 2.3 | 239 | 66.6 | 39.4 | NA |
| 27 | Buffy coat | NA | 500 | 54.9 | 2.3 | 241 | 65.7 | 38.9 | NA |
| 28 | Buffy coat | NA | 500 | 60.9 | 2.3 | 233 | 66 | 39.5 | NA |
| 29 | Buffy coat | NA | 500 | 45.1 | 2.2 | 234 | 67.3 | 39.7 | NA |
| 30 | Buffy coat | NA | 500 | 40 | 2.3 | 235 | 69.1 | 39.5 | NA |
| 31 | Buffy coat | NA | 500 | 56.8 | 2.1 | 233 | 66.1 | 39.4 | NA |
| 33 | Buffy coat | NA | 500 | 55.9 | 2.3 | 238 | 65.7 | 39.1 | NA |
| 34 | Buffy coat | NA | 500 | 46.8 | 2.3 | 247 | 52.9 | 37.3 | NA |
| 35 | Buffy coat | NA | 500 | 41.6 | 2.3 | 235 | 68.8 | 39.4 | NA |
| 36 | Buffy coat | NA | 500 | 36.8 | 2.3 | 235 | 69 | 39.4 | NA |
| 37 | Buffy coat | NA | 500 | 50.8 | 2.3 | 256 | 51.9 | 36.7 | NA |
| 38 | Buffy coat | NA | 500 | 54.2 | 2.3 | 252 | 52.6 | 37 | NA |
| 39 | Buffy coat | NA | 500 | 49.8 | 2.3 | 238 | 68.5 | 39.3 | NA |
| 40 | Buffy coat | NA | 500 | 62.7 | 2.3 | 280 | 62.1 | 36.4 | NA |
| 41 | Buffy coat | NA | 500 | 60.5 | 2.3 | 246 | 64.5 | 38.4 | NA |
| 42 | Buffy coat | NA | 500 | 35.9 | 2.3 | 245 | 65.1 | 37.6 | NA |

© 2019 Reinert T et al. *JAMA Oncology.*

**eTable 4. Summary of Samples and WES Information.**

| Patient ID | Source of DNA | Sample type[b] | Input, ng | WES coverage | Ti/Tv ratio | Median insert size (bp) | Percentage bases on target | Capture fold enrichment | # of variants |
|---|---|---|---|---|---|---|---|---|---|
| 43 | Buffy coat | NA | 500 | 35.2 | 2.3 | 242 | 65 | 37.7 | NA |
| 44 | Buffy coat | NA | 500 | 45.2 | 2.3 | 238 | 67.2 | 39.5 | NA |
| 45 | Buffy coat | NA | 500 | 44.8 | 2.3 | 239 | 67.1 | 39.4 | NA |
| 46 | Buffy coat | NA | 500 | 40.6 | 2.3 | 232 | 67.8 | 39.8 | NA |
| 47 | Buffy coat | NA | 500 | 37.6 | 2.3 | 241 | 65.3 | 37.8 | NA |
| 48 | Buffy coat | NA | 500 | 52.7 | 2.3 | 228 | 67.9 | 40.1 | NA |
| 49 | Buffy coat | NA | 500 | 57.7 | 2.3 | 233 | 68.9 | 39.7 | NA |
| 50 | Buffy coat | NA | 500 | 43.2 | 2.3 | 240 | 52.9 | 37.7 | NA |
| 51 | Buffy coat | NA | 500 | 36.4 | 2.3 | 243 | 52.5 | 37.4 | NA |
| 52 | Buffy coat | NA | 500 | 55.4 | 2.3 | 244 | 64.5 | 38.5 | NA |
| 53 | Buffy coat | NA | 500 | 56 | 2.3 | 234 | 65.8 | 39.3 | NA |
| 54 | Buffy coat | NA | 500 | 79.7 | 2.3 | 239 | 65 | 38.8 | NA |
| 55 | Buffy coat | NA | 500 | 69.1 | 2.3 | 234 | 68.7 | 39.6 | NA |
| 57 | Buffy coat | NA | 500 | 72.8 | 2.3 | 250 | 64.1 | 38.2 | NA |
| 58 | Buffy coat | NA | 500 | 53.7 | 2.3 | 237 | 64.9 | 38.9 | NA |
| 59 | Buffy coat | NA | 500 | 58.4 | 2.3 | 240 | 64.7 | 38.9 | NA |
| 60 | Buffy coat | NA | 500 | 57.4 | 2.3 | 233 | 65.4 | 39.3 | NA |
| 61 | Buffy coat | NA | 500 | 81.9 | 2.2 | 236 | 64.8 | 39 | NA |
| 62 | Buffy coat | NA | 500 | 40 | 2.3 | 235 | 68.7 | 39.4 | NA |
| 63 | Buffy coat | NA | 500 | 44.8 | 2.1 | 240 | 67.1 | 39.2 | NA |
| 64 | Buffy coat | NA | 500 | 41.3 | 2.3 | 243 | 66.8 | 39.1 | NA |
| 65 | Buffy coat | NA | 500 | 39.1 | 2.3 | 233 | 67.5 | 39.7 | NA |
| 66 | Buffy coat | NA | 500 | 47.7 | 2.3 | 231 | 67.4 | 39.9 | NA |
| 67 | Buffy coat | NA | 500 | 50.2 | 2.3 | 233 | 67.2 | 39.8 | NA |
| 68 | Buffy coat | NA | 500 | 42 | 2.3 | 232 | 68.1 | 39.9 | NA |
| 69 | Buffy coat | NA | 500 | 44.9 | 2.3 | 239 | 67.2 | 39.4 | NA |
| 70 | Buffy coat | NA | 500 | 39.8 | 2.4 | 234 | 68.6 | 39.5 | NA |
| 71 | Buffy coat | NA | 500 | 39.2 | 2.3 | 232 | 69 | 39.7 | NA |
| 72 | Buffy coat | NA | 500 | 57 | 2.3 | 232 | 66.1 | 39.5 | NA |
| 73 | Buffy coat | NA | 500 | 55.3 | 2.1 | 237 | 66.1 | 39.3 | NA |
| 74 | Buffy coat | NA | 500 | 44.7 | 2.3 | 234 | 67.3 | 39.7 | NA |
| 75 | Buffy coat | NA | 500 | 36.5 | 2.3 | 239 | 59.5 | 38.3 | NA |
| 76 | Buffy coat | NA | 500 | 55.6 | 2.3 | 242 | 65.4 | 38.9 | NA |
| 77 | Buffy coat | NA | 500 | 42.1 | 2.3 | 232 | 67.3 | 40 | NA |
| 77 | Buffy coat | NA | 500 | 42.1 | 2.3 | 232 | 67.3 | 40 | NA |

© 2019 Reinert T et al. *JAMA Oncology.*

| eTable 4. Summary of Samples and WES Information. | | | | | | | | | |
|---|---|---|---|---|---|---|---|---|---|
| **Patient ID** | **Source of DNA** | **Sample type[b]** | **Input, ng** | **WES coverage** | **Ti/Tv ratio** | **Median insert size (bp)** | **Percentage bases on target** | **Capture fold enrichment** | **# of variants** |
| 78 | Buffy coat | NA | 500 | 53.5 | 2.3 | 236 | 66.2 | 39.1 | NA |
| 79 | Buffy coat | NA | 500 | 54.1 | 2.3 | 243 | 65.6 | 38.6 | NA |
| 80 | Buffy coat | NA | 500 | 44.8 | 2.3 | 237 | 59.9 | 38.6 | NA |
| 81 | Buffy coat | NA | 500 | 38.3 | 2.3 | 248 | 65.5 | 37.8 | NA |
| 82 | Buffy coat | NA | 500 | 69.3 | 2.3 | 234 | 68.8 | 39.6 | NA |
| 83 | Buffy coat | NA | 500 | 52.2 | 2.3 | 231 | 66.5 | 39.3 | NA |
| 84 | Buffy coat | NA | 500 | 39.6 | 2.3 | 244 | 58.8 | 37.9 | NA |
| 85 | Buffy coat | NA | 500 | 63.3 | 2.3 | 247 | 65.6 | 37.9 | NA |
| 86 | Buffy coat | NA | 500 | 38.3 | 2.3 | 240 | 59 | 38.1 | NA |
| 87 | Buffy coat | NA | 500 | 39.7 | 2.3 | 241 | 59.7 | 38.3 | NA |
| 88 | Buffy coat | NA | 500 | 45.4 | 2.3 | 235 | 68.8 | 39.4 | NA |
| 89 | Buffy coat | NA | 500 | 56.5 | 2.3 | 234 | 66.5 | 39.1 | NA |
| 90 | Buffy coat | NA | 500 | 41.3 | 2.3 | 234 | 59.9 | 38.8 | NA |
| 91 | Buffy coat | NA | 500 | 84 | 2.2 | 240 | 65.7 | 38.7 | NA |
| 92 | Buffy coat | NA | 500 | 54.1 | 2.3 | 235 | 66.1 | 39.1 | NA |
| 93 | Buffy coat | NA | 500 | 41.1 | 2.3 | 233 | 60.1 | 38.8 | NA |
| 95 | Buffy coat | NA | 500 | 52.3 | 2.1 | 234 | 66.2 | 39.1 | NA |
| 96 | Buffy coat | NA | 500 | 52.9 | 2.3 | 235 | 65.9 | 39 | NA |
| 97 | Buffy coat | NA | 500 | 33.1 | 2.3 | 230 | 60.4 | 38.8 | NA |
| 98 | Buffy coat | NA | 500 | 41.6 | 2.3 | 243 | 59.4 | 38.2 | NA |
| 99 | Buffy coat | NA | 500 | 38.4 | 2.3 | 245 | 65.7 | 37.9 | NA |
| 100 | Buffy coat | NA | 500 | 38.4 | 2.3 | 246 | 65.7 | 38 | NA |
| 101 | Buffy coat | NA | 500 | 43.3 | 2.3 | 247 | 65.1 | 37.6 | NA |
| 102 | Buffy coat | NA | 500 | 32.9 | 2.2 | 238 | 65.4 | 37.9 | NA |
| 103 | Buffy coat | NA | 500 | 53.5 | 2.3 | 233 | 68.8 | 39.6 | NA |
| 104 | Buffy coat | NA | 500 | 107.4 | 2.2 | 236 | 64 | 39.1 | NA |
| 105 | Buffy coat | NA | 500 | 80.5 | 2.2 | 250 | 52.2 | 37 | NA |
| 106 | Buffy coat | NA | 500 | 46.9 | 2.3 | 258 | 52.2 | 36.9 | NA |
| 107 | Buffy coat | NA | 500 | 45.6 | 2.3 | 243 | 65.7 | 38 | NA |
| 108 | Buffy coat | NA | 500 | 57.3 | 2.3 | 241 | 65.8 | 38.2 | NA |
| 109 | Buffy coat | NA | 500 | 56.5 | 2.3 | 234 | 68.7 | 39.5 | NA |
| 110 | Buffy coat | NA | 500 | 35.6 | 2.3 | 241 | 65 | 37.8 | NA |
| 111 | Buffy coat | NA | 500 | 38.8 | 2.3 | 235 | 60.4 | 38.8 | NA |
| 112 | Buffy coat | NA | 500 | 54.3 | 2.3 | 235 | 66.2 | 39.3 | NA |
| 113 | Buffy coat | NA | 500 | 78.7 | 2.3 | 237 | 64.9 | 38.9 | NA |

© 2019 Reinert T et al. *JAMA Oncology.*

**eTable 4. Summary of Samples and WES Information.**

| Patient ID | Source of DNA | Sample type[b] | Input, ng | WES coverage | Ti/Tv ratio | Median insert size (bp) | Percentage bases on target | Capture fold enrichment | # of variants |
|---|---|---|---|---|---|---|---|---|---|
| 114 | Buffy coat | NA | 500 | 100.3 | 2.2 | 234 | 64.3 | 39.2 | NA |
| 115 | Buffy coat | NA | 500 | 38.2 | 2.3 | 236 | 68.7 | 39.4 | NA |
| 116 | Buffy coat | NA | 500 | 43.2 | 2.3 | 252 | 65.5 | 37.6 | NA |
| 117 | Buffy coat | NA | 500 | 74.2 | 2.3 | 242 | 65.6 | 38.6 | NA |
| 118 | Buffy coat | NA | 500 | 53 | 2.3 | 237 | 65.8 | 38.9 | NA |
| 119 | Buffy coat | NA | 500 | 54.9 | 2.3 | 239 | 65.8 | 38.8 | NA |
| 120 | Buffy coat | NA | 500 | 83 | 2.3 | 247 | 65.9 | 38.2 | NA |
| 121 | Buffy coat | NA | 500 | 127 | 2.2 | 225 | 65.9 | 40 | NA |
| 122 | Buffy coat | NA | 500 | 37.3 | 2.3 | 240 | 66.3 | 38.3 | NA |
| 123 | Buffy coat | NA | 500 | 56.5 | 2.3 | 234 | 68.7 | 39.5 | NA |
| 124 | Buffy coat | NA | 500 | 93.2 | 2.3 | 238 | 64.8 | 39.1 | NA |
| 125 | Buffy coat | NA | 500 | 35 | 2.3 | 236 | 59.9 | 38.5 | NA |
| 126 | Buffy coat | NA | 500 | 48.1 | 2.3 | 239 | 65.5 | 38.7 | NA |
| 127 | Buffy coat | NA | 500 | 48.5 | 2.3 | 239 | 66.2 | 38.5 | NA |
| 128 | Buffy coat | NA | 500 | 105.8 | 2.3 | 234 | 64.5 | 39.2 | NA |
| 130 | Buffy coat | NA | 500 | 50.4 | 2.3 | 248 | 52.9 | 37.2 | NA |

[a]Synchronous CRC were marked with S1 or S2
[b]FF=fresh frozen, FFPE=formalin fixed paraffin embedded,
[c]NA=Not applicable

© 2019 Reinert T et al. *JAMA Oncology.*

## eTable 5. ctDNA Results for All 795 Plasma Samples

| Patient ID | Recurrence | Time post surgery (months)[a] | # ctDNA positive targets | Input DNA (ng) | VAF (mean) | VAF CI 95% | Plasma (mL) | ctDNA copies per mL plasma |
|---|---|---|---|---|---|---|---|---|
| 1 | 0 | 1.2 | 0 | 24.83 | 0 | NA[b] | 8 | 0 |
| 1 | 0 | 3.3 | 0 | 8.65 | 0 | NA | 8.6 | 0 |
| 1 | 0 | 5.8 | 0 | 35.18 | 0 | NA | 8.8 | 0 |
| 1 | 0 | 11.5 | 0 | 47.61 | 0 | NA | 9 | 0 |
| 1 | 0 | 15.9 | 0 | 7.57 | 0 | NA | 7 | 0 |
| 1 | 0 | 18.9 | 0 | 31.62 | 0 | NA | 9 | 0 |
| 1 | 0 | 21.7 | 0 | 57.27 | 0 | NA | 9.1 | 0 |
| 1 | 0 | 24.6 | 0 | 16.54 | 0 | NA | 8.7 | 0 |
| 1 | 0 | 28.0 | 0 | 33.61 | 0 | NA | 9.2 | 0 |
| 1 | 0 | 30.8 | 0 | 45.2 | 0 | NA | 8.1 | 0 |
| 1 | 0 | 35.0 | 0 | 21.18 | 0 | NA | 7.5 | 0 |
| 2 | 0 | -0.1 | 12 | 5.56 | 0.00206 | (0.001-0.0031) | 7.6 | 0.52 |
| 2 | 0 | 1.2 | 0 | 28.88 | 0 | NA | 7.8 | 0 |
| 2 | 0 | 2.8 | 0 | 17.26 | 0 | NA | 7.7 | 0 |
| 2 | 0 | 5.8 | 0 | 21.39 | 0 | NA | 8.5 | 0 |
| 2 | 0 | 8.8 | 0 | 29.39 | 0 | NA | 8.1 | 0 |
| 2 | 0 | 11.3 | 0 | 46.8 | 0 | NA | 8.5 | 0 |
| 2 | 0 | 15.4 | 0 | 29.16 | 0 | NA | 9.3 | 0 |
| 2 | 0 | 18.7 | 0 | 27.85 | 0 | NA | 9.1 | 0 |
| 2 | 0 | 21.4 | 0 | 37.65 | 0 | NA | 8.5 | 0 |
| 2 | 0 | 24.5 | 0 | 28.8 | 0 | NA | 9 | 0 |
| 2 | 0 | 27.6 | 0 | 40.32 | 0 | NA | 8.8 | 0 |
| 2 | 0 | 30.6 | 0 | 47.2 | 0 | NA | 8 | 0 |
| 2 | 0 | 33.3 | 0 | 22.53 | 0 | NA | 9 | 0 |
| 2 | 0 | 35.2 | 0 | 31.45 | 0 | NA | 7.9 | 0 |
| 3 | 0 | 0.0 | 14 | 31.16 | 0.00217 | (0.0014-0.0029) | 4 | 5.74 |
| 3 | 0 | 1.4 | 0 | 66.0 | 0 | NA | 7.8 | 0 |
| 3 | 0 | 4.1 | 0 | 66.0 | 0 | NA | 8.2 | 0 |
| 3 | 0 | 6.2 | 0 | 66.0 | 0 | NA | 8 | 0 |

© 2019 Reinert T et al. *JAMA Oncology.*

## eTable 5. ctDNA Results for All 795 Plasma Samples

| Patient ID | Recurrence | Time post surgery (months)[a] | # ctDNA positive targets | Input DNA (ng) | VAF (mean) | VAF CI 95% | Plasma (mL) | ctDNA copies per mL plasma |
|---|---|---|---|---|---|---|---|---|
| 3 | 0 | 9.5 | 0 | 66.0 | 0 | NA | 7.5 | 0 |
| 3 | 0 | 15.7 | 0 | 66.0 | 0 | NA | 8.6 | 0 |
| 3 | 0 | 18.1 | 0 | 50.34 | 0 | NA | 8.3 | 0 |
| 3 | 0 | 21.2 | 0 | 48.96 | 0 | NA | 8.9 | 0 |
| 3 | 0 | 24.1 | 0 | 66.0 | 0 | NA | 9.2 | 0 |
| 3 | 0 | 28.0 | 0 | 41.22 | 0 | NA | 7.7 | 0 |
| 3 | 0 | 30.9 | 0 | 37.6 | 0 | NA | 8 | 0 |
| 3 | 0 | 34.5 | 0 | 34.3 | 0 | NA | 8.5 | 0 |
| 3 | 0 | 37.8 | 0 | 50.41 | 0 | NA | 8.5 | 0 |
| 4 | 0 | 0.0 | 10 | 3.02 | 0.00170 | (0.0011-0.0023) | 2 | 0.93 |
| 4 | 0 | 1.2 | 0 | 45.18 | 0 | NA | 8 | 0 |
| 4 | 0 | 3.1 | 0 | 66.0 | 0 | NA | 8.3 | 0 |
| 4 | 0 | 5.8 | 0 | 66.0 | 0 | NA | 7.9 | 0 |
| 4 | 0 | 8.8 | 0 | 66.0 | 0 | NA | 8 | 0 |
| 4 | 0 | 11.9 | 0 | 62.18 | 0 | NA | 8 | 0 |
| 4 | 0 | 14.9 | 0 | 63.06 | 0 | NA | 8.5 | 0 |
| 4 | 0 | 17.7 | 0 | 60.1 | 0 | NA | 9 | 0 |
| 4 | 0 | 20.9 | 0 | 66.0 | 0 | NA | 8.9 | 0 |
| 4 | 0 | 24.5 | 0 | 56.46 | 0 | NA | 8.7 | 0 |
| 4 | 0 | 26.9 | 0 | 53.75 | 0 | NA | 8.7 | 0 |
| 4 | 0 | 30.1 | 0 | 54.0 | 0 | NA | 8.3 | 0 |
| 4 | 0 | 32.7 | 0 | 31.52 | 0 | NA | 8.7 | 0 |
| 4 | 0 | 35.6 | 0 | 37.34 | 0 | NA | 8 | 0 |
| 5 | 0 | -0.1 | 16 | 66.0 | 0.01055 | (0.0075-0.0136) | 7.5 | 65.30 |
| 5 | 0 | 1.4 | 0 | 45.52 | 0 | NA | 7.2 | 0 |
| 5 | 0 | 3.3 | 0 | 27.32 | 0 | NA | 7.5 | 0 |
| 5 | 0 | 6.1 | 0 | 22.22 | 0 | NA | 8 | 0 |
| 5 | 0 | 9.2 | 0 | 33.08 | 0 | NA | 8 | 0 |
| 5 | 0 | 12.1 | 0 | 39.36 | 0 | NA | 8 | 0 |
| 5 | 0 | 14.8 | 0 | 26.77 | 0 | NA | 8 | 0 |

© 2019 Reinert T et al. *JAMA Oncology.*

## eTable 5. ctDNA Results for All 795 Plasma Samples

| Patient ID | Recurrence | Time post surgery (months)[a] | # ctDNA positive targets | Input DNA (ng) | VAF (mean) | VAF CI 95% | Plasma (mL) | ctDNA copies per mL plasma |
|---|---|---|---|---|---|---|---|---|
| 5 | 0 | 17.7 | 0 | 26.32 | 0 | NA | 9.3 | 0 |
| 5 | 0 | 20.5 | 0 | 33.84 | 0 | NA | 8.5 | 0 |
| 5 | 0 | 23.8 | 0 | 33.26 | 0 | NA | 8 | 0 |
| 6 | 0 | -0.1 | 15 | 63.09 | 0.00164 | (0.0013-0.002) | 8.5 | 4.14 |
| 6 | 0 | 1.9 | 0 | 54.04 | 0 | NA | 8 | 0 |
| 6 | 0 | 3.3 | 0 | 46.39 | 0 | NA | 8 | 0 |
| 6 | 0 | 6.0 | 0 | 53.2 | 0 | NA | 8 | 0 |
| 6 | 0 | 9.2 | 0 | 32.63 | 0 | NA | 9 | 0 |
| 6 | 0 | 11.4 | 0 | 36.96 | 0 | NA | 8.6 | 0 |
| 6 | 0 | 14.6 | 0 | 66.0 | 0 | NA | 9.5 | 0 |
| 6 | 0 | 18.0 | 0 | 62.07 | 0 | NA | 9.3 | 0 |
| 6 | 0 | 21.4 | 0 | 41.71 | 0 | NA | 9.2 | 0 |
| 6 | 0 | 24.2 | 0 | 53.2 | 0 | NA | 9.2 | 0 |
| 6 | 0 | 27.5 | 0 | 29.13 | 0 | NA | 8 | 0 |
| 6 | 0 | 31.3 | 0 | 50.38 | 0 | NA | 8.5 | 0 |
| 6 | 0 | 34.6 | 0 | 47.53 | 0 | NA | 8.6 | 0 |
| 7 | 0 | -0.1 | 0 | 63.8 | 0 | NA | 8.2 | 0 |
| 7 | 0 | 1.2 | 0 | 51.85 | 0 | NA | 8.5 | 0 |
| 7 | 0 | 3.3 | 0 | 43.2 | 0 | NA | 9 | 0 |
| 7 | 0 | 6.2 | 0 | 22.92 | 0 | NA | 9.5 | 0 |
| 7 | 0 | 9.0 | 0 | 45.66 | 0 | NA | 8.5 | 0 |
| 7 | 0 | 12.0 | 0 | 51.24 | 0 | NA | 9.5 | 0 |
| 7 | 0 | 14.9 | 0 | 41.52 | 0 | NA | 8.8 | 0 |
| 7 | 0 | 17.9 | 0 | 35.2 | 0 | NA | 8.6 | 0 |
| 7 | 0 | 21.0 | 0 | 36.0 | 0 | NA | 8.2 | 0 |
| 7 | 0 | 23.9 | 0 | 33.02 | 0 | NA | 8.9 | 0 |
| 7 | 0 | 27.2 | 0 | 59.72 | 0 | NA | 8.1 | 0 |
| 7 | 0 | 30.2 | 0 | 29.19 | 0 | NA | 9 | 0 |
| 7 | 0 | 33.1 | 0 | 33.45 | 0 | NA | 9 | 0 |
| 8 | 0 | 0.9 | 0 | 1.22 | 0 | NA | 8 | 0 |

© 2019 Reinert T et al. *JAMA Oncology.*

## eTable 5. ctDNA Results for All 795 Plasma Samples

| Patient ID | Recurrence | Time post surgery (months)[a] | # ctDNA positive targets | Input DNA (ng) | VAF (mean) | VAF CI 95% | Plasma (mL) | ctDNA copies per mL plasma |
|---|---|---|---|---|---|---|---|---|
| 8 | 0 | 3.0 | 0 | 66.0 | 0 | NA | 8.5 | 0 |
| 8 | 0 | 4.4 | 0 | 39.88 | 0 | NA | 9 | 0 |
| 8 | 0 | 7.6 | 0 | 29.39 | 0 | NA | 8.5 | 0 |
| 8 | 0 | 12.0 | 0 | 26.0 | 0 | NA | 9 | 0 |
| 8 | 0 | 14.7 | 0 | 34.54 | 0 | NA | 9 | 0 |
| 8 | 0 | 17.7 | 0 | 16.0 | 0 | NA | 9 | 0 |
| 8 | 0 | 20.8 | 0 | 19.16 | 0 | NA | 8.5 | 0 |
| 8 | 0 | 23.8 | 0 | 29.95 | 0 | NA | 7.5 | 0 |
| 8 | 0 | 27.1 | 0 | 34.8 | 0 | NA | 8 | 0 |
| 8 | 0 | 30.2 | 0 | 12.85 | 0 | NA | 8 | 0 |
| 8 | 0 | 33.4 | 0 | 43.6 | 0 | NA | 8.5 | 0 |
| 9 | 0 | 0.0 | 14 | 30.2 | 0.01623 | (0.0106-0.0219) | 8.5 | 19.83 |
| 9 | 0 | 0.9 | 0 | 66.0 | 0 | NA | 9 | 0 |
| 9 | 0 | 2.6 | 0 | 66.0 | 0 | NA | 9 | 0 |
| 9 | 0 | 5.2 | 0 | 66.0 | 0 | NA | 9 | 0 |
| 9 | 0 | 7.9 | 0 | 38.99 | 0 | NA | 8.5 | 0 |
| 9 | 0 | 11.5 | 0 | 66.0 | 0 | NA | 9.2 | 0 |
| 9 | 0 | 15.8 | 0 | 59.32 | 0 | NA | 10 | 0 |
| 9 | 0 | 19.2 | 0 | 42.8 | 0 | NA | 9 | 0 |
| 9 | 0 | 23.3 | 0 | 33.0 | 0 | NA | 8.5 | 0 |
| 9 | 0 | 26.9 | 0 | 47.5 | 0 | NA | 9 | 0 |
| 9 | 0 | 29.2 | 0 | 66.0 | 0 | NA | 8.5 | 0 |
| 9 | 0 | 32.7 | 0 | 66.0 | 0 | NA | 9 | 0 |
| 10 | 0 | 0.0 | 0 | 13.32 | 0 | NA | 8 | 0 |
| 10 | 0 | 2.8 | 0 | 66.0 | 0 | NA | 10 | 0 |
| 10 | 0 | 6.7 | 0 | 66.0 | 0 | NA | 9 | 0 |
| 10 | 0 | 10.2 | 0 | 66.0 | 0 | NA | 9.7 | 0 |
| 10 | 0 | 12.7 | 0 | 66.0 | 0 | NA | 8.7 | 0 |
| 10 | 0 | 16.2 | 0 | 66.0 | 0 | NA | 8.8 | 0 |
| 10 | 0 | 18.7 | 0 | 66.0 | 0 | NA | 8.9 | 0 |

© 2019 Reinert T et al. *JAMA Oncology.*

## eTable 5. ctDNA Results for All 795 Plasma Samples

| Patient ID | Recurrence | Time post surgery (months)[a] | # ctDNA positive targets | Input DNA (ng) | VAF (mean) | VAF CI 95% | Plasma (mL) | ctDNA copies per mL plasma |
|---|---|---|---|---|---|---|---|---|
| 10 | 0 | 21.9 | 0 | 66.0 | 0 | NA | 9.3 | 0 |
| 10 | 0 | 24.7 | 0 | 63.6 | 0 | NA | 9 | 0 |
| 10 | 0 | 28.1 | 0 | 66.0 | 0 | NA | 9.4 | 0 |
| 11 | 0 | -0.1 | 0 | 14.17 | 0 | NA | 8 | 0 |
| 11 | 0 | 1.4 | 0 | 21.98 | 0 | NA | 8 | 0 |
| 11 | 0 | 6.1 | 0 | 66.0 | 0 | NA | 9.3 | 0 |
| 11 | 0 | 8.6 | 0 | 66.0 | 0 | NA | 9.3 | 0 |
| 11 | 0 | 12.0 | 0 | 66.0 | 0 | NA | 8.5 | 0 |
| 11 | 0 | 14.8 | 0 | 66.0 | 0 | NA | 9.2 | 0 |
| 11 | 0 | 18.2 | 0 | 66.0 | 0 | NA | 8.9 | 0 |
| 11 | 0 | 21.2 | 0 | 66.0 | 0 | NA | 8.7 | 0 |
| 11 | 0 | 24.2 | 0 | 66.0 | 0 | NA | 8.5 | 0 |
| 11 | 0 | 26.9 | 0 | 66.0 | 0 | NA | 8.5 | 0 |
| 11 | 0 | 30.5 | 0 | 66.0 | 0 | NA | 9.1 | 0 |
| 12 | 0 | 0.0 | 5 | 42.63 | 0.00033 | (0.0002-0.0004) | 8.3 | 0.53 |
| 12 | 0 | 1.5 | 0 | 28.45 | 0 | NA | 9 | 0 |
| 12 | 0 | 3.1 | 0 | 35.5 | 0 | NA | 9.3 | 0 |
| 12 | 0 | 6.1 | 0 | 26.39 | 0 | NA | 8.3 | 0 |
| 12 | 0 | 9.3 | 0 | 23.67 | 0 | NA | 8.7 | 0 |
| 12 | 0 | 12.1 | 0 | 21.54 | 0 | NA | 9.2 | 0 |
| 13 | 0 | 0.0 | 9 | 33.8 | 0.00036 | (0.0002-0.0005) | 8.8 | 0.47 |
| 13 | 0 | 1.9 | 0 | 29.68 | 0 | NA | 9 | 0 |
| 13 | 0 | 5.8 | 0 | 64.89 | 0 | NA | 8.6 | 0 |
| 13 | 0 | 8.8 | 0 | 56.76 | 0 | NA | 8.2 | 0 |
| 13 | 0 | 12.7 | 0 | 66.0 | 0 | NA | 8.2 | 0 |
| 13 | 0 | 14.8 | 0 | 28.94 | 0 | NA | 9.5 | 0 |
| 13 | 0 | 18.0 | 0 | 42.6 | 0 | NA | 9 | 0 |
| 13 | 0 | 21.4 | 0 | 66.0 | 0 | NA | 8.6 | 0 |
| 13 | 0 | 24.4 | 0 | 48.69 | 0 | NA | 8.3 | 0 |
| 13 | 0 | 27.3 | 0 | 52.46 | 0 | NA | 8.7 | 0 |

© 2019 Reinert T et al. *JAMA Oncology.*

## eTable 5. ctDNA Results for All 795 Plasma Samples

| Patient ID | Recurrence | Time post surgery (months)[a] | # ctDNA positive targets | Input DNA (ng) | VAF (mean) | VAF CI 95% | Plasma (mL) | ctDNA copies per mL plasma |
|---|---|---|---|---|---|---|---|---|
| 13 | 0 | 30.2 | 0 | 35.6 | 0 | NA | 8.7 | 0 |
| 14 | 0 | 0.0 | 14 | 30.07 | 0.00132 | (0.0009-0.0017) | 8 | 1.7 |
| 14 | 0 | 1.1 | 0 | 38.96 | 0 | NA | 8.6 | 0 |
| 14 | 0 | 3.6 | 0 | 31.47 | 0 | NA | 8.6 | 0 |
| 14 | 0 | 6.0 | 0 | 28.58 | 0 | NA | 9 | 0 |
| 14 | 0 | 8.9 | 0 | 25.51 | 0 | NA | 8.5 | 0 |
| 14 | 0 | 11.5 | 0 | 27.84 | 0 | NA | 7.3 | 0 |
| 14 | 0 | 15.0 | 0 | 41.57 | 0 | NA | 8.5 | 0 |
| 14 | 0 | 22.1 | 0 | 46.73 | 0 | NA | 8 | 0 |
| 14 | 0 | 25.3 | 0 | 57.21 | 0 | NA | 8.4 | 0 |
| 15 | 0 | 0.0 | 2 | 1.61 | 0.00761 | (0.0015-0.0121) | 8 | 0.47 |
| 15 | 0 | 1.5 | 0 | 3.62 | 0 | NA | 8 | 0 |
| 15 | 0 | 3.6 | 0 | 37.94 | 0 | NA | 8.4 | 0 |
| 15 | 0 | 5.8 | 0 | 25.76 | 0 | NA | 8 | 0 |
| 15 | 0 | 8.9 | 0 | 32.14 | 0 | NA | 8.9 | 0 |
| 15 | 0 | 12.0 | 0 | 21.03 | 0 | NA | 9 | 0 |
| 15 | 0 | 15.2 | 0 | 41.67 | 0 | NA | 8.7 | 0 |
| 15 | 0 | 18.7 | 0 | 43.74 | 0 | NA | 9 | 0 |
| 15 | 0 | 25.4 | 0 | 37.41 | 0 | NA | 8.6 | 0 |
| 15 | 0 | 29.7 | 0 | 35.74 | 0 | NA | 8.5 | 0 |
| 16 | 0 | 0.0 | 10 | 32.47 | 0.00042 | (0.0003-0.0005) | 9 | 0.46 |
| 16 | 0 | 2.1 | 0 | 31.13 | 0 | NA | 9 | 0 |
| 16 | 0 | 5.7 | 0 | 31.97 | 0 | NA | 9.4 | 0 |
| 16 | 0 | 8.5 | 0 | 36.34 | 0 | NA | 8.6 | 0 |
| 16 | 0 | 11.7 | 0 | 47.03 | 0 | NA | 8.6 | 0 |
| 16 | 0 | 15.4 | 0 | 37.2 | 0 | NA | 9 | 0 |
| 16 | 0 | 18.9 | 0 | 37.07 | 0 | NA | 7.5 | 0 |
| 16 | 0 | 21.8 | 0 | 28.0 | 0 | NA | 8.5 | 0 |
| 16 | 0 | 24.8 | 0 | 36.24 | 0 | NA | 8.2 | 0 |
| 16 | 0 | 27.8 | 0 | 43.67 | 0 | NA | 7.5 | 0 |

© 2019 Reinert T et al. *JAMA Oncology.*

## eTable 5. ctDNA Results for All 795 Plasma Samples

| Patient ID | Recurrence | Time post surgery (months)[a] | # ctDNA positive targets | Input DNA (ng) | VAF (mean) | VAF CI 95% | Plasma (mL) | ctDNA copies per mL plasma |
|---|---|---|---|---|---|---|---|---|
| 16 | 0 | 30.8 | 0 | 42.21 | 0 | NA | 8.5 | 0 |
| 18 | 1 | 0.0 | 16 | 44.0 | 0.00259 | (0.0013-0.0039) | 8 | 4.9 |
| 18 | 1 | 1.4 | 0 | 36.15 | 0 | NA | 8 | 0 |
| 18 | 1 | 3.4 | 0 | 66.0 | 0 | NA | 9 | 0 |
| 18 | 1 | 7.3 | 8 | 66.0 | 0.00034 | (0.0002-0.0005) | 8 | 1.4 |
| 18 | 1 | 10.6 | 5 | 66.0 | 0.00037 | (0.0001-0.0006) | 9 | 1.4 |
| 18 | 1 | 13.7 | 0 | 66.0 | 0 | NA | 8.5 | 0 |
| 18 | 1 | 16.4 | 0 | 66.0 | 0 | NA | 9 | 0 |
| 18 | 1 | 19.6 | 0 | 65.73 | 0 | NA | 9 | 0 |
| 18 | 1 | 21.9 | 0 | 59.13 | 0 | NA | 8.5 | 0 |
| 18 | 1 | 27.4 | 0 | 66.0 | 0 | NA | 8.5 | 0 |
| 18 | 1 | 31.1 | 0 | 66.0 | 0 | NA | 8.5 | 0 |
| 19 | 0 | 0.0 | 14 | 66.0 | 0.00197 | (0.0014-0.0026) | 8.8 | 42 |
| 19 | 0 | 1.0 | 0 | 66.0 | 0 | NA | 8 | 0 |
| 19 | 0 | 2.7 | 0 | 66.0 | 0 | NA | 8.5 | 0 |
| 19 | 0 | 6.6 | 0 | 59.7 | 0 | NA | 9.5 | 0 |
| 19 | 0 | 8.8 | 0 | 66.0 | 0 | NA | 9 | 0 |
| 19 | 0 | 11.9 | 0 | 31.27 | 0 | NA | 7.5 | 0 |
| 19 | 0 | 15.4 | 0 | 66.0 | 0 | NA | 9.4 | 0 |
| 19 | 0 | 18.2 | 0 | 66.0 | 0 | NA | 8.1 | 0 |
| 19 | 0 | 21.4 | 0 | 66.0 | 0 | NA | 8.2 | 0 |
| 19 | 0 | 24.1 | 0 | 51.56 | 0 | NA | 8.3 | 0 |
| 19 | 0 | 26.8 | 0 | 58.0 | 0 | NA | 8.6 | 0 |
| 20 | 1 | 0.0 | 5 | 26.8 | 0.00026 | (0.0002-0.0003) | 8 | 0.29 |
| 20 | 1 | 0.8 | 0 | 21.31 | 0 | NA | 8 | 0 |
| 20 | 1 | 2.7 | 0 | 30.45 | 0 | NA | 8.4 | 0 |
| 20 | 1 | 6.4 | 0 | 36.27 | 0 | NA | 9.5 | 0 |
| 20 | 1 | 8.7 | 0 | 26.81 | 0 | NA | 8.7 | 0 |
| 20 | 1 | 11.7 | 0 | 28.52 | 0 | NA | 9 | 0 |
| 20 | 1 | 15.1 | 0 | 39.12 | 0 | NA | 8.3 | 0 |

© 2019 Reinert T et al. *JAMA Oncology.*

## eTable 5. ctDNA Results for All 795 Plasma Samples

| Patient ID | Recurrence | Time post surgery (months)[a] | # ctDNA positive targets | Input DNA (ng) | VAF (mean) | VAF CI 95% | Plasma (mL) | ctDNA copies per mL plasma |
|---|---|---|---|---|---|---|---|---|
| 20 | 1 | 18.5 | 0 | 32.92 | 0 | NA | 8.5 | 0 |
| 20 | 1 | 21.1 | 0 | 66.0 | 0 | NA | 9 | 0 |
| 20 | 1 | 26.7 | 0 | 45.2 | 0 | NA | 9 | 0 |
| 21 | 0 | 0.0 | 13 | 17.12 | 0.00133 | (0.0008-0.0019) | 8 | 0.97 |
| 21 | 0 | 2.7 | 0 | 36.3 | 0 | NA | 8.8 | 0 |
| 21 | 0 | 6.1 | 0 | 60.19 | 0 | NA | 9.3 | 0 |
| 21 | 0 | 11.5 | 0 | 25.8 | 0 | NA | 8.2 | 0 |
| 21 | 0 | 15.6 | 0 | 37.12 | 0 | NA | 9.5 | 0 |
| 21 | 0 | 19.7 | 0 | 44.55 | 0 | NA | 9 | 0 |
| 21 | 0 | 24.0 | 0 | 33.05 | 0 | NA | 8.8 | 0 |
| 21 | 0 | 26.8 | 0 | 35.25 | 0 | NA | 8.8 | 0 |
| 22 | 0 | 0.0 | 2 | 1.51 | 0.00138 | (0.0013-0.0015) | 8 | 0.09 |
| 22 | 0 | 1.3 | 0 | 18.81 | 0 | NA | 8 | 0 |
| 22 | 0 | 2.2 | 0 | 14.65 | 0 | NA | 8 | 0 |
| 22 | 0 | 4.8 | 0 | 45.27 | 0 | NA | 7.8 | 0 |
| 22 | 0 | 8.2 | 0 | 59.92 | 0 | NA | 8.7 | 0 |
| 22 | 0 | 11.9 | 0 | 31.34 | 0 | NA | 9.4 | 0 |
| 22 | 0 | 14.4 | 0 | 29.39 | 0 | NA | 9.5 | 0 |
| 22 | 0 | 17.9 | 0 | 27.59 | 0 | NA | 8.9 | 0 |
| 22 | 0 | 20.6 | 0 | 31.2 | 0 | NA | 8.2 | 0 |
| 22 | 0 | 23.7 | 0 | 14.82 | 0 | NA | 8.4 | 0 |
| 22 | 0 | 26.7 | 0 | 22.68 | 0 | NA | 8.5 | 0 |
| 23 | 0 | 0.0 | 6 | 8.84 | 0.00141 | (0.0005-0.0023) | 8 | 0.53 |
| 23 | 0 | 1.1 | 0 | 7.93 | 0 | NA | 8 | 0 |
| 23 | 0 | 2.6 | 0 | 66.0 | 0 | NA | 9.2 | 0 |
| 23 | 0 | 5.8 | 0 | 53.91 | 0 | NA | 8.7 | 0 |
| 23 | 0 | 8.4 | 0 | 29.68 | 0 | NA | 8.9 | 0 |
| 23 | 0 | 11.4 | 0 | 28.8 | 0 | NA | 9.2 | 0 |
| 23 | 0 | 14.4 | 0 | 22.1 | 0 | NA | 9.1 | 0 |
| 23 | 0 | 17.2 | 0 | 43.42 | 0 | NA | 8.5 | 0 |

© 2019 Reinert T et al. *JAMA Oncology.*

## eTable 5. ctDNA Results for All 795 Plasma Samples

| Patient ID | Recurrence | Time post surgery (months)[a] | # ctDNA positive targets | Input DNA (ng) | VAF (mean) | VAF CI 95% | Plasma (mL) | ctDNA copies per mL plasma |
|---|---|---|---|---|---|---|---|---|
| 23 | 0 | 20.1 | 0 | 22.22 | 0 | NA | 8.5 | 0 |
| 23 | 0 | 23.4 | 0 | 27.81 | 0 | NA | 8.4 | 0 |
| 23 | 0 | 26.6 | 0 | 39.14 | 0 | NA | 8 | 0 |
| 24 | 1 | 0.0 | 11 | 12.4 | 0.00088 | (0.0006-0.0012) | 8 | 0.47 |
| 24 | 1 | 1.0 | 0 | 5.96 | 0 | NA | 8 | 0 |
| 24 | 1 | 3.1 | 0 | 31.4 | 0 | NA | 9 | 0 |
| 24 | 1 | 5.7 | 0 | 26.97 | 0 | NA | 9.3 | 0 |
| 24 | 1 | 8.9 | 0 | 22.3 | 0 | NA | 9 | 0 |
| 24 | 1 | 12.3 | 0 | 22.44 | 0 | NA | 8.6 | 0 |
| 24 | 1 | 14.8 | 0 | 26.4 | 0 | NA | 9.2 | 0 |
| 24 | 1 | 17.8 | 0 | 15.81 | 0 | NA | 8.5 | 0 |
| 24 | 1 | 21.7 | 0 | 19.12 | 0 | NA | 8.3 | 0 |
| 24 | 1 | 25.8 | 0 | 25.91 | 0 | NA | 9 | 0 |
| 25 | 0 | 0.0 | 16 | 45.73 | 0.00657 | (0.0044-0.0088) | 8 | 13 |
| 25 | 0 | 1.2 | 0 | 57.58 | 0 | NA | 8.5 | 0 |
| 25 | 0 | 3.1 | 0 | 30.05 | 0 | NA | 9.5 | 0 |
| 25 | 0 | 6.1 | 0 | 30.67 | 0 | NA | 9.5 | 0 |
| 25 | 0 | 8.8 | 0 | 35.19 | 0 | NA | 9.6 | 0 |
| 25 | 0 | 10.5 | 0 | 38.06 | 0 | NA | 9 | 0 |
| 25 | 0 | 14.8 | 0 | 50.93 | 0 | NA | 9 | 0 |
| 25 | 0 | 17.8 | 0 | 29.92 | 0 | NA | 9 | 0 |
| 25 | 0 | 21.2 | 0 | 50.66 | 0 | NA | 8.8 | 0 |
| 25 | 0 | 24.8 | 0 | 42.39 | 0 | NA | 8.4 | 0 |
| 25 | 0 | 28.1 | 0 | 37.63 | 0 | NA | 8.7 | 0 |
| 26 | 0 | 0.0 | 16 | 66.0 | 0.00943 | (0.0072-0.0117) | 8 | 33 |
| 26 | 0 | 1.2 | 0 | 25.77 | 0 | NA | 8 | 0 |
| 26 | 0 | 3.6 | 0 | 48.66 | 0 | NA | 8.9 | 0 |
| 26 | 0 | 6.0 | 0 | 45.85 | 0 | NA | 9 | 0 |
| 26 | 0 | 9.0 | 0 | 66.0 | 0 | NA | 8.7 | 0 |
| 26 | 0 | 12.4 | 0 | 60.83 | 0 | NA | 8.4 | 0 |

© 2019 Reinert T et al. *JAMA Oncology.*

## eTable 5. ctDNA Results for All 795 Plasma Samples

| Patient ID | Recurrence | Time post surgery (months)[a] | # ctDNA positive targets | Input DNA (ng) | VAF (mean) | VAF CI 95% | Plasma (mL) | ctDNA copies per mL plasma |
|---|---|---|---|---|---|---|---|---|
| 26 | 0 | 14.8 | 0 | 66.0 | 0 | NA | 9.5 | 0 |
| 26 | 0 | 18.2 | 0 | 66.0 | 0 | NA | 9 | 0 |
| 26 | 0 | 21.4 | 0 | 66.0 | 0 | NA | 8 | 0 |
| 26 | 0 | 24.1 | 0 | 66.0 | 0 | NA | 9 | 0 |
| 27 | 0 | -0.1 | 14 | 18.4 | 0.00369 | (0.002-0.0049) | 8 | 2.7 |
| 27 | 0 | 1.0 | 0 | 21.71 | 0 | NA | 8 | 0 |
| 27 | 0 | 3.1 | 0 | 36.36 | 0 | NA | 9.2 | 0 |
| 27 | 0 | 6.0 | 0 | 26.0 | 0 | NA | 8.9 | 0 |
| 27 | 0 | 9.9 | 0 | 23.27 | 0 | NA | 9.5 | 0 |
| 27 | 0 | 12.0 | 0 | 23.02 | 0 | NA | 9.4 | 0 |
| 27 | 0 | 15.0 | 0 | 28.11 | 0 | NA | 8 | 0 |
| 27 | 0 | 18.0 | 0 | 29.88 | 0 | NA | 8.7 | 0 |
| 27 | 0 | 22.1 | 0 | 20.92 | 0 | NA | 8 | 0 |
| 27 | 0 | 25.3 | 0 | 30.0 | 0 | NA | 8.6 | 0 |
| 28 | 1 | 0.0 | 7 | 7.25 | 0.00069 | (0.0005-0.001) | 8 | 0.22 |
| 28 | 1 | 0.9 | 0 | 10.73 | 0 | NA | 8 | 0 |
| 28 | 1 | 2.8 | 0 | 66.0 | 0 | NA | 9.6 | 0 |
| 28 | 1 | 6.2 | 0 | 66.0 | 0 | NA | 8.9 | 0 |
| 28 | 1 | 9.7 | 0 | 66.0 | 0 | NA | 8.5 | 0 |
| 28 | 1 | 12.3 | 0 | 49.6 | 0 | NA | 8.3 | 0 |
| 28 | 1 | 15.2 | 13 | 22.1 | 0.00066 | (0.0004-0.001) | 8.5 | 0.62 |
| 28 | 1 | 18.0 | 16 | 44.93 | 0.00182 | (0.0013-0.0024) | 9.1 | 3.1 |
| 28 | 1 | 21.8 | 16 | 52.92 | 0.00777 | (0.006-0.0095) | 8.5 | 16 |
| 28 | 1 | 25.3 | 16 | 66.0 | 0.01076 | (0.0086-0.0129) | 8.3 | 43 |
| 29 | 1 | 0.0 | 0 | 6.74 | 0 | NA | 8 | 0 |
| 29 | 1 | 1.9 | 0 | 12.91 | 0 | NA | 8 | 0 |
| 29 | 1 | 3.4 | 0 | 39.36 | 0 | NA | 8.5 | 0 |
| 29 | 1 | 6.3 | 0 | 26.46 | 0 | NA | 9.3 | 0 |
| 29 | 1 | 10.9 | 11 | 33.2 | 0.00067 | (0.0004-0.0009) | 8.8 | 0.85 |
| 29 | 1 | 15.5 | 6 | 22.29 | 0.00041 | (0.0002-0.0006) | 8.4 | 0.38 |

© 2019 Reinert T et al. *JAMA Oncology.*

## eTable 5. ctDNA Results for All 795 Plasma Samples

| Patient ID | Recurrence | Time post surgery (months)[a] | # ctDNA positive targets | Input DNA (ng) | VAF (mean) | VAF CI 95% | Plasma (mL) | ctDNA copies per mL plasma |
|---|---|---|---|---|---|---|---|---|
| 29 | 1 | 17.9 | 13 | 25.51 | 0.00095 | (0.0007-0.0012) | 8.3 | 1.0 |
| 29 | 1 | 22.1 | 4 | 33.52 | 0.00042 | (0.0002-0.0006) | 3.3 | 1.4 |
| 30 | 1 | 0.0 | 15 | 14.11 | 0.04070 | (0.0315-0.05) | 8 | 24 |
| 30 | 1 | 1.2 | 15 | 14.15 | 0.07946 | (0.0645-0.0947) | 8 | 47 |
| 30 | 1 | 3.0 | 5 | 66.0 | 0.00013 | (0.0001-0.0002) | 8.5 | 3.2 |
| 30 | 1 | 6.0 | 0 | 66.0 | 0 | NA | 9 | 0 |
| 30 | 1 | 9.4 | 14 | 66.0 | 0.00343 | (0.0026-0.0043) | 9 | 15 |
| 30 | 1 | 13.5 | 15 | 66.0 | 0.33035 | (0.2524-0.4076) | 9.3 | 2743 |
| 30 | 1 | 15.6 | 15 | 66.0 | 0.00118 | (0.0009-0.0015) | 9 | 4.5 |
| 30 | 1 | 18.6 | 15 | 66.0 | 0.04747 | (0.0343-0.0608) | 8.7 | 181 |
| 31 | 0 | 0.0 | 4 | 24.63 | 0.00035 | (0.0003-0.0004) | 8 | 0.36 |
| 31 | 0 | 1.1 | 0 | 19.17 | 0 | NA | 8 | 0 |
| 31 | 0 | 3.2 | 0 | 66.0 | 0 | NA | 9.2 | 0 |
| 31 | 0 | 6.0 | 0 | 66.0 | 0 | NA | 8.3 | 0 |
| 31 | 0 | 9.3 | 0 | 66.0 | 0 | NA | 9.1 | 0 |
| 31 | 0 | 12.1 | 0 | 66.0 | 0 | NA | 8.8 | 0 |
| 31 | 0 | 15.1 | 0 | 66.0 | 0 | NA | 9.1 | 0 |
| 31 | 0 | 18.2 | 0 | 66.0 | 0 | NA | 8.3 | 0 |
| 31 | 0 | 21.3 | 0 | 49.92 | 0 | NA | 8.2 | 0 |
| 31 | 0 | 25.0 | 0 | 43.5 | 0 | NA | 8.2 | 0 |
| 33 | 0 | 0.0 | 9 | 12.73 | 0.00992 | (0.0025-0.0174) | 6 | 7.2 |
| 33 | 0 | 1.1 | 7 | 16.8 | 0.00718 | (0.0024-0.01) | 8 | 4.4 |
| 33 | 0 | 2.8 | 0 | 58.2 | 0 | NA | 8 | 0 |
| 33 | 0 | 5.6 | 0 | 54.16 | 0 | NA | 9 | 0 |
| 33 | 0 | 8.7 | 0 | 39.02 | 0 | NA | 9 | 0 |
| 33 | 0 | 9.9 | 0 | 51.56 | 0 | NA | 8 | 0 |
| 33 | 0 | 11.7 | 0 | 47.92 | 0 | NA | 9.5 | 0 |
| 33 | 0 | 15.3 | 0 | 40.8 | 0 | NA | 8.4 | 0 |
| 33 | 0 | 18.3 | 0 | 32.01 | 0 | NA | 8.5 | 0 |
| 33 | 0 | 21.9 | 0 | 29.17 | 0 | NA | 8.7 | 0 |

© 2019 Reinert T et al. *JAMA Oncology.*

## eTable 5. ctDNA Results for All 795 Plasma Samples

| Patient ID | Recurrence | Time post surgery (months)[a] | # ctDNA positive targets | Input DNA (ng) | VAF (mean) | VAF CI 95% | Plasma (mL) | ctDNA copies per mL plasma |
|---|---|---|---|---|---|---|---|---|
| 34 | 1 | 0.0 | 12 | 34.4 | 0.00049 | (0.0004-0.0006) | 9.3 | 0.62 |
| 34 | 1 | 1.2 | 0 | 31.75 | 0 | NA | 9.2 | 0 |
| 34 | 1 | 2.6 | 0 | 41.44 | 0 | NA | 8.5 | 0 |
| 34 | 1 | 5.3 | 0 | 34.79 | 0 | NA | 8.5 | 0 |
| 34 | 1 | 8.5 | 0 | 35.36 | 0 | NA | 9 | 0 |
| 34 | 1 | 11.2 | 0 | 66.0 | 0 | NA | 8.9 | 0 |
| 34 | 1 | 14.0 | 7 | 29.6 | 0.00051 | (0.0004-0.0007) | 8.4 | 0.61 |
| 34 | 1 | 17.1 | 14 | 31.76 | 0.00185 | (0.0015-0.0022) | 8.5 | 2.4 |
| 34 | 1 | 20.5 | 14 | 42.63 | 0.08891 | (0.0768-0.1009) | 8.5 | 152 |
| 35 | 0 | 0.0 | 12 | 66.0 | 0.00042 | (0.0003-0.0005) | 9.4 | 1.5 |
| 35 | 0 | 0.9 | 0 | 57.65 | 0 | NA | 8.1 | 0 |
| 35 | 0 | 2.0 | 0 | 37.32 | 0 | NA | 8.7 | 0 |
| 35 | 0 | 6.1 | 0 | 66.0 | 0 | NA | 9 | 0 |
| 35 | 0 | 9.0 | 0 | 45.58 | 0 | NA | 9 | 0 |
| 35 | 0 | 11.9 | 0 | 40.29 | 0 | NA | 8.5 | 0 |
| 35 | 0 | 15.1 | 0 | 55.17 | 0 | NA | 8.8 | 0 |
| 35 | 0 | 18.3 | 0 | 56.49 | 0 | NA | 9 | 0 |
| 36 | 0 | 0.0 | 15 | 62.06 | 0.00098 | (0.0006-0.0013) | 8.9 | 2.3 |
| 36 | 0 | 1.0 | 0 | 66.0 | 0 | NA | 9.5 | 0 |
| 36 | 0 | 3.0 | 0 | 66.0 | 0 | NA | 9.5 | 0 |
| 36 | 0 | 6.3 | 0 | 66.0 | 0 | NA | 9.2 | 0 |
| 36 | 0 | 8.9 | 0 | 65.38 | 0 | NA | 9 | 0 |
| 36 | 0 | 11.8 | 0 | 51.1 | 0 | NA | 8.7 | 0 |
| 36 | 0 | 14.7 | 0 | 41.29 | 0 | NA | 8.9 | 0 |
| 36 | 0 | 18.0 | 0 | 66.0 | 0 | NA | 9 | 0 |
| 37 | 1 | 0.0 | 16 | 49.2 | 0.00582 | (0.0043-0.0073) | 8.8 | 11 |
| 37 | 1 | 1.7 | 16 | 36.8 | 0.00440 | (0.0035-0.0053) | 9.5 | 5.8 |
| 37 | 1 | 3.0 | 16 | 14.44 | 0.01216 | (0.0091-0.0154) | 9.2 | 6.53 |
| 37 | 1 | 5.8 | 16 | 55.16 | 0.00609 | (0.0044-0.0078) | 8.4 | 14 |
| 37 | 1 | 9.0 | 0 | 32.66 | 0 | NA | 9 | 0 |

© 2019 Reinert T et al. *JAMA Oncology.*

**eTable 5. ctDNA Results for All 795 Plasma Samples**

| Patient ID | Recurrence | Time post surgery (months)[a] | # ctDNA positive targets | Input DNA (ng) | VAF (mean) | VAF CI 95% | Plasma (mL) | ctDNA copies per mL plasma |
|---|---|---|---|---|---|---|---|---|
| 37 | 1 | 12.2 | 0 | 29.99 | 0 | NA | 8.6 | 0 |
| 37 | 1 | 15.2 | 0 | 29.62 | 0 | NA | 8.5 | 0 |
| 37 | 1 | 18.5 | 0 | 31.6 | 0 | NA | 8.6 | 0 |
| 38 | 0 | 0.0 | 7 | 4.29 | 0.00147 | (0.0007-0.0022) | 8.8 | 0.25 |
| 38 | 0 | 1.0 | 0 | 30.25 | 0 | NA | 9 | 0 |
| 38 | 0 | 4.5 | 0 | 27.15 | 0 | NA | 8.5 | 0 |
| 38 | 0 | 6.5 | 0 | 36.41 | 0 | NA | 9 | 0 |
| 38 | 0 | 9.4 | 0 | 29.05 | 0 | NA | 8.9 | 0 |
| 38 | 0 | 12.1 | 0 | 20.8 | 0 | NA | 8.5 | 0 |
| 38 | 0 | 16.8 | 0 | 16.79 | 0 | NA | 8.3 | 0 |
| 39 | 0 | 0.0 | 16 | 40.4 | 0.00508 | (0.0041-0.0061) | 9.6 | 7.3 |
| 39 | 0 | 1.0 | 0 | 66.0 | 0 | NA | 8.9 | 0 |
| 39 | 0 | 3.9 | 0 | 49.73 | 0 | NA | 9.5 | 0 |
| 39 | 0 | 6.5 | 0 | 66.0 | 0 | NA | 9.1 | 0 |
| 39 | 0 | 9.7 | 0 | 49.02 | 0 | NA | 8.5 | 0 |
| 39 | 0 | 12.3 | 0 | 50.25 | 0 | NA | 9 | 0 |
| 39 | 0 | 15.9 | 0 | 39.6 | 0 | NA | 8.5 | 0 |
| 39 | 0 | 18.7 | 0 | 36.58 | 0 | NA | 7.9 | 0 |
| 40 | 0 | 0.0 | 10 | 41.6 | 0.00027 | (0.0002-0.0004) | 9.2 | 0.40 |
| 40 | 0 | 0.9 | 0 | 48.23 | 0 | NA | 9 | 0 |
| 40 | 0 | 3.3 | 0 | 66.0 | 0 | NA | 9.2 | 0 |
| 40 | 0 | 6.2 | 0 | 66.0 | 0 | NA | 9.2 | 0 |
| 40 | 0 | 9.4 | 0 | 66.0 | 0 | NA | 9 | 0 |
| 40 | 0 | 11.8 | 0 | 66.0 | 0 | NA | 9.3 | 0 |
| 41 | 0 | 0.0 | 16 | 35.1 | 0.00819 | (0.0061-0.0103) | 9 | 11 |
| 41 | 0 | 0.5 | 0 | 54.27 | 0 | NA | 9 | 0 |
| 41 | 0 | 3.0 | 0 | 54.62 | 0 | NA | 9 | 0 |
| 41 | 0 | 6.2 | 0 | 28.8 | 0 | NA | 8.5 | 0 |
| 41 | 0 | 11.8 | 0 | 28.23 | 0 | NA | 8.8 | 0 |
| 42 | 1 | 0.0 | 10 | 27.29 | 0.00060 | (0.0005-0.0007) | 7.3 | 0.76 |

© 2019 Reinert T et al. *JAMA Oncology.*

## eTable 5. ctDNA Results for All 795 Plasma Samples

| Patient ID | Recurrence | Time post surgery (months)[a] | # ctDNA positive targets | Input DNA (ng) | VAF (mean) | VAF CI 95% | Plasma (mL) | ctDNA copies per mL plasma |
|---|---|---|---|---|---|---|---|---|
| 42 | 1 | 2.5 | 0 | 66.0 | 0 | NA | 7.8 | 0 |
| 43 | 0 | -0.1 | 16 | 27.63 | 0.00226 | (0.0016-0.003) | 6.8 | 3.1 |
| 43 | 0 | 2.6 | 0 | 33.39 | 0 | NA | 7.5 | 0 |
| 44 | 0 | 0.0 | 2 | 39.8 | 0.00026 | NA | 4 | 0.78 |
| 44 | 0 | 1.8 | 0 | 37.67 | 0 | NA | 8 | 0 |
| 45 | 0 | 0.0 | 0 | 1.0 | 0 | NA | 8 | 0 |
| 45 | 0 | 2.3 | 0 | 44.8 | 0 | NA | 9 | 0 |
| 46 | 0 | -0.1 | 4 | 8.4 | 0.00182 | (0.0008-0.0025) | 4 | 1.2 |
| 46 | 0 | 1.7 | 0 | 36.56 | 0 | NA | 9.4 | 0 |
| 47 | 0 | 0.0 | 16 | 66.0 | 0.00815 | (0.0061-0.0102) | 9.1 | 23 |
| 47 | 0 | 3.4 | 0 | 45.97 | 0 | NA | 7.3 | 0 |
| 48 | 0 | 0.0 | 0 | 6.8 | 0 | NA | 8 | 0 |
| 48 | 0 | 0.4 | 0 | 4.64 | 0 | NA | 8 | 0 |
| 49 | 0 | 0.0 | 2 | 42.4 | 0.00019 | (0.0002-0.0002) | 8 | 0.33 |
| 49 | 0 | 0.4 | 0 | 66.0 | 0 | NA | 7.5 | 0 |
| 50 | 0 | 0.0 | 12 | 41.96 | 0.00150 | (0.0008-0.0022) | 8.8 | 2.4 |
| 50 | 0 | 0.5 | 0 | 66.0 | 0 | NA | 9.1 | 0 |
| 51 | 0 | 0.0 | 16 | 28.4 | 0.01376 | (0.0101-0.0175) | 8 | 17 |
| 51 | 0 | 0.5 | 0 | 66.0 | 0 | NA | 8.2 | 0 |
| 52 | 0 | -0.3 | 15 | 66.0 | 0.00112 | (0.0008-0.0014) | 8.5 | 4.7 |
| 52 | 0 | 0.6 | 0 | 45.2 | 0 | NA | 8 | 0 |
| 53 | 0 | 0.0 | 15 | 62.4 | 0.00561 | (0.0044-0.0069) | 9.2 | 13 |
| 53 | 0 | 0.2 | 0 | 66.0 | 0 | NA | 8.5 | 0 |
| 54 | 0 | 0.0 | 16 | 66.0 | 0.00155 | (0.0012-0.002) | 9.6 | 19 |
| 54 | 0 | 0.5 | 0 | 66.0 | 0 | NA | 8.8 | 0 |
| 55 | 0 | 0.0 | 5 | 65.26 | 0.00059 | (0.0001-0.0011) | 8.8 | 1.5 |
| 55 | 0 | 0.4 | 0 | 66.0 | 0 | NA | 9 | 0 |
| 57 | 0 | 0.0 | 7 | 46.2 | 0.00095 | (0.0004-0.0015) | 9 | 1.7 |
| 57 | 0 | 0.9 | 0 | 66.0 | 0 | NA | 9.5 | 0 |
| 58 | 0 | 0.0 | 8 | 61.6 | 0.00025 | (0.0002-0.0003) | 8.5 | 0.63 |

© 2019 Reinert T et al. *JAMA Oncology.*

## eTable 5. ctDNA Results for All 795 Plasma Samples

| Patient ID | Recurrence | Time post surgery (months)[a] | # ctDNA positive targets | Input DNA (ng) | VAF (mean) | VAF CI 95% | Plasma (mL) | ctDNA copies per mL plasma |
|---|---|---|---|---|---|---|---|---|
| 58 | 0 | 1.0 | 0 | 66.0 | 0 | NA | 8.2 | 0 |
| 59 | 0 | 0.0 | 0 | 10.8 | 0 | NA | 9 | 0 |
| 59 | 0 | 0.9 | 0 | 66.0 | 0 | NA | 9.3 | 0 |
| 60 | 0 | 0.0 | 12 | 50.8 | 0.00043 | (0.0003-0.0006) | 9.4 | 0.79 |
| 60 | 0 | 0.9 | 0 | 66.0 | 0 | NA | 9.4 | 0 |
| 61 | 0 | 0.0 | 10 | 33.46 | 0.00090 | (0.0006-0.0012) | 8.5 | 1.2 |
| 61 | 0 | 0.6 | 0 | 65.6 | 0 | NA | 8.8 | 0 |
| 62 | 0 | -0.3 | 8 | 2.37 | 0.00168 | (0.0005-0.0029) | 8 | 0.17 |
| 62 | 0 | 0.7 | 5 | 9.77 | 0.00063 | (0.0004-0.0008) | 8 | 0.26 |
| 62 | 0 | 2.8 | 0 | 66.0 | 0 | NA | 8.6 | 0 |
| 62 | 0 | 6.0 | 0 | 43.39 | 0 | NA | 8.5 | 0 |
| 62 | 0 | 9.0 | 0 | 28.95 | 0 | NA | 8.7 | 0 |
| 62 | 0 | 12.5 | 0 | 31.6 | 0 | NA | 7 | 0 |
| 62 | 0 | 15.1 | 0 | 21.27 | 0 | NA | 8.6 | 0 |
| 62 | 0 | 17.7 | 0 | 27.62 | 0 | NA | 8.2 | 0 |
| 62 | 0 | 21.1 | 0 | 41.97 | 0 | NA | 8.6 | 0 |
| 62 | 0 | 23.7 | 0 | 50.25 | 0 | NA | 9 | 0 |
| 62 | 0 | 26.7 | 0 | 31.62 | 0 | NA | 8.5 | 0 |
| 62 | 0 | 29.7 | 0 | 37.6 | 0 | NA | 8.5 | 0 |
| 63 | 0 | -0.2 | 12 | 13.09 | 0.00926 | (0.0052-0.0134) | 8 | 5.2 |
| 63 | 0 | 0.9 | 0 | 66.0 | 0 | NA | 8 | 0 |
| 63 | 0 | 3.5 | 0 | 55.57 | 0 | NA | 9 | 0 |
| 63 | 0 | 5.6 | 0 | 47.87 | 0 | NA | 9.5 | 0 |
| 63 | 0 | 9.2 | 0 | 43.09 | 0 | NA | 8.8 | 0 |
| 63 | 0 | 12.4 | 0 | 61.6 | 0 | NA | 8.7 | 0 |
| 63 | 0 | 15.0 | 0 | 31.29 | 0 | NA | 8.7 | 0 |
| 63 | 0 | 18.2 | 0 | 43.22 | 0 | NA | 8.2 | 0 |
| 63 | 0 | 21.0 | 0 | 51.11 | 0 | NA | 8.8 | 0 |
| 63 | 0 | 23.5 | 0 | 66.0 | 0 | NA | 8.5 | 0 |
| 63 | 0 | 26.6 | 0 | 45.19 | 0 | NA | 7.8 | 0 |

© 2019 Reinert T et al. *JAMA Oncology.*

## eTable 5. ctDNA Results for All 795 Plasma Samples

| Patient ID | Recurrence | Time post surgery (months)[a] | # ctDNA positive targets | Input DNA (ng) | VAF (mean) | VAF CI 95% | Plasma (mL) | ctDNA copies per mL plasma |
|---|---|---|---|---|---|---|---|---|
| 63 | 0 | 30.1 | 0 | 42.4 | 0 | NA | 8.2 | 0 |
| 64 | 0 | -0.2 | 3 | 2.94 | 0.00133 | (0.0009-0.0017) | 8 | 0.16 |
| 64 | 0 | 0.7 | 0 | 8.26 | 0 | NA | 8 | 0 |
| 64 | 0 | 3.1 | 0 | 66.0 | 0 | NA | 8.4 | 0 |
| 64 | 0 | 6.0 | 0 | 66.0 | 0 | NA | 9.5 | 0 |
| 64 | 0 | 9.6 | 0 | 43.65 | 0 | NA | 8.5 | 0 |
| 64 | 0 | 11.8 | 0 | 45.8 | 0 | NA | 8.5 | 0 |
| 64 | 0 | 15.5 | 0 | 28.37 | 0 | NA | 8.3 | 0 |
| 64 | 0 | 18.0 | 0 | 52.38 | 0 | NA | 8.7 | 0 |
| 64 | 0 | 21.3 | 0 | 59.28 | 0 | NA | 8 | 0 |
| 64 | 0 | 24.0 | 0 | 56.33 | 0 | NA | 8.4 | 0 |
| 64 | 0 | 26.6 | 0 | 41.2 | 0 | NA | 8 | 0 |
| 64 | 0 | 30.0 | 0 | 30.33 | 0 | NA | 8 | 0 |
| 65 | 0 | -0.3 | 8 | 10.4 | 0.00135 | (0.0007-0.002) | 8 | 0.60 |
| 65 | 0 | 0.7 | 0 | 12.86 | 0 | NA | 8 | 0 |
| 65 | 0 | 2.9 | 0 | 66.0 | 0 | NA | 9 | 0 |
| 65 | 0 | 6.0 | 0 | 66.0 | 0 | NA | 9 | 0 |
| 65 | 0 | 8.8 | 0 | 66.0 | 0 | NA | 9 | 0 |
| 65 | 0 | 11.6 | 0 | 41.77 | 0 | NA | 8.7 | 0 |
| 66 | 0 | -0.2 | 3 | 3.49 | 0.00318 | (0-0.008) | 8 | 0.54 |
| 66 | 0 | 0.6 | 0 | 14.88 | 0 | NA | 8 | 0 |
| 66 | 0 | 3.0 | 0 | 66.0 | 0 | NA | 8.8 | 0 |
| 66 | 0 | 6.0 | 0 | 43.8 | 0 | NA | 8.7 | 0 |
| 66 | 0 | 9.0 | 0 | 42.25 | 0 | NA | 9 | 0 |
| 66 | 0 | 11.8 | 0 | 27.01 | 0 | NA | 9.1 | 0 |
| 66 | 0 | 15.2 | 0 | 32.46 | 0 | NA | 8.5 | 0 |
| 66 | 0 | 18.2 | 0 | 38.0 | 0 | NA | 8 | 0 |
| 66 | 0 | 21.0 | 0 | 24.79 | 0 | NA | 8.5 | 0 |
| 66 | 0 | 24.0 | 0 | 27.48 | 0 | NA | 8.8 | 0 |
| 66 | 0 | 27.4 | 0 | 35.25 | 0 | NA | 8 | 0 |

© 2019 Reinert T et al. *JAMA Oncology.*

## eTable 5. ctDNA Results for All 795 Plasma Samples

| Patient ID | Recurrence | Time post surgery (months)[a] | # ctDNA positive targets | Input DNA (ng) | VAF (mean) | VAF CI 95% | Plasma (mL) | ctDNA copies per mL plasma |
|---|---|---|---|---|---|---|---|---|
| 66 | 0 | 30.2 | 0 | 51.16 | 0 | NA | 8.4 | 0 |
| 67 | 0 | -0.3 | 10 | 23.24 | 0.00064 | (0.0004-0.0008) | 8 | 0.63 |
| 67 | 0 | 0.9 | 0 | 23.2 | 0 | NA | 8 | 0 |
| 67 | 0 | 2.8 | 0 | 66.0 | 0 | NA | 8.6 | 0 |
| 67 | 0 | 5.7 | 0 | 66.0 | 0 | NA | 9 | 0 |
| 67 | 0 | 8.7 | 0 | 66.0 | 0 | NA | 8.5 | 0 |
| 67 | 0 | 11.5 | 0 | 55.68 | 0 | NA | 8.5 | 0 |
| 67 | 0 | 15.0 | 0 | 66.0 | 0 | NA | 8.3 | 0 |
| 67 | 0 | 18.0 | 0 | 66.0 | 0 | NA | 8 | 0 |
| 67 | 0 | 20.8 | 0 | 66.0 | 0 | NA | 8.5 | 0 |
| 67 | 0 | 24.0 | 0 | 66.0 | 0 | NA | 8.6 | 0 |
| 67 | 0 | 27.6 | 0 | 66.0 | 0 | NA | 8 | 0 |
| 67 | 0 | 30.0 | 0 | 66.0 | 0 | NA | 8.5 | 0 |
| 68 | 1 | -0.2 | 14 | 15.9 | 0.00142 | (0.0008-0.002) | 8 | 0.96 |
| 68 | 1 | 1.0 | 0 | 13.93 | 0 | NA | 8 | 0 |
| 68 | 1 | 3.1 | 6 | 19.33 | 0.00044 | (0.0003-0.0006) | 8.9 | 0.32 |
| 68 | 1 | 6.1 | 2 | 31.81 | 0.00030 | NA | 8.5 | 0.34 |
| 68 | 1 | 9.4 | 11 | 31.08 | 0.00075 | (0.0005-0.0009) | 9 | 0.86 |
| 68 | 1 | 12.0 | 16 | 40.71 | 0.00218 | (0.0017-0.0026) | 8.8 | 3.4 |
| 68 | 1 | 15.0 | 16 | 35.09 | 0.01345 | (0.0106-0.0164) | 8.8 | 18 |
| 68 | 1 | 18.4 | 16 | 25.56 | 0.00420 | (0.0029-0.0055) | 8.5 | 4.3 |
| 68 | 1 | 21.6 | 16 | 8.02 | 0.00516 | (0.0037-0.0065) | 8.5 | 1.7 |
| 69 | 0 | -0.2 | 8 | 12.35 | 0.00065 | (0.0004-0.0008) | 8 | 0.31 |
| 69 | 0 | 0.9 | 0 | 5.37 | 0 | NA | 8 | 0 |
| 69 | 0 | 3.0 | 0 | 66.0 | 0 | NA | 8.2 | 0 |
| 69 | 0 | 6.0 | 0 | 66.0 | 0 | NA | 8.7 | 0 |
| 69 | 0 | 8.9 | 0 | 40.17 | 0 | NA | 8.6 | 0 |
| 69 | 0 | 12.0 | 0 | 33.33 | 0 | NA | 8.5 | 0 |
| 69 | 0 | 15.2 | 0 | 34.23 | 0 | NA | 8.5 | 0 |
| 69 | 0 | 18.0 | 0 | 30.15 | 0 | NA | 8.3 | 0 |

© 2019 Reinert T et al. *JAMA Oncology.*

## eTable 5. ctDNA Results for All 795 Plasma Samples

| Patient ID | Recurrence | Time post surgery (months)[a] | # ctDNA positive targets | Input DNA (ng) | VAF (mean) | VAF CI 95% | Plasma (mL) | ctDNA copies per mL plasma |
|---|---|---|---|---|---|---|---|---|
| 69 | 0 | 21.0 | 0 | 50.25 | 0 | NA | 8.5 | 0 |
| 69 | 0 | 23.9 | 0 | 43.27 | 0 | NA | 8 | 0 |
| 69 | 0 | 27.1 | 0 | 50.93 | 0 | NA | 8 | 0 |
| 70 | 0 | -0.3 | 3 | 19.69 | 0.00048 | (0.0001-0.0009) | 8 | 0.41 |
| 70 | 0 | 0.9 | 0 | 36.35 | 0 | NA | 8 | 0 |
| 70 | 0 | 3.0 | 0 | 66.0 | 0 | NA | 8.5 | 0 |
| 70 | 0 | 6.1 | 0 | 66.0 | 0 | NA | 8.7 | 0 |
| 70 | 0 | 8.9 | 0 | 42.8 | 0 | NA | 8.5 | 0 |
| 70 | 0 | 12.0 | 0 | 26.12 | 0 | NA | 8.4 | 0 |
| 70 | 0 | 15.0 | 0 | 34.62 | 0 | NA | 8.5 | 0 |
| 70 | 0 | 18.1 | 0 | 37.67 | 0 | NA | 8.5 | 0 |
| 70 | 0 | 20.9 | 0 | 42.63 | 0 | NA | 8.4 | 0 |
| 71 | 0 | -0.2 | 2 | 1.65 | 0.01366 | (0-0.0294) | 8 | 0.78 |
| 71 | 0 | 0.7 | 0 | 12.28 | 0 | NA | 8 | 0 |
| 71 | 0 | 3.3 | 0 | 66.0 | 0 | NA | 8.6 | 0 |
| 71 | 0 | 6.2 | 0 | 66.0 | 0 | NA | 8.4 | 0 |
| 71 | 0 | 9.2 | 0 | 52.1 | 0 | NA | 9.5 | 0 |
| 71 | 0 | 12.1 | 0 | 63.2 | 0 | NA | 9 | 0 |
| 71 | 0 | 14.7 | 0 | 66.0 | 0 | NA | 8.8 | 0 |
| 71 | 0 | 18.4 | 0 | 48.83 | 0 | NA | 8 | 0 |
| 71 | 0 | 21.0 | 0 | 40.4 | 0 | NA | 8.5 | 0 |
| 71 | 0 | 23.9 | 0 | 47.73 | 0 | NA | 8 | 0 |
| 71 | 0 | 26.4 | 0 | 66.0 | 0 | NA | 8.3 | 0 |
| 72 | 0 | -0.4 | 16 | 26.13 | 0.00127 | (0.001-0.0015) | 9 | 1.2 |
| 72 | 0 | 0.8 | 0 | 1.64 | 0 | NA | 8 | 0 |
| 72 | 0 | 3.3 | 0 | 22.71 | 0 | NA | 9 | 0 |
| 72 | 0 | 6.0 | 0 | 35.07 | 0 | NA | 8.9 | 0 |
| 72 | 0 | 9.0 | 0 | 29.06 | 0 | NA | 9 | 0 |
| 72 | 0 | 12.1 | 0 | 23.14 | 0 | NA | 8.3 | 0 |
| 72 | 0 | 15.1 | 0 | 26.86 | 0 | NA | 8.9 | 0 |

© 2019 Reinert T et al. *JAMA Oncology.*

## eTable 5. ctDNA Results for All 795 Plasma Samples

| Patient ID | Recurrence | Time post surgery (months)[a] | # ctDNA positive targets | Input DNA (ng) | VAF (mean) | VAF CI 95% | Plasma (mL) | ctDNA copies per mL plasma |
|---|---|---|---|---|---|---|---|---|
| 72 | 0 | 18.0 | 0 | 28.95 | 0 | NA | 8.7 | 0 |
| 72 | 0 | 21.0 | 0 | 40.03 | 0 | NA | 8.5 | 0 |
| 72 | 0 | 24.0 | 0 | 30.8 | 0 | NA | 9 | 0 |
| 73 | 0 | -0.3 | 0 | 12.68 | 0 | NA | 8 | 0 |
| 73 | 0 | 0.8 | 0 | 15.2 | 0 | NA | 8 | 0 |
| 73 | 0 | 2.9 | 0 | 66.0 | 0 | NA | 9.4 | 0 |
| 73 | 0 | 5.9 | 0 | 66.0 | 0 | NA | 8.4 | 0 |
| 73 | 0 | 8.4 | 0 | 66.0 | 0 | NA | 8.8 | 0 |
| 73 | 0 | 12.1 | 0 | 66.0 | 0 | NA | 8.5 | 0 |
| 73 | 0 | 15.3 | 0 | 62.03 | 0 | NA | 8.6 | 0 |
| 73 | 0 | 18.1 | 0 | 66.0 | 0 | NA | 8 | 0 |
| 73 | 0 | 21.4 | 0 | 66.0 | 0 | NA | 8.5 | 0 |
| 73 | 0 | 24.1 | 0 | 61.87 | 0 | NA | 8.7 | 0 |
| 74 | 0 | -0.2 | 2 | 30.66 | 0.00053 | (0-0.0011) | 8 | 0.69 |
| 74 | 0 | 0.5 | 0 | 20.8 | 0 | NA | 8 | 0 |
| 74 | 0 | 3.7 | 0 | 60.49 | 0 | NA | 8.7 | 0 |
| 74 | 0 | 6.2 | 0 | 52.95 | 0 | NA | 8 | 0 |
| 74 | 0 | 9.5 | 0 | 63.08 | 0 | NA | 8.5 | 0 |
| 74 | 0 | 12.2 | 0 | 66.0 | 0 | NA | 8.3 | 0 |
| 74 | 0 | 14.8 | 0 | 59.77 | 0 | NA | 8.2 | 0 |
| 74 | 0 | 18.0 | 0 | 66.0 | 0 | NA | 8 | 0 |
| 74 | 0 | 21.4 | 0 | 56.84 | 0 | NA | 8.5 | 0 |
| 75 | 1 | -0.2 | 14 | 15.81 | 0.02285 | (0.008-0.0379) | 8 | 16 |
| 75 | 1 | 0.5 | 0 | 66.0 | 0 | NA | 8 | 0 |
| 75 | 1 | 3.2 | 15 | 26.0 | 0.10138 | (0.0395-0.1637) | 4 | 225 |
| 75 | 1 | 5.8 | 15 | 66.0 | 0.11723 | (0.0519-0.1829) | 8.7 | 311 |
| 75 | 1 | 8.6 | 15 | 66.0 | 0.20991 | (0.122-0.2986) | 8 | 857 |
| 75 | 1 | 11.7 | 15 | 66.0 | 0.42629 | (0.3125-0.5401) | 8 | 8240 |
| 75 | 1 | 14.7 | 0 | 34.88 | 0 | NA | 6.5 | 0 |
| 75 | 1 | 18.2 | 0 | 32.87 | 0 | NA | 8.2 | 0 |

© 2019 Reinert T et al. *JAMA Oncology.*

## eTable 5. ctDNA Results for All 795 Plasma Samples

| Patient ID | Recurrence | Time post surgery (months)[a] | # ctDNA positive targets | Input DNA (ng) | VAF (mean) | VAF CI 95% | Plasma (mL) | ctDNA copies per mL plasma |
|---|---|---|---|---|---|---|---|---|
| 75 | 1 | 20.3 | 2 | 27.53 | 0.00036 | (0.0003-0.0004) | 8 | 0.43 |
| 75 | 1 | 24.4 | 0 | 60.4 | 0 | NA | 8 | 0 |
| 76 | 0 | -0.2 | 0 | 1.0 | 0 | NA | 8 | 0 |
| 76 | 0 | 0.9 | 0 | 36.5 | 0 | NA | 8 | 0 |
| 76 | 0 | 3.0 | 0 | 66.0 | 0 | NA | 8 | 0 |
| 76 | 0 | 5.9 | 0 | 66.0 | 0 | NA | 8 | 0 |
| 76 | 0 | 8.2 | 0 | 66.0 | 0 | NA | 8.8 | 0 |
| 76 | 0 | 12.2 | 0 | 66.0 | 0 | NA | 9 | 0 |
| 76 | 0 | 14.9 | 0 | 66.0 | 0 | NA | 8.4 | 0 |
| 76 | 0 | 17.7 | 0 | 66.0 | 0 | NA | 8.5 | 0 |
| 76 | 0 | 20.5 | 0 | 66.0 | 0 | NA | 8.5 | 0 |
| 76 | 0 | 23.3 | 0 | 66.0 | 0 | NA | 8.4 | 0 |
| 77 | 1 | -0.2 | 2 | 26.27 | 0.00065 | (0.0003-0.001) | 8 | 0.73 |
| 77 | 1 | 0.7 | 0 | 56.18 | 0 | NA | 8 | 0 |
| 77 | 1 | 3.2 | 0 | 66.0 | 0 | NA | 8.6 | 0 |
| 77 | 1 | 5.7 | 0 | 66.0 | 0 | NA | 8.9 | 0 |
| 77 | 1 | 8.9 | 0 | 43.65 | 0 | NA | 8.5 | 0 |
| 77 | 1 | 12.2 | 0 | 61.7 | 0 | NA | 9.1 | 0 |
| 77 | 1 | 14.8 | 0 | 66.0 | 0 | NA | 7.5 | 0 |
| 77 | 1 | 17.7 | 2 | 66.0 | 0.00014 | (0.0001-0.0002) | 9 | 2.00 |
| 77 | 1 | 20.5 | 0 | 66.0 | 0 | NA | 8 | 0 |
| 78 | 0 | -0.2 | 9 | 66.0 | 0.00034 | (0.0002-0.0005) | 8.6 | 1.6 |
| 78 | 0 | 0.8 | 0 | 66.0 | 0 | NA | 8.3 | 0 |
| 78 | 0 | 2.6 | 0 | 66.0 | 0 | NA | 8.6 | 0 |
| 78 | 0 | 6.0 | 0 | 47.6 | 0 | NA | 8.6 | 0 |
| 78 | 0 | 9.1 | 0 | 40.4 | 0 | NA | 8.5 | 0 |
| 78 | 0 | 12.7 | 0 | 43.6 | 0 | NA | 8.2 | 0 |
| 78 | 0 | 15.9 | 0 | 33.6 | 0 | NA | 8.8 | 0 |
| 78 | 0 | 18.5 | 0 | 40.4 | 0 | NA | 8.3 | 0 |
| 79 | 1 | -0.1 | 15 | 10.4 | 0.00870 | (0.0062-0.0112) | 8.2 | 3.8 |

© 2019 Reinert T et al. *JAMA Oncology.*

## eTable 5. ctDNA Results for All 795 Plasma Samples

| Patient ID | Recurrence | Time post surgery (months)[a] | # ctDNA positive targets | Input DNA (ng) | VAF (mean) | VAF CI 95% | Plasma (mL) | ctDNA copies per mL plasma |
|---|---|---|---|---|---|---|---|---|
| 79 | 1 | 0.6 | 15 | 25.6 | 0.00299 | (0.002-0.0039) | 8.5 | 3.1 |
| 79 | 1 | 3.4 | 13 | 31.6 | 0.00124 | (0.0008-0.0017) | 8.2 | 1.6 |
| 79 | 1 | 6.4 | 15 | 40.0 | 0.00330 | (0.0025-0.0042) | 8.5 | 5.3 |
| 79 | 1 | 9.2 | 15 | 24.8 | 0.03044 | (0.0217-0.0393) | 8.9 | 29 |
| 79 | 1 | 12.2 | 15 | 30.8 | 0.04347 | (0.0306-0.0565) | 8 | 57 |
| 79 | 1 | 15.7 | 15 | 25.6 | 0.14889 | (0.1118-0.1863) | 7.8 | 167 |
| 79 | 1 | 17.8 | 6 | 30.8 | 0.00051 | (0.0001-0.0009) | 8 | 0.68 |
| 80 | 0 | -0.2 | 16 | 56.49 | 0.00112 | (0.0008-0.0015) | 9 | 2.4 |
| 80 | 0 | 0.9 | 0 | 66.0 | 0 | NA | 9 | 0 |
| 80 | 0 | 3.1 | 0 | 66.0 | 0 | NA | 8.9 | 0 |
| 80 | 0 | 5.7 | 0 | 66.0 | 0 | NA | 8.8 | 0 |
| 80 | 0 | 9.0 | 2 | 66.0 | 0.00024 | (0.0002-0.0003) | 9 | 0.75 |
| 80 | 0 | 12.0 | 0 | 45.17 | 0 | NA | 8.9 | 0 |
| 80 | 0 | 15.2 | 0 | 48.37 | 0 | NA | 8.5 | 0 |
| 80 | 0 | 18.0 | 0 | 62.02 | 0 | NA | 8.5 | 0 |
| 81 | 0 | -0.2 | 5 | 66.0 | 0.00023 | (0.0001-0.0003) | 8.7 | 0.82 |
| 81 | 0 | 0.6 | 0 | 66.0 | 0 | NA | 8.4 | 0 |
| 81 | 0 | 3.1 | 0 | 66.0 | 0 | NA | 8.1 | 0 |
| 81 | 0 | 5.9 | 0 | 66.0 | 0 | NA | 8.7 | 0 |
| 81 | 0 | 8.9 | 0 | 66.0 | 0 | NA | 7.8 | 0 |
| 81 | 0 | 12.0 | 0 | 66.0 | 0 | NA | 8.9 | 0 |
| 81 | 0 | 14.7 | 0 | 66.0 | 0 | NA | 8.4 | 0 |
| 81 | 0 | 17.8 | 0 | 66.0 | 0 | NA | 9.5 | 0 |
| 82 | 1 | -0.3 | 16 | 13.6 | 0.00880 | (0.0066-0.011) | 8 | 5.1 |
| 82 | 1 | 0.4 | 13 | 25.55 | 0.00291 | (0.0022-0.0036) | 9 | 2.8 |
| 82 | 1 | 3.4 | 14 | 45.41 | 0.00104 | (0.0008-0.0013) | 8.9 | 1.8 |
| 82 | 1 | 7.3 | 15 | 26.58 | 0.00804 | (0.0063-0.0098) | 8.5 | 8.5 |
| 82 | 1 | 11.3 | 15 | 21.6 | 0.03572 | (0.0277-0.0437) | 8.6 | 31 |
| 83 | 0 | -0.2 | 15 | 44.4 | 0.00050 | (0.0003-0.0007) | 8 | 0.93 |
| 83 | 0 | 0.5 | 0 | 47.6 | 0 | NA | 8.5 | 0 |

© 2019 Reinert T et al. *JAMA Oncology.*

## eTable 5. ctDNA Results for All 795 Plasma Samples

| Patient ID | Recurrence | Time post surgery (months)[a] | # ctDNA positive targets | Input DNA (ng) | VAF (mean) | VAF CI 95% | Plasma (mL) | ctDNA copies per mL plasma |
|---|---|---|---|---|---|---|---|---|
| 83 | 0 | 2.8 | 0 | 66.0 | 0 | NA | 9 | 0 |
| 83 | 0 | 6.0 | 0 | 66.0 | 0 | NA | 8.7 | 0 |
| 83 | 0 | 9.2 | 0 | 56.8 | 0 | NA | 8.5 | 0 |
| 83 | 0 | 12.6 | 0 | 66.0 | 0 | NA | 8.5 | 0 |
| 83 | 0 | 14.8 | 0 | 50.8 | 0 | NA | 8.2 | 0 |
| 84 | 0 | -0.2 | 15 | 66.0 | 0.00423 | (0.0031-0.0054) | 8 | 39 |
| 84 | 0 | 0.9 | 0 | 66.0 | 0 | NA | 8.5 | 0 |
| 84 | 0 | 2.9 | 0 | 53.2 | 0 | NA | 8.7 | 0 |
| 84 | 0 | 6.4 | 0 | 40.0 | 0 | NA | 8.5 | 0 |
| 84 | 0 | 8.9 | 0 | 50.0 | 0 | NA | 8 | 0 |
| 84 | 0 | 15.0 | 0 | 37.6 | 0 | NA | 8 | 0 |
| 85 | 1 | -0.2 | 12 | 27.2 | 0.00512 | (0.0034-0.0069) | 8.2 | 5.8 |
| 85 | 1 | 0.4 | 11 | 66.0 | 0.00635 | (0.0048-0.0079) | 8.6 | 18 |
| 85 | 1 | 3.2 | 11 | 60.08 | 0.01166 | (0.009-0.0144) | 7.3 | 33 |
| 85 | 1 | 6.6 | 11 | 66.0 | 0.09014 | (0.073-0.1078) | 8 | 729 |
| 85 | 1 | 8.7 | 11 | 66.0 | 0.39998 | (0.3217-0.4803) | 8 | 4347 |
| 85 | 1 | 12.2 | 11 | 66.0 | 0.53680 | (0.4215-0.6526) | 9.5 | 70790 |
| 86 | 0 | -0.2 | 16 | 52.0 | 0.02194 | (0.0175-0.0265) | 9.1 | 43 |
| 86 | 0 | 0.3 | 0 | 66.0 | 0 | NA | 10 | 0 |
| 87 | 0 | -0.2 | 16 | 35.85 | 0.00378 | (0.0033-0.0043) | 8.4 | 5.5 |
| 87 | 0 | 0.5 | 0 | 49.6 | 0 | NA | 8.5 | 0 |
| 88 | 0 | -0.2 | 8 | 13.52 | 0.00207 | (0.0012-0.0029) | 9 | 1.1 |
| 88 | 0 | 0.5 | 0 | 31.41 | 0 | NA | 9 | 0 |
| 89 | 1 | -0.2 | 0 | 66.0 | 0 | NA | 8.6 | 0 |
| 89 | 1 | 0.5 | 0 | 66.0 | 0 | NA | 9.3 | 0 |
| 90 | 0 | -0.1 | 2 | 62.0 | 0.00210 | (0-0.006) | 8.3 | 0.26 |
| 90 | 0 | 0.3 | 0 | 66.0 | 0 | NA | 9.1 | 0 |
| 91 | 0 | -0.4 | 16 | 66.0 | 0.00173 | (0.0012-0.0023) | 8.5 | 6.4 |
| 91 | 0 | 0.3 | 0 | 66.0 | 0 | NA | 8.5 | 0 |
| 92 | 1 | -0.3 | 13 | 66.0 | 0.00896 | (0.0061-0.0119) | 9.4 | 22 |

© 2019 Reinert T et al. *JAMA Oncology.*

## eTable 5. ctDNA Results for All 795 Plasma Samples

| Patient ID | Recurrence | Time post surgery (months)[a] | # ctDNA positive targets | Input DNA (ng) | VAF (mean) | VAF CI 95% | Plasma (mL) | ctDNA copies per mL plasma |
|---|---|---|---|---|---|---|---|---|
| 92 | 1 | 0.4 | 0 | 66.0 | 0 | NA | 8.8 | 0 |
| 93 | 0 | -0.2 | 14 | 29.6 | 0.00110 | (0.0008-0.0014) | 8.5 | 1.3 |
| 93 | 0 | 0.5 | 0 | 49.2 | 0 | NA | 9 | 0 |
| 95 | 0 | -0.2 | 10 | 39.18 | 0.00064 | (0.0004-0.0009) | 8.9 | 0.86 |
| 95 | 0 | 0.3 | 0 | 66.0 | 0 | NA | 8.5 | 0 |
| 96 | 0 | -0.2 | 14 | 50.4 | 0.00057 | (0.0004-0.0007) | 9 | 1.1 |
| 96 | 0 | 0.4 | 0 | 66.0 | 0 | NA | 9 | 0 |
| 97 | 0 | -0.2 | 10 | 20.8 | 0.00096 | (0.0006-0.0011) | 8 | 0.78 |
| 97 | 0 | 0.4 | 0 | 66.0 | 0 | NA | 8 | 0 |
| 98 | 0 | -0.2 | 0 | 51.2 | 0 | NA | 9 | 0 |
| 98 | 0 | 0.4 | 0 | 66.0 | 0 | NA | 8.2 | 0 |
| 99 | 1 | -0.2 | 13 | 66.0 | 0.00065 | (0.0002-0.0011) | 8.4 | 2.2 |
| 99 | 1 | 0.3 | 0 | 66.0 | 0 | NA | 9.1 | 0 |
| 100 | 0 | -0.2 | 7 | 23.6 | 0.00046 | (0.0003-0.0007) | 8.4 | 0.45 |
| 100 | 0 | 0.3 | 0 | 66.0 | 0 | NA | 8.7 | 0 |
| 101 | 0 | -0.2 | 16 | 66.0 | 0.00292 | (0.0021-0.0038) | 8.5 | 9.7 |
| 101 | 0 | 0.4 | 0 | 66.0 | 0 | NA | 8.2 | 0 |
| 102 | 0 | -0.3 | 13 | 44.0 | 0.00044 | (0.0003-0.0006) | 8.4 | 0.78 |
| 102 | 0 | 0.4 | 0 | 66.0 | 0 | NA | 8.6 | 0 |
| 103 | 1 | -0.1 | 16 | 33.58 | 0.03621 | (0.0219-0.0505) | 8.5 | 49 |
| 103 | 1 | 0.3 | 0 | 66.0 | 0 | NA | 8.8 | 0 |
| 104 | 1 | -0.1 | 16 | 16.29 | 0.00220 | (0.0017-0.0027) | 8.5 | 1.5 |
| 104 | 1 | 0.4 | 4 | 28.15 | 0.00043 | (0.0003-0.0006) | 8.8 | 0.47 |
| 105 | 0 | -0.2 | 16 | 34.0 | 0.00861 | (0.0059-0.0114) | 8.5 | 12 |
| 105 | 0 | 0.4 | 0 | 66.0 | 0 | NA | 8.7 | 0 |
| 106 | 0 | -0.2 | 0 | 33.2 | 0 | NA | 8.5 | 0 |
| 106 | 0 | 0.5 | 0 | 66.0 | 0 | NA | 9 | 0 |
| 107 | 0 | -0.3 | 5 | 35.2 | 0.00045 | (0.0002-0.0007) | 8.5 | 0.65 |
| 107 | 0 | 0.5 | 0 | 66.0 | 0 | NA | 8.7 | 0 |
| 108 | 1 | -0.4 | 3 | 66.0 | 0.00041 | (0.0001-0.0007) | 9 | 1.5 |

© 2019 Reinert T et al. *JAMA Oncology.*

## eTable 5. ctDNA Results for All 795 Plasma Samples

| Patient ID | Recurrence | Time post surgery (months)[a] | # ctDNA positive targets | Input DNA (ng) | VAF (mean) | VAF CI 95% | Plasma (mL) | ctDNA copies per mL plasma |
|---|---|---|---|---|---|---|---|---|
| 108 | 1 | 0.3 | 0 | 66.0 | 0 | NA | 8.8 | 0 |
| 109 | 0 | -0.1 | 6 | 36.04 | 0.00068 | (0.0003-0.0011) | 9 | 0.93 |
| 109 | 0 | 0.3 | 0 | 66.0 | 0 | NA | 8.9 | 0 |
| 110 | 0 | -0.2 | 0 | 62.8 | 0 | NA | 8.7 | 0 |
| 110 | 0 | 0.6 | 0 | 63.6 | 0 | NA | 8.8 | 0 |
| 111 | 0 | 0.0 | 16 | 49.47 | 0.00542 | (0.0023-0.0086) | 8 | 12 |
| 111 | 0 | 3.1 | 0 | 66.0 | 0 | NA | 8 | 0 |
| 111 | 0 | 5.8 | 0 | 66.0 | 0 | NA | 8 | 0 |
| 111 | 0 | 9.3 | 0 | 41.78 | 0 | NA | 9 | 0 |
| 111 | 0 | 11.8 | 0 | 41.17 | 0 | NA | 8.5 | 0 |
| 111 | 0 | 15.5 | 0 | 53.71 | 0 | NA | 8.2 | 0 |
| 111 | 0 | 18.3 | 0 | 51.93 | 0 | NA | 8.8 | 0 |
| 111 | 0 | 21.0 | 0 | 39.2 | 0 | NA | 8 | 0 |
| 111 | 0 | 27.5 | 0 | 21.96 | 0 | NA | 8.5 | 0 |
| 112 | 0 | 0.0 | 3 | 25.32 | 0.00045 | (0.0003-0.0006) | 8 | 0.47 |
| 112 | 0 | 0.9 | 0 | 14.55 | 0 | NA | 8 | 0 |
| 112 | 0 | 3.1 | 0 | 65.59 | 0 | NA | 8 | 0 |
| 112 | 0 | 6.3 | 0 | 66.0 | 0 | NA | 8 | 0 |
| 112 | 0 | 11.2 | 0 | 46.05 | 0 | NA | 8.5 | 0 |
| 112 | 0 | 15.7 | 0 | 66.0 | 0 | NA | 7.9 | 0 |
| 112 | 0 | 18.2 | 0 | 34.65 | 0 | NA | 8 | 0 |
| 112 | 0 | 21.6 | 0 | 25.46 | 0 | NA | 7.8 | 0 |
| 112 | 0 | 27.2 | 0 | 42.97 | 0 | NA | 8 | 0 |
| 113 | 0 | 0.0 | 16 | 40.69 | 0.00320 | (0.0022-0.0043) | 8 | 5.6 |
| 113 | 0 | 1.2 | 0 | 9.51 | 0 | NA | 8 | 0 |
| 113 | 0 | 3.9 | 0 | 37.2 | 0 | NA | 8.8 | 0 |
| 113 | 0 | 6.7 | 0 | 52.0 | 0 | NA | 8.5 | 0 |
| 113 | 0 | 9.5 | 0 | 56.4 | 0 | NA | 8.3 | 0 |
| 113 | 0 | 11.6 | 0 | 51.2 | 0 | NA | 8.3 | 0 |
| 113 | 0 | 15.0 | 0 | 60.8 | 0 | NA | 7.8 | 0 |

© 2019 Reinert T et al. *JAMA Oncology.*

## eTable 5. ctDNA Results for All 795 Plasma Samples

| Patient ID | Recurrence | Time post surgery (months)[a] | # ctDNA positive targets | Input DNA (ng) | VAF (mean) | VAF CI 95% | Plasma (mL) | ctDNA copies per mL plasma |
|---|---|---|---|---|---|---|---|---|
| 113 | 0 | 18.2 | 0 | 43.6 | 0 | NA | 8 | 0 |
| 113 | 0 | 21.3 | 0 | 52.8 | 0 | NA | 9 | 0 |
| 114 | 0 | -0.2 | 4 | 13.59 | 0.00513 | NA | 8 | 2.6 |
| 114 | 0 | 0.7 | 0 | 19.37 | 0 | NA | 8 | 0 |
| 114 | 0 | 3.2 | 0 | 66.0 | 0 | NA | 8.8 | 0 |
| 114 | 0 | 6.0 | 0 | 54.4 | 0 | NA | 7.8 | 0 |
| 114 | 0 | 9.0 | 0 | 52.24 | 0 | NA | 8 | 0 |
| 114 | 0 | 12.7 | 0 | 65.62 | 0 | NA | 8.6 | 0 |
| 114 | 0 | 15.2 | 0 | 66.0 | 0 | NA | 8.5 | 0 |
| 114 | 0 | 18.0 | 0 | 57.84 | 0 | NA | 6.5 | 0 |
| 115 | 0 | 0.0 | 12 | 66.0 | 0.00067 | (0.0003-0.001) | 8.8 | 1.9 |
| 115 | 0 | 0.7 | 0 | 56.82 | 0 | NA | 8.5 | 0 |
| 115 | 0 | 2.9 | 0 | 49.39 | 0 | NA | 7.8 | 0 |
| 115 | 0 | 5.7 | 0 | 60.52 | 0 | NA | 8 | 0 |
| 115 | 0 | 8.5 | 0 | 54.15 | 0 | NA | 7.7 | 0 |
| 115 | 0 | 11.9 | 0 | 59.2 | 0 | NA | 8.5 | 0 |
| 115 | 0 | 15.1 | 0 | 66.0 | 0 | NA | 8.3 | 0 |
| 115 | 0 | 18.1 | 0 | 66.0 | 0 | NA | 8.5 | 0 |
| 116 | 0 | -0.1 | 10 | 55.23 | 0.00076 | (0.0006-0.0009) | 9 | 1.6 |
| 116 | 0 | 0.5 | 0 | 66.0 | 0 | NA | 8.5 | 0 |
| 116 | 0 | 3.5 | 0 | 50.35 | 0 | NA | 7.5 | 0 |
| 116 | 0 | 6.5 | 0 | 66.0 | 0 | NA | 8 | 0 |
| 116 | 0 | 9.3 | 0 | 66.0 | 0 | NA | 9 | 0 |
| 116 | 0 | 12.0 | 0 | 66.0 | 0 | NA | 8.5 | 0 |
| 116 | 0 | 15.0 | 0 | 59.45 | 0 | NA | 7.5 | 0 |
| 116 | 0 | 18.2 | 0 | 65.2 | 0 | NA | 9 | 0 |
| 117 | 0 | -0.1 | 6 | 30.74 | 0.00064 | (0.0002-0.0011) | 7.5 | 0.89 |
| 117 | 0 | 0.7 | 0 | 66.0 | 0 | NA | 4.3 | 0 |
| 118 | 0 | 0.0 | 14 | 27.2 | 0.00142 | (0.0007-0.0021) | 8.6 | 1.51 |
| 118 | 0 | 0.9 | 0 | 63.6 | 0 | NA | 8.5 | 0 |

© 2019 Reinert T et al. *JAMA Oncology.*

## eTable 5. ctDNA Results for All 795 Plasma Samples

| Patient ID | Recurrence | Time post surgery (months)[a] | # ctDNA positive targets | Input DNA (ng) | VAF (mean) | VAF CI 95% | Plasma (mL) | ctDNA copies per mL plasma |
|---|---|---|---|---|---|---|---|---|
| 118 | 0 | 3.0 | 0 | 66.0 | 0 | NA | 8.4 | 0 |
| 118 | 0 | 5.7 | 0 | 66.0 | 0 | NA | 8.8 | 0 |
| 118 | 0 | 9.0 | 0 | 66.0 | 0 | NA | 6.5 | 0 |
| 118 | 0 | 12.4 | 0 | 61.2 | 0 | NA | 7 | 0 |
| 118 | 0 | 15.1 | 0 | 66.0 | 0 | NA | 8.2 | 0 |
| 119 | 1 | -0.1 | 15 | 59.6 | 0.00118 | (0.0009-0.0015) | 8.5 | 2.8 |
| 119 | 1 | 0.9 | 12 | 66.0 | 0.00054 | (0.0003-0.0007) | 8.5 | 4.3 |
| 119 | 1 | 3.5 | 6 | 50.4 | 0.00026 | (0.0002-0.0004) | 8.2 | 0.53 |
| 119 | 1 | 6.2 | 0 | 66.0 | 0 | NA | 8.4 | 0 |
| 119 | 1 | 11.1 | 8 | 66.0 | 0.00022 | (0.0002-0.0003) | 8.7 | 0.61 |
| 119 | 1 | 13.4 | 13 | 60.8 | 0.00075 | (0.0005-0.001) | 9 | 1.7 |
| 120 | 0 | 0.0 | 14 | 17.6 | 0.00654 | (0.0044-0.0087) | 8 | 4.9 |
| 120 | 0 | 0.5 | 0 | 14.0 | 0 | NA | 8.1 | 0 |
| 120 | 0 | 3.4 | 0 | 66.0 | 0 | NA | 7 | 0 |
| 120 | 0 | 6.4 | 0 | 66.0 | 0 | NA | 8.2 | 0 |
| 120 | 0 | 9.0 | 0 | 19.6 | 0 | NA | 8.5 | 0 |
| 121 | 0 | 0.0 | 0 | 66.0 | 0 | NA | 9.2 | 0 |
| 121 | 0 | 0.8 | 0 | 66.0 | 0 | NA | 7.1 | 0 |
| 122 | 0 | 0.0 | 11 | 66.0 | 0.00060 | (0.0004-0.0008) | 8.4 | 7.7 |
| 122 | 0 | 0.7 | 0 | 66.0 | 0 | NA | 8.5 | 0 |
| 123 | 0 | 0.0 | 5 | 42.13 | 0.00054 | (0.0001-0.001) | 8.5 | 0.53 |
| 123 | 0 | 0.4 | 0 | 66.0 | 0 | NA | 8.5 | 0 |
| 123 | 0 | 3.0 | 0 | 66.0 | 0 | NA | 8.1 | 0 |
| 123 | 0 | 6.2 | 0 | 66.0 | 0 | NA | 8.5 | 0 |
| 123 | 0 | 9.2 | 0 | 35.58 | 0 | NA | 7.8 | 0 |
| 123 | 0 | 11.7 | 0 | 48.02 | 0 | NA | 8.5 | 0 |
| 124 | 1 | 0.0 | 16 | 66.0 | 0.04668 | (0.0396-0.0542) | 9 | 375 |
| 124 | 1 | 0.5 | 13 | 48.87 | 0.00074 | (0.0005-0.0009) | 8 | 1.5 |
| 124 | 1 | 3.1 | 7 | 43.62 | 0.00054 | (0.0003-0.0007) | 8.4 | 0.91 |
| 124 | 1 | 5.8 | 13 | 39.78 | 0.00063 | (0.0005-0.0008) | 9 | 0.96 |

© 2019 Reinert T et al. *JAMA Oncology.*

## eTable 5. ctDNA Results for All 795 Plasma Samples

| Patient ID | Recurrence | Time post surgery (months)[a] | # ctDNA positive targets | Input DNA (ng) | VAF (mean) | VAF CI 95% | Plasma (mL) | ctDNA copies per mL plasma |
|---|---|---|---|---|---|---|---|---|
| 124 | 1 | 8.8 | 16 | 51.85 | 0.03123 | (0.026-0.0367) | 8.5 | 65 |
| 124 | 1 | 12.0 | 16 | 44.17 | 0.23820 | (0.2037-0.2736) | 9 | 398 |
| 125 | 1 | -0.2 | 15 | 32.16 | 0.00140 | (0.0011-0.0017) | 8.5 | 1.8 |
| 125 | 1 | 0.7 | 0 | 38.8 | 0 | NA | 7.5 | 0 |
| 126 | 0 | -0.2 | 13 | 51.65 | 0.00772 | (0.0028-0.0131) | 8.2 | 17 |
| 126 | 0 | 0.5 | 0 | 44.72 | 0 | NA | 7.6 | 0 |
| 127 | 0 | 0.0 | 15 | 66.0 | 0.00136 | (0.0008-0.002) | 9.1 | 12 |
| 127 | 0 | 0.6 | 0 | 66.0 | 0 | NA | 8.3 | 0 |
| 128 | 0 | 21.3 | 0 | 62.4 | 0 | NA | 8.5 | 0 |
| 128 | 0 | 24.0 | 0 | 42.4 | 0 | NA | 8.5 | 0 |
| 128 | 0 | 27.0 | 0 | 62.4 | 0 | NA | 8.3 | 0 |
| 128 | 0 | 30.0 | 0 | 44.8 | 0 | NA | 8.4 | 0 |
| 128 | 0 | 33.2 | 0 | 57.2 | 0 | NA | 8.4 | 0 |
| 128 | 0 | 36.6 | 0 | 46.0 | 0 | NA | 8.1 | 0 |
| 130 | 0 | 0.0 | 14 | 50.48 | 0.00099 | (0.0006-0.0014) | 9.6 | 1.9 |
| 130 | 0 | 0.4 | 2 | 66.0 | 0.00019 | (0-0.0005) | 9.8 | 0.98 |

[a]A negative value indicates that the blood was drawn on a day prior to surgery. A null value indicates that the sampling timepoint is on the same day as surgery, but prior to surgery.

[b]Not applicable

© 2019 Reinert T et al. *JAMA Oncology.*

| **eTable 6. Recurrence-Free Survival Analysis by Clinicopathological Variables and Post-op ctDNA Status at Day 30** | | | | | | | |
|---|---|---|---|---|---|---|---|
| **Variable** | **Univariate analysis** | | | **Multivariate analysis** | | | |
| | **HR** | **(95% CI)** | ***P*-value** | **HR** | **(95% CI)** | ***P*-value** | **Schoenfeld *P*-value** |
| **All patients with a day 30 postoperative sample (n = 94)** | | | | | | | |
| **Age** | | | | | | | |
| < mean versus ≥ Mean | 1.4 | (0.52-3.8) | .51 | | | | |
| **Stage** | | | | | | | |
| Stage II versus stage III | 5.3 | (1.2-23.0) | **.028** | 2.4 | (0.48-12.2) | .29 | |
| **Tumor site** | | | | | | | |
| Right versus left | 1.9 | (0.72-5.3) | .19 | | | | |
| **Lymphovascular invasion** | | | | | | | |
| No versus yes | 2.7 | (1.0-7.0) | **.044** | 1.9 | (0.63-5.6) | .26 | |
| **MMR-status** | | | | | | | |
| Deficient versus proficient | 0.45 | (0.059-3.4) | .43 | | | | |
| **Radical resection (micro)** | | | | | | | |
| Yes versus no | 2.3 | (0.72-7.2) | .16 | | | | |
| **Histology** | | | | | | | |
| Adeno- versus mucinouscarcinoma | 1.6 | (0.35-7.2) | .53 | | | | |
| **Tumor differentiation** | | | | | | | |
| Medium/well versus poor | 0.86 | (0.19-3.8) | .85 | | | | |
| **Gender** | | | | | | | |
| Female versus male | 0.24 | (0.079-0.74) | **.013**[a] | .21 | (0.06-0.69) | **.010** | |
| **ctDNA status up to 6 weeks after OP (no ACT)** | | | | | | | |
| ctDNA- versus ctDNA+ | 7.2 | (2.7-19.0) | **<.001** | 4.5 | (1.6-12.8) | **.004** | |
| **Global goodness-of fit test for Cox proportional hazards models** | | | | | | | .22 |

[a]The present consecutive cohort unexpectedly has an extreme high proportion of relapse events among women 28.8% (15/52) and an a low proportion among men 12.3% (9/73). In the Danish population the overall relapse rates in women and men, in the period from 2001 to 2011, were 22% (2233/9958) and 25% (2803/11194), respectively.[13] Hence, the association between female sex and relapse observed in the present cohort is likely false.

© 2019 Reinert T et al. *JAMA Oncology.*

**eTable 7. Recurrence-Free Survival Analysis by Clinicopathological Variables, Post-op ctDNA, and Post-op CEA Status at First Timepoint Post-ACT**

| Variable | Univariate analysis | | | Multivariate analysis | | | |
|---|---|---|---|---|---|---|---|
| | HR | (95% CI) | *P*-value | HR | (95% CI) | *P*-value | Schoenfeld *P*-value |
| **All patients with longitudinally collected plasma and ACT (n=58)** | | | | | | | |
| **Age** | | | | | | | |
| < mean versus ≥ Mean | 1.3 | (0.44-3.8) | .61 | | | | |
| **Stage** | | | | | | | |
| Stage II versus stage III | 1.3 | (0.17-10.0) | .79 | | | | |
| **Tumor site** | | | | | | | |
| Right versus left | 1.8 | (0.63-5.3) | .27 | | | | |
| **Lymphovascular invasion** | | | | | | | |
| No versus yes | 3.0 | (0.93-9.5) | .066 | | | | |
| **MMR-status** | | | | | | | |
| Deficient versus proficient | 1.1 | (0.0-Inf) | >.99 | | | | |
| **Radical resection (micro)** | | | | | | | |
| Yes versus no | 2.6 | (0.77-9) | .13 | | | | |
| **Histology** | | | | | | | |
| Adeno- versus mucinouscarcinoma | 1.1 | (.14-8.3) | .94 | | | | |
| **Tumor differentiation** | | | | | | | |
| Medium/well versus poor | 1.2 | (0.27-5.6) | .78 | | | | |
| **Tumor perforation** | | | | | | | |
| No versus yes | 0.91 | (0.12-7) | .93 | | | | |
| **Gender** | | | | | | | |
| Female versus male | 0.2 | (0.055-0.71) | **.013**[a] | 0.37 | (0.09-1.5) | .16 | |
| **CEA** | | | | | | | |
| CEA- versus CEA+ | 2.4 | (0.74-7.9) | .14 | | | | |
| **ctDNA** | | | | | | | |
| ctDNA- versus ctDNA+ | 17.5 | (5.4-56.5) | **<.001** | 11.8 | (3.4-40.8) | **<.001** | |
| **Global goodness-of fit test for Cox proportional hazards models** | | | | | | | .80 |

© 2019 Reinert T et al. *JAMA Oncology.*

[a]The present consecutive cohort unexpectedly has an extreme high proportion of relapse events among women 28.8% (15/52) and an a low proportion among men 12.3% (9/73). In the Danish population the overall relapse rates in women and men, in the period from 2001 to 2011, were 22% (2233/9958) and 25% (2803/11194), respectively.[13] Hence, the association between female sex and relapse observed in the present cohort is likely false.

**eTable 8. Matched Tumor and Metastatic WES**

| | Patient ID | | |
|---|---|---|---|
| | 20[a] | 24[a] | 77[b] |
| Mutations present in tumor also present in the metastasis (%) | 62.6 | 50.0 | 79.4 |
| Mutations screened in plasma also present in the metastasis (%) | 100.0 | 87.5 | 93.8 |

[a]No ctDNA detected
[b]ctDNA detected after relapse

© 2019 Reinert T et al. *JAMA Oncology.*

**eTable 9. Recurrence-Free Survival Analysis by Clinicopathological Variables and Post-op ctDNA and CEA Status in Surveillance Samples**

| Variable | Univariate analysis | | | Multivariate analysis | | | |
|---|---|---|---|---|---|---|---|
| | HR | (95% CI) | *P*-value | HR | (95% CI) | *P*-value | *Schoenfeld P-value* |
| **All patients with longitudinally collected plasma (n=75)** | | | | | | | |
| **Age** | | | | | | | |
| < mean versus ≥ Mean | 1.0 | (0.39-2.8) | .95 | | | | |
| **Stage** | | | | | | | |
| Stage II versus stage III | 3.3 | (0.74-15) | .12 | | | | |
| **Tumor site** | | | | | | | |
| Right versus left | 1.2 | (0.45-3.2) | .72 | | | | |
| **Lymphovascular invasion** | | | | | | | |
| No versus yes | 4.1 | (1.4-12) | **.010** | 1.1 | (0.35-3.4) | .87 | |
| **MMR-status** | | | | | | | |
| Deficient versus proficient | 1.2 | (0.0-Inf) | >.99 | | | | |
| **Radical resection (micro)** | | | | | | | |
| Yes versus no | 2.9 | (0.88-9.3) | .080 | | | | |
| **Histology** | | | | | | | |
| Adeno- versus mucinous carcinoma | 0.84 | (.84-6.4) | .87 | | | | |
| **Tumor differentiation** | | | | | | | |
| Medium/well versus poor | 0.92 | (0.21-4.1) | .91 | | | | |
| **Tumor perforation** | | | | | | | |
| No versus yes | 1.1 | (0.15-8.7) | .89 | | | | |
| **Gender** | | | | | | | |
| Female versus male | 0.25 | (0.08-0.8) | **.018**[a] | 0.94 | (0.28-3.2) | .92 | |
| **CEA** | | | | | | | |
| CEA- versus CEA+ | 2.8 | (0.95-8.0) | .061 | | | | |
| **ctDNA** | | | | | | | |
| ctDNA- versus ctDNA+ | 43.5 | (9.8-193.5) | **<.001** | 39.9 | (7.5-211.0) | **<.001** | |
| **Global goodness-of fit test for Cox proportional hazards models** | | | | | | | .84 |

[a]The present consecutive cohort unexpectedly has an extreme high proportion of relapse events among women 28.8% (15/52) and an a low proportion among men 12.3% (9/73). In the Danish population the overall relapse rates in women and men, in the period from 2001 to 2011, were 22% (2233/9958) and 25% (2803/11194), respectively.[13] Hence, the association between female sex and relapse observed in the present cohort is likely false.

© 2019 Reinert T et al. *JAMA Oncology.*

## eTable 10. Patients With Actionable Mutations Detected in the Primary Tumor

| Pt. ID | Chr | Gene | POS (Hg19) | SNP ID | Nucleotide | Alt. nucleotide | POS change | Aminoacid change | Cancer type | Drug | Database | ctDNA+ longitudinal plasma |
|---|---|---|---|---|---|---|---|---|---|---|---|---|
| 42 | chr14 | *AKT1* | 105246551 | rs121434592 | C | T | c.49G>A | p.Glu17Lys | Solid Tumors | AZD5363 | https://www.mycancergenome.org/ | |
| 42 | chr7 | *BRAF* | 140453136 | rs113488022 | A | T | c.1799T>A | p.Val600Glu | CRC | RTK-Inhibitor+Trametinib | http://oncokb.org/#/actionableGenes | |
| 18 | chr12 | *KRAS* | 25398284 | rs121913529 | C | T | c.35G>A | p.Gly12Asp | All Tumors | GDC-0994, KO-947, LY3214996 | http://oncokb.org/#/actionableGenes | Yes |
| 20 | chr12 | *KRAS* | 25398284 | rs121913529 | C | T | c.35G>A | p.Gly12Asp | All Tumors | GDC-0994, KO-947, LY3214996 | http://oncokb.org/#/actionableGenes | |
| 20 | chr10 | *PTEN* | 89692981 | . | T | TG | c.988dupG | p.Glu330fs | CRC | GSK2636771 & AZD8186 | http://oncokb.org/#/actionableGenes | |
| 68 | chr3 | *PIK3CA* | 178936091 | rs104886003 | G | A | c.1633G>A | p.E545K | All Tumors | AZD8186 | https://www.mycancergenome.org/ | Yes |
| 68 | chr12 | *KRAS* | 25398284 | rs121913529 | C | T | c.35G>A | p.Gly12Asp | All Tumors | GDC-0994, KO-947, LY3214996 | http://oncokb.org/#/actionableGenes | Yes |
| 24 | chr12 | *KRAS* | 25398284 | rs121913529 | C | A | c.35G>T | p.Gly12Val | CRC | GDC-0994, KO-947, LY3214996 | http://oncokb.org/#/actionableGenes | |
| 24 | chr18 | *SMAD4* | 48604706 | . | G | T | c.1528G>T | p.Gly510* | All Tumors | | https://www.mycancergenome.org/ | |
| 29 | chr12 | *KRAS* | 25398281 | rs112445441 | C | T | c.38G>A | p.Gly13Asp | CRC | GDC-0994, KO-947, LY3214996 | http://oncokb.org/#/actionableGenes | Yes |
| 30 | chr3 | *PIK3CA* | 178936082 | . | G | A | c.1624G>A | E542K | CRC/Breast cancer | Aspirin/PI3K inhibitors | https://www.mycancergenome.org/ | Yes |
| 30 | chr12 | *KRAS* | 25398284 | rs121913529 | C | A | c.35G>T | p.Gly12Val | CRC/Breast cancer | GDC-0994, KO-947, LY3214996 | http://oncokb.org/#/actionableGenes | Yes |
| 30 | chr18 | *SMAD4* | 48591928 | rs377767350 | T | G | c.1091T>G | p.Leu364Trp | CRC | | https://www.mycancergenome.org/ | Yes |
| 75 | chr17 | *ERBB2* | 37880261 | . | G | T | c.2305G>T | p.Asp769Tyr | All Tumors | Neratinib/afatinib | https://civicdb.org | Yes |
| 75 | chr17 | *ERBB2* | 37881000 | rs121913471 | G | T | c.2329G>T | p.Val777Leu | All Tumors | Neratinib/afatinib | https://civicdb.org | Yes |
| 77 | chr7 | *BRAF* | 140453136 | rs113488022 | A | T | c.1799T>A | p.Val600Glu | CRC | RTK-Inhibitor+Trametinib | http://oncokb.org/#/actionableGenes | Yes |
| 37 | chr12 | *KRAS* | 25398285 | rs121913530 | C | T | c.34G>A | p.Gly12Ser | CRC | GDC-0994, KO-947, LY3214996 | http://oncokb.org/#/actionableGenes | Yes |
| 89 | chr12 | *KRAS* | 25398285 | rs121913530 | C | A | c.34G>T | p.Gly12Cys | CRC | GDC-0994, KO-947, LY3214996 | http://oncokb.org/#/actionableGenes | |
| 125 | chr12 | *KRAS* | 25398284 | rs121913529 | C | A | c.35G>T | p.Gly12Val | CRC | GDC-0994, KO-947, LY3214996 | http://oncokb.org/#/actionableGenes | |
| 85 | chr3 | *PIK3CA* | 178938934 | . | G | A | c.2176G>A | p.Glu726Lys | CRC | Aspirin/PI3K inhibitors | https://www.mycancergenome.org/ | Yes |
| 85 | chr12 | *KRAS* | 25398285 | rs121913530 | C | A | c.34G>T | p.Gly12Cys | All Tumors | GDC-0994, KO-947, LY3214996 | http://oncokb.org/#/actionableGenes | Yes |
| 119 | chr12 | *KRAS* | 25398285 | rs121913530 | C | T | c.34G>A | p.Gly12Ser | CRC | GDC-0994, KO-947, LY3214996 | http://oncokb.org/#/actionableGenes | Yes |

© 2019 Reinert T et al. *JAMA Oncology.*

| | | | | | | | | | | | | |
|---|---|---|---|---|---|---|---|---|---|---|---|---|
| 99 | chr12 | *KRAS* | 25380278 | . | A | T | c.180T>A | p.Gly60Gly | CRC | GDC-0994, KO-947, LY3214997 | http://oncokb.org/#/actionableGenes | |
| 99 | chr12 | *KRAS* | 25380277 | rs121913238 | G | T | c.181C>A | p.Gln61Lys | CRC | GDC-0994, KO-947, LY3214998 | http://oncokb.org/#/actionableGenes | |
| 104 | chr18 | *SMAD4* | 48604701 | . | G | C | c.1523G>C | p.Gly508Ala | CRC | | https://www.mycancergenome.org/ | Yes |
| 124 | chr7 | *BRAF* | 140453136 | rs113488022 | A | T | c.1799T>A | p.Val600Glu | CRC | RTK-Inhibitor+Trametinib | http://oncokb.org/#/actionableGenes | Yes |
| 108 | chr3 | *PIK3CA* | 178936094 | rs121913286 | C | A | c.1636C>A | Q546K | CRC | Aspirin/PI3K inhibitors | https://www.mycancergenome.org/ | |
| 108 | chr12 | *KRAS* | 25398285 | rs121913530 | C | A | c.34G>T | p.Gly12Cys | CRC | GDC-0994, KO-947, LY3214996 | http://oncokb.org/#/actionableGenes | |

© 2019 Reinert T et al. *JAMA Oncology.*

**eFigure 1. Summary of Clinical, Histopathological, and Molecular Parameters for All 125 Patients.** A) Rate of synonymous and non-synonymous mutations called from WES. B) The relative contribution of the five most prevalent colorectal cancer associated mutational signatures. C) Mutations in frequently mutated genes in colorectal cancer.[11] D) Clinical and histopathological characteristics.

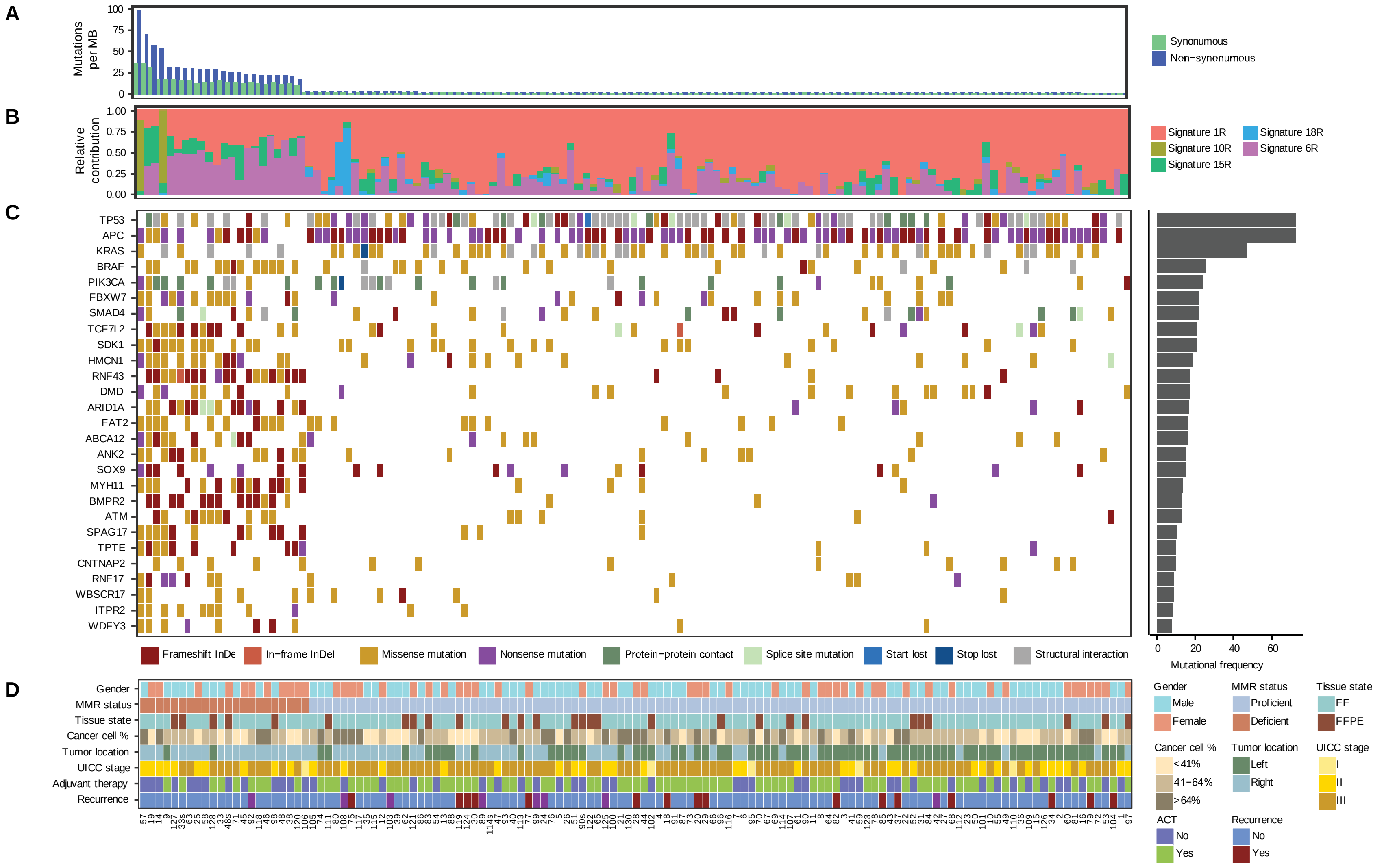


© 2019 Reinert T et al. *JAMA Oncology.*

**eFigure 2. Quality Control Metrics for cfDNA Sequencing Using Multiplex PCR NGS.**
A) DNA input into NGS libraries. A maximum of 66ng was used for library preparation (approximately 20.000 genome equivalents). B) read depth of amplicons. Amplicons with coverage less than 5000x were excluded from analyses and samples with less than 8 passing amplicons (out of 16) failed sequencing coverage QC. C) mean error rates. D) Sequencing sample concordance between plasma samples from the same patient as well as the corresponding tissue biopsy sample. All plasma samples from the relapse patients were tested and no mixup was identified.

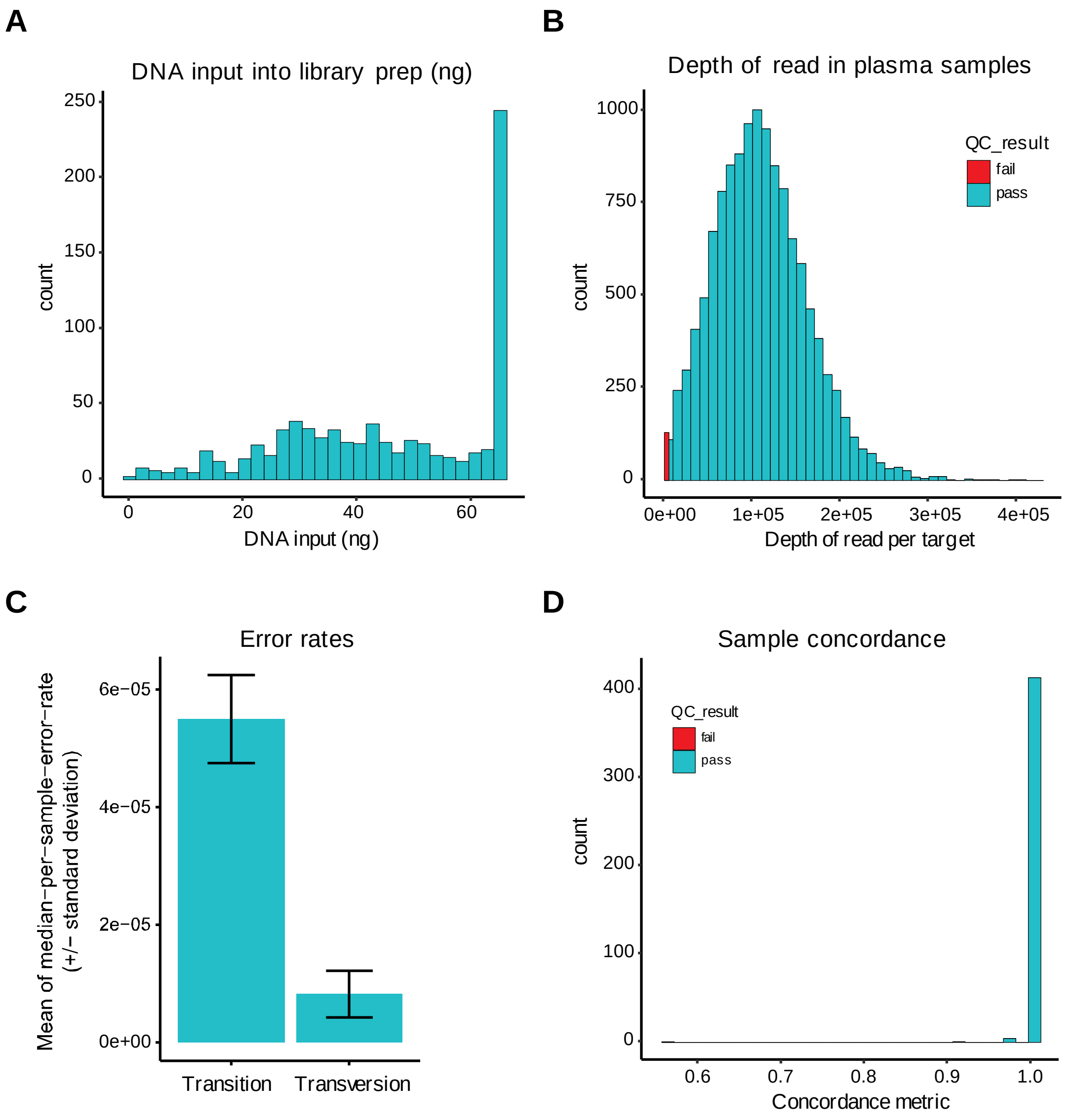


© 2019 Reinert T et al. *JAMA Oncology.*

**eFigure 3. Detailed ctDNA Results and Disease Course Information.** Representation of detailed disease courses, applied treatments and longitudinal ctDNA analyses for all 125 patients. ctDNA is represented as copies/mL plasma for each mutation individually and as a mean of all detected mutations.

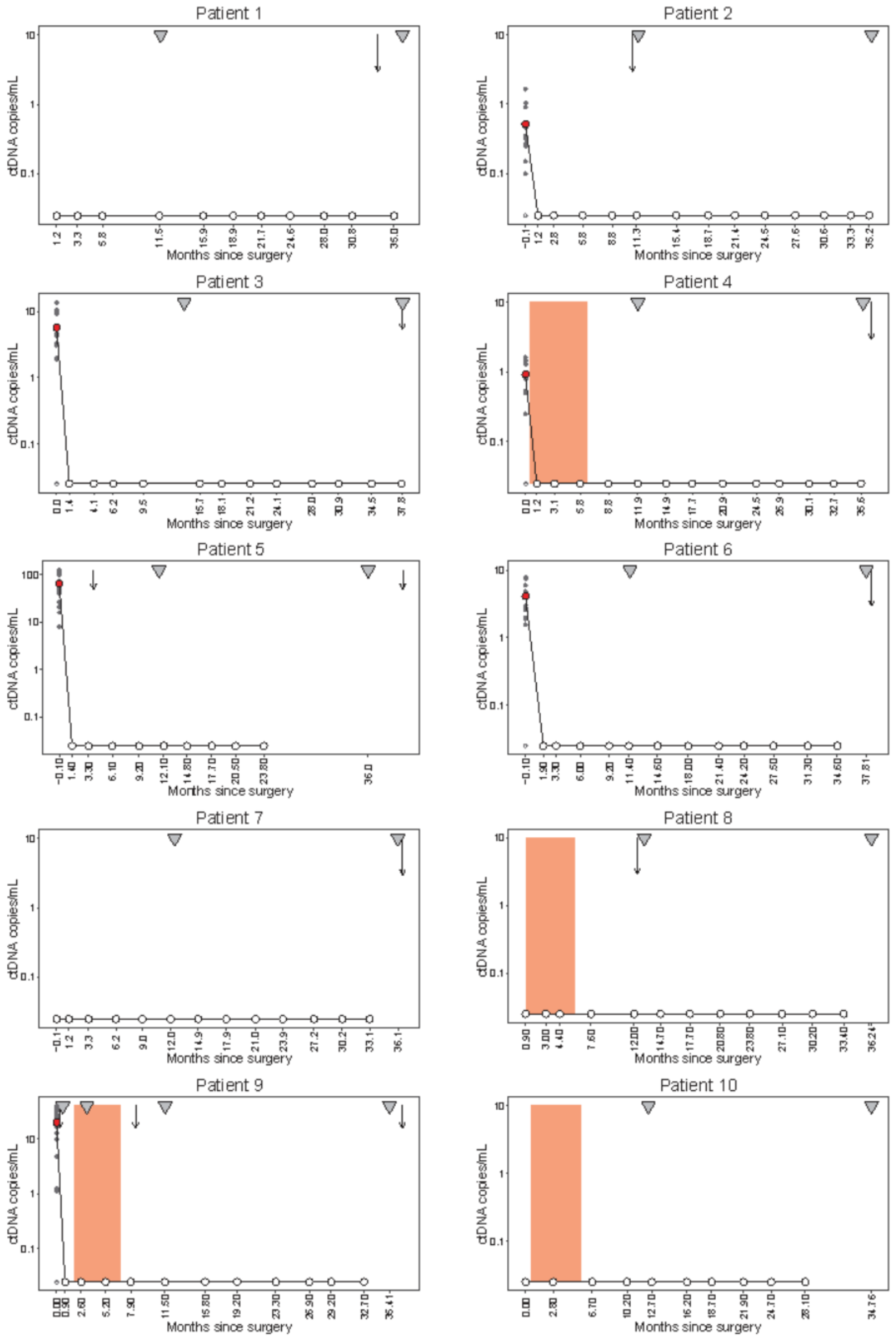


© 2019 Reinert T et al. *JAMA Oncology.*

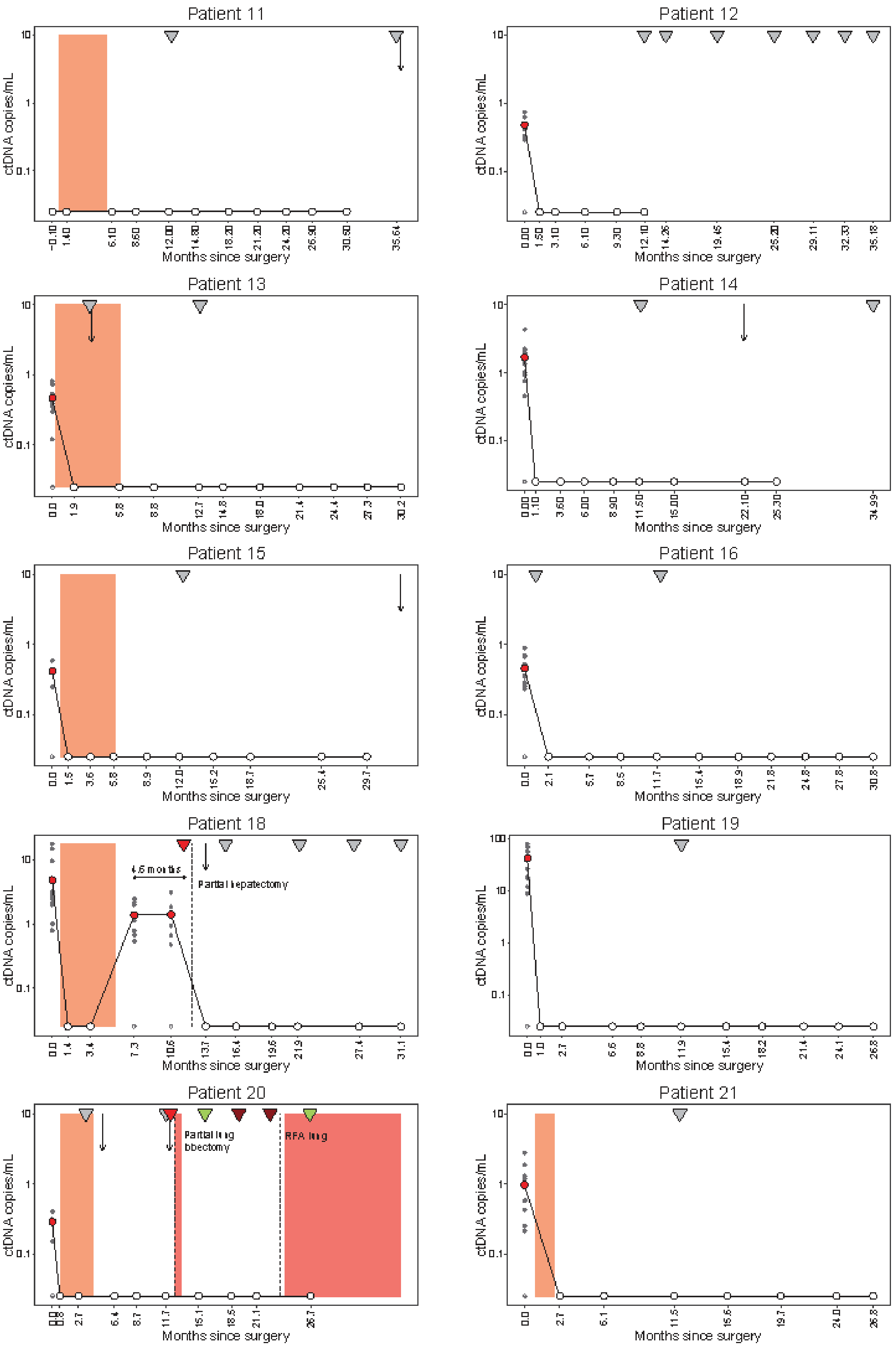


© 2019 Reinert T et al. *JAMA Oncology.*

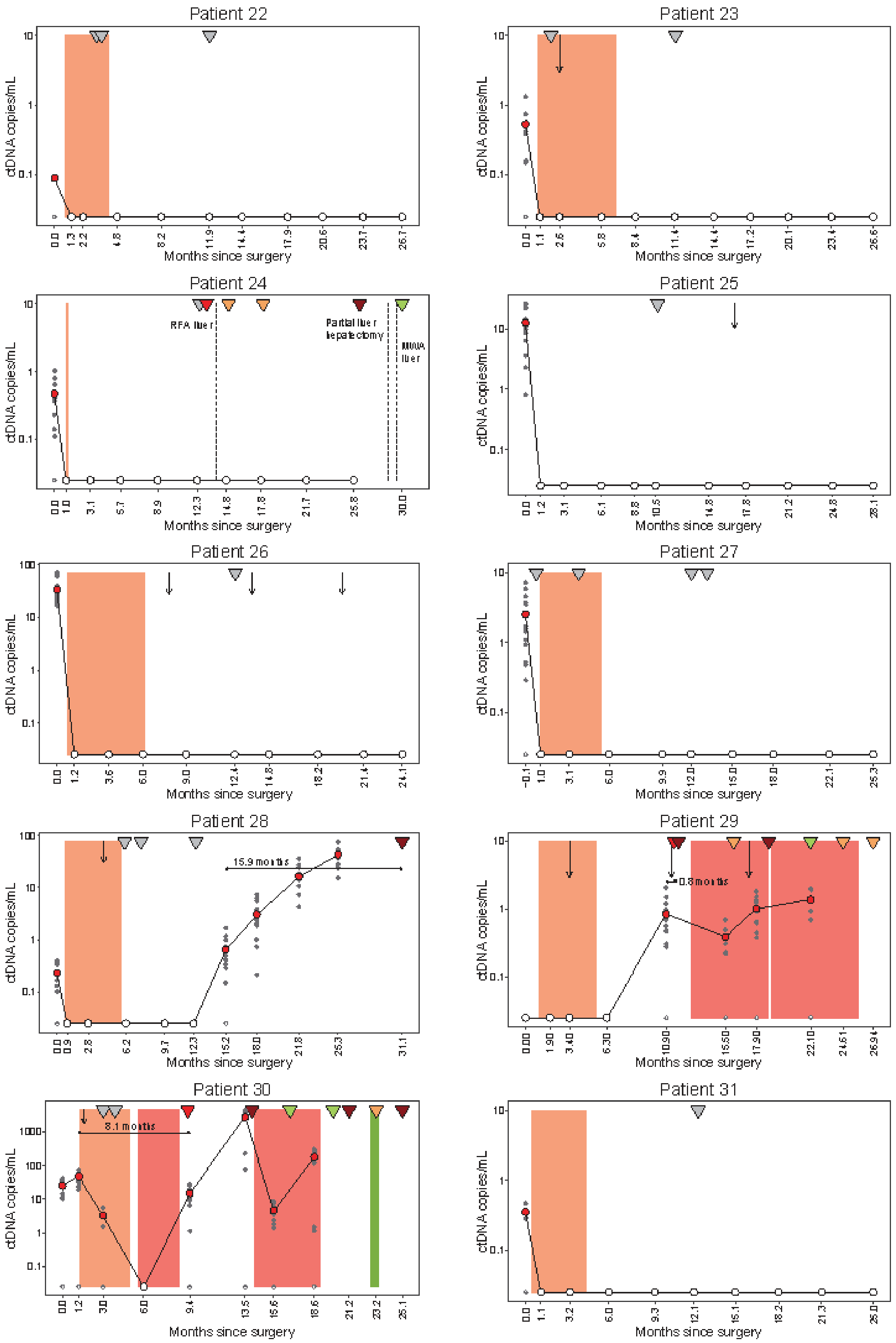


© 2019 Reinert T et al. *JAMA Oncology.*

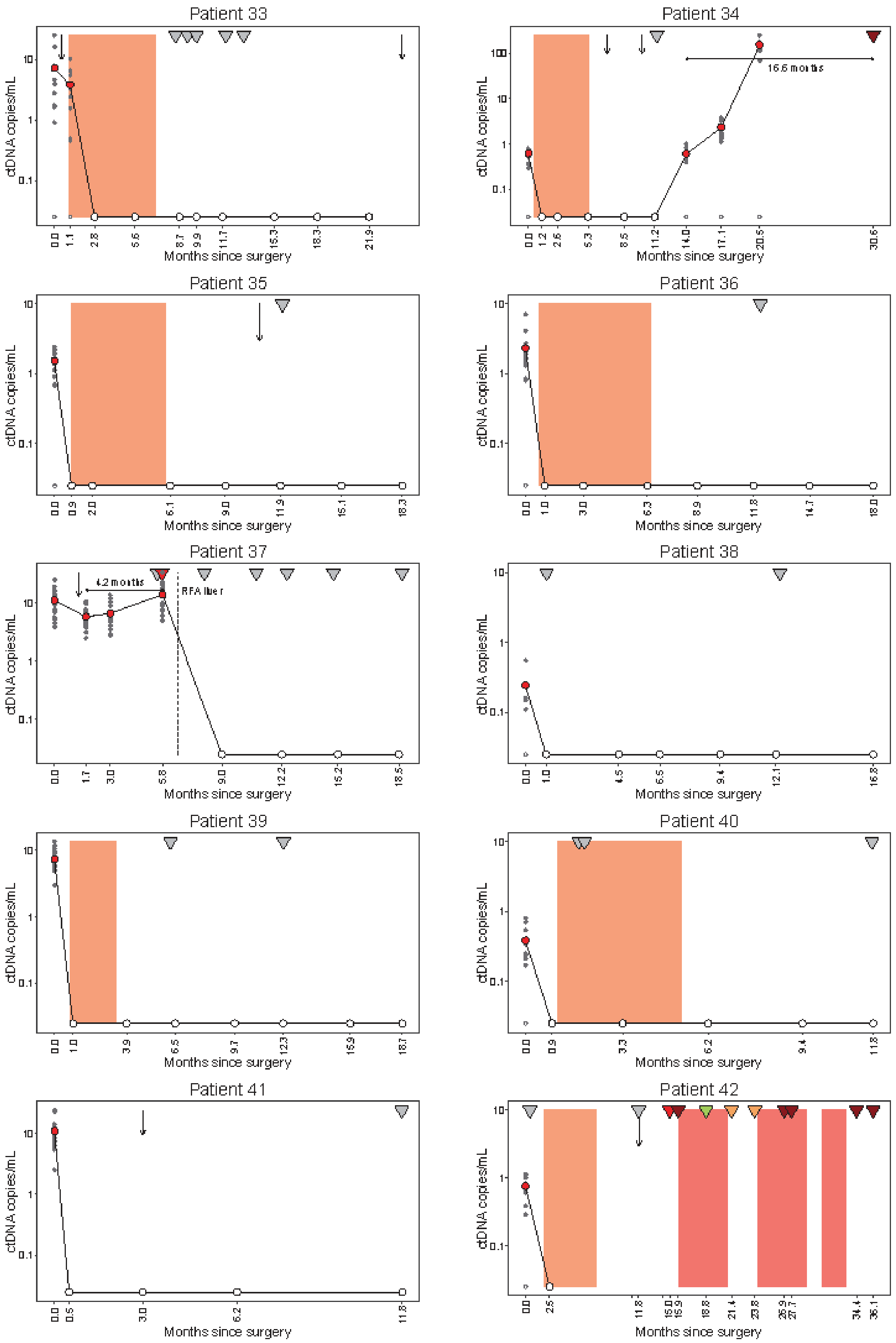


© 2019 Reinert T et al. *JAMA Oncology.*

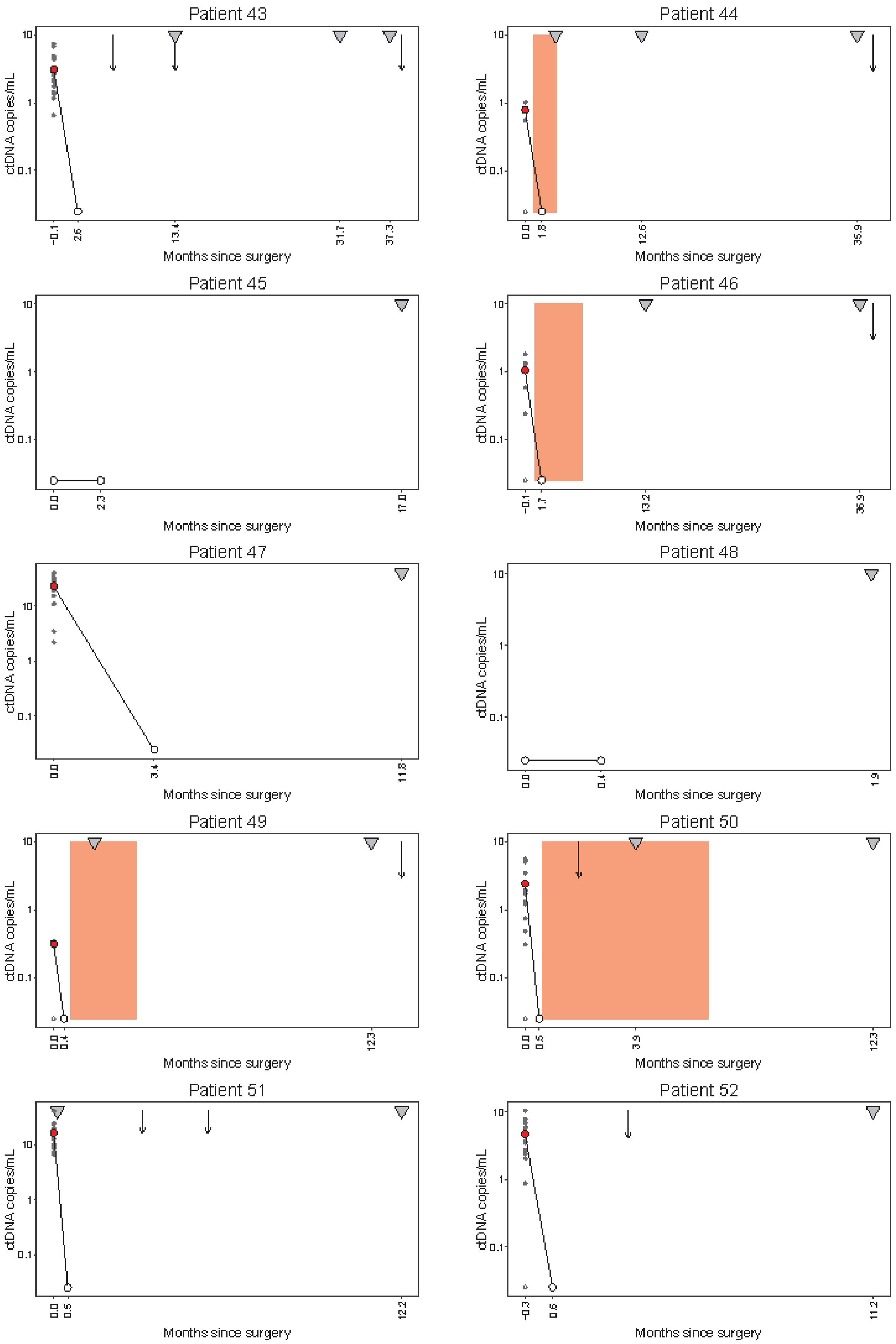


© 2019 Reinert T et al. *JAMA Oncology.*

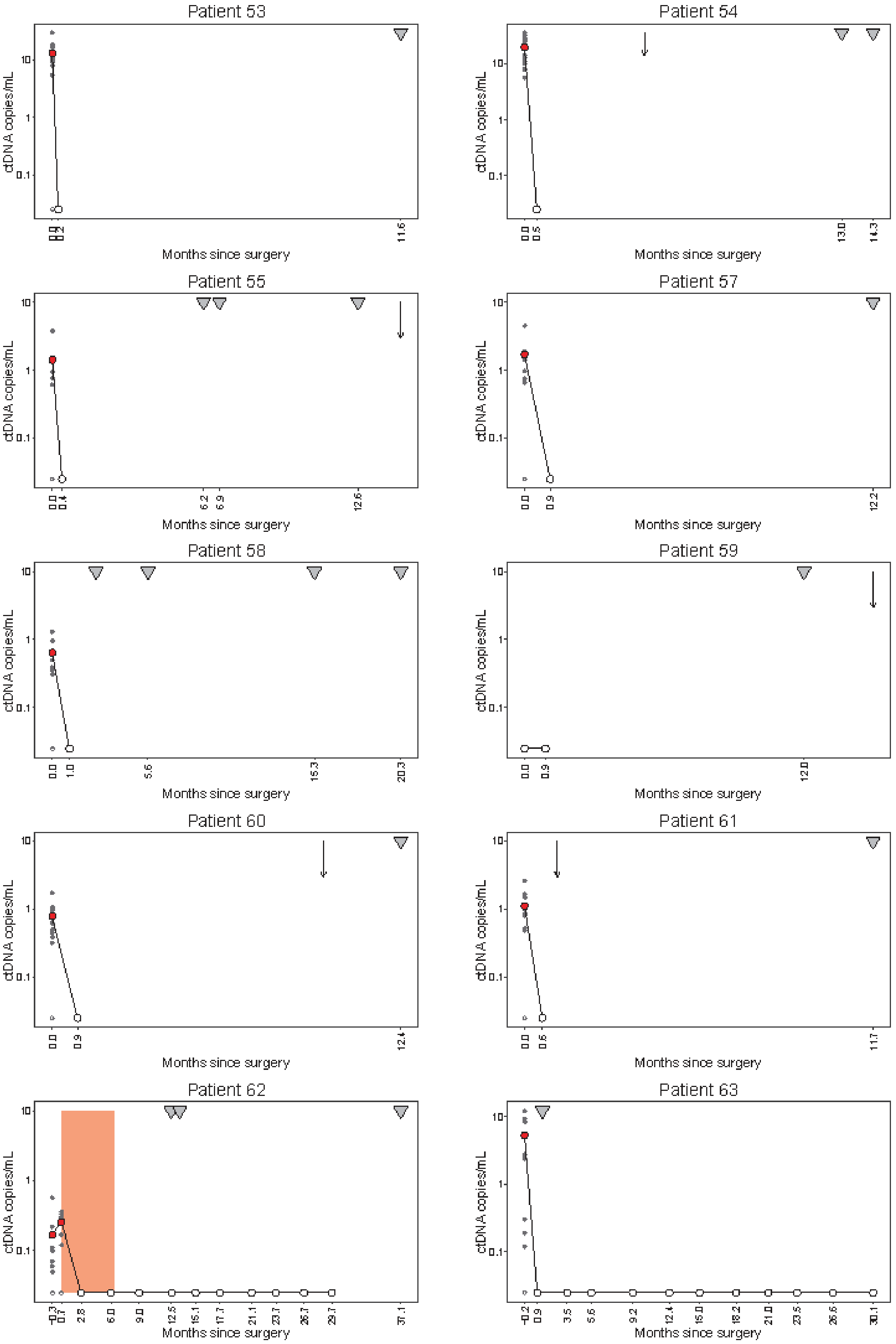


© 2019 Reinert T et al. *JAMA Oncology.*

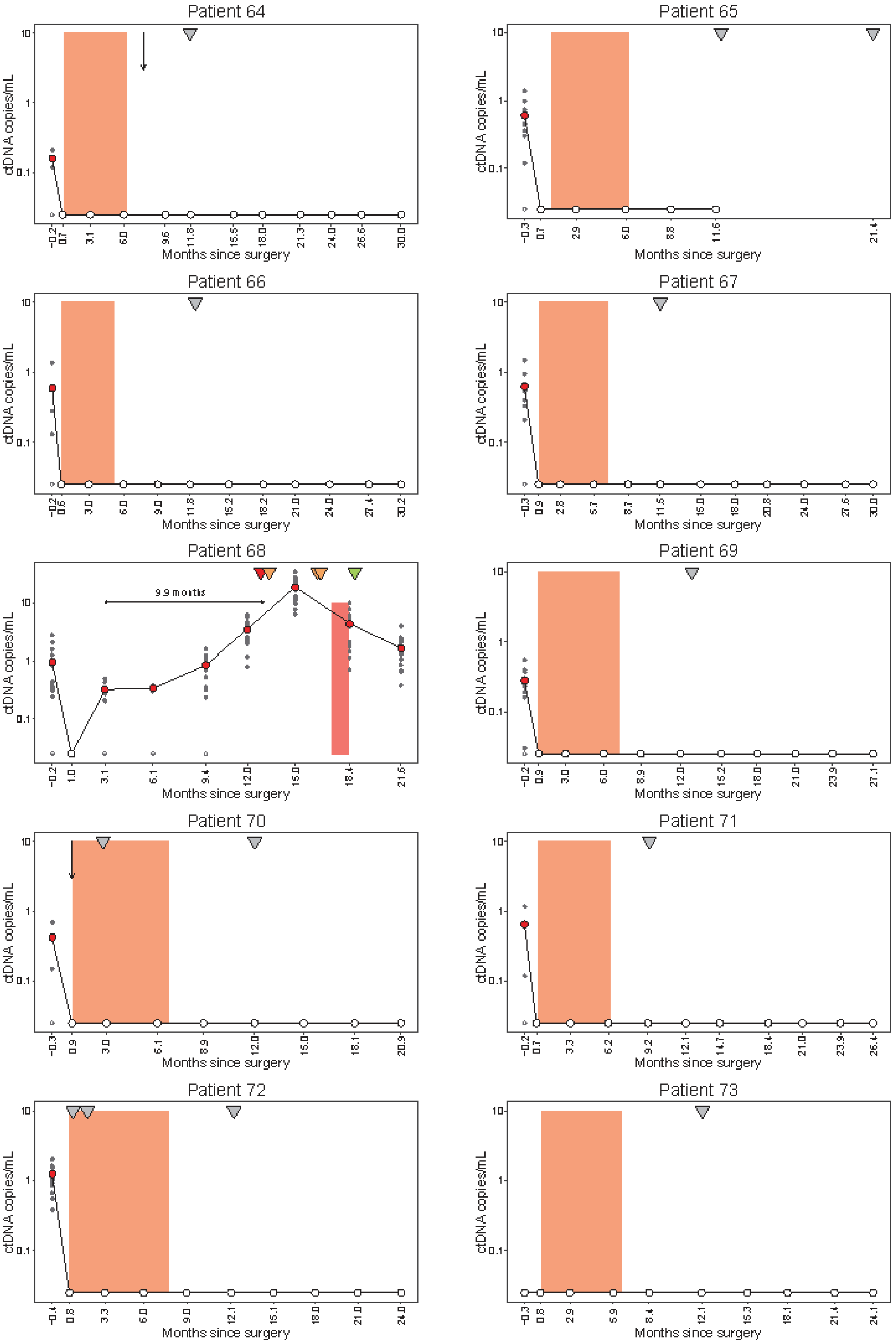


© 2019 Reinert T et al. *JAMA Oncology.*

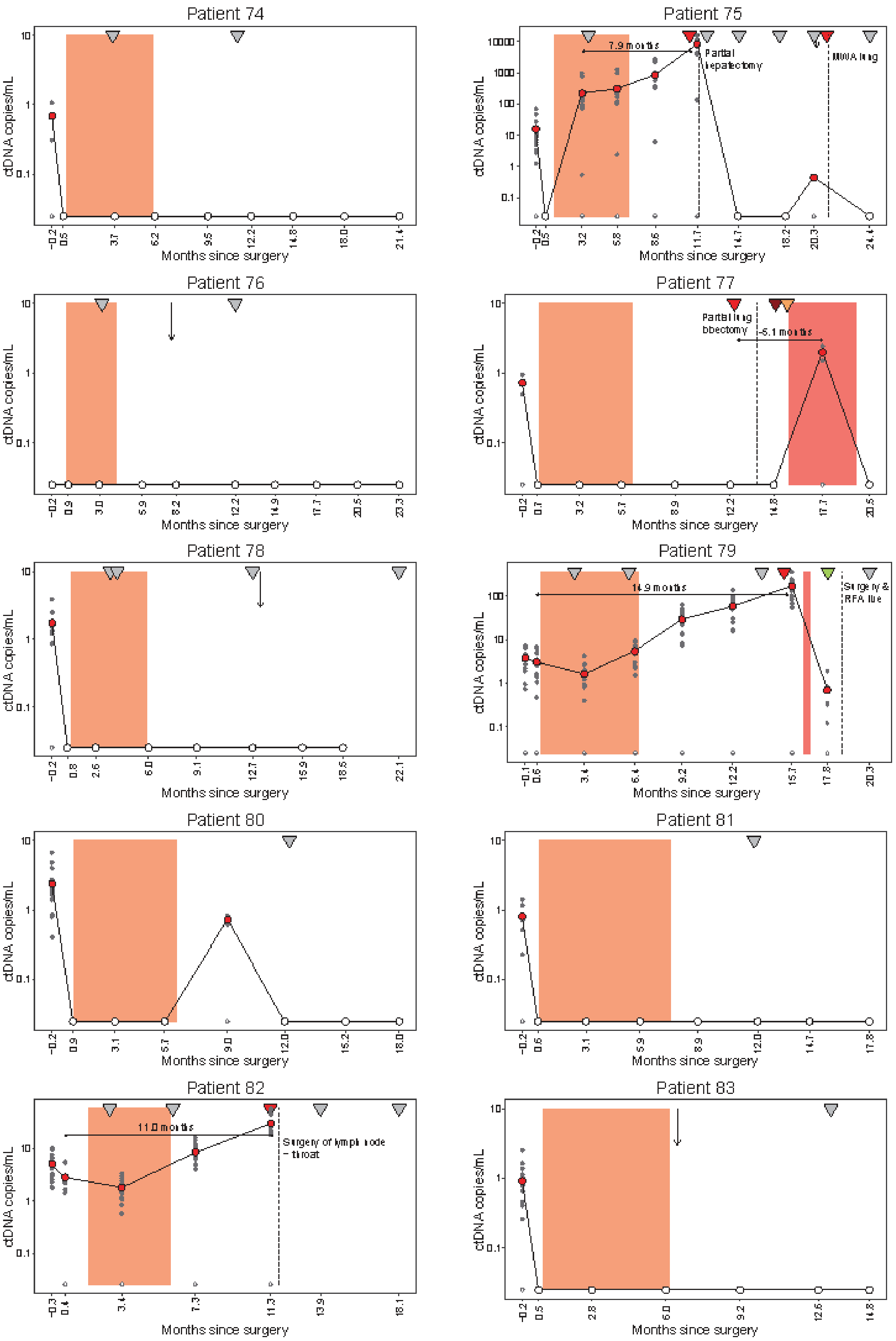


© 2019 Reinert T et al. *JAMA Oncology*.

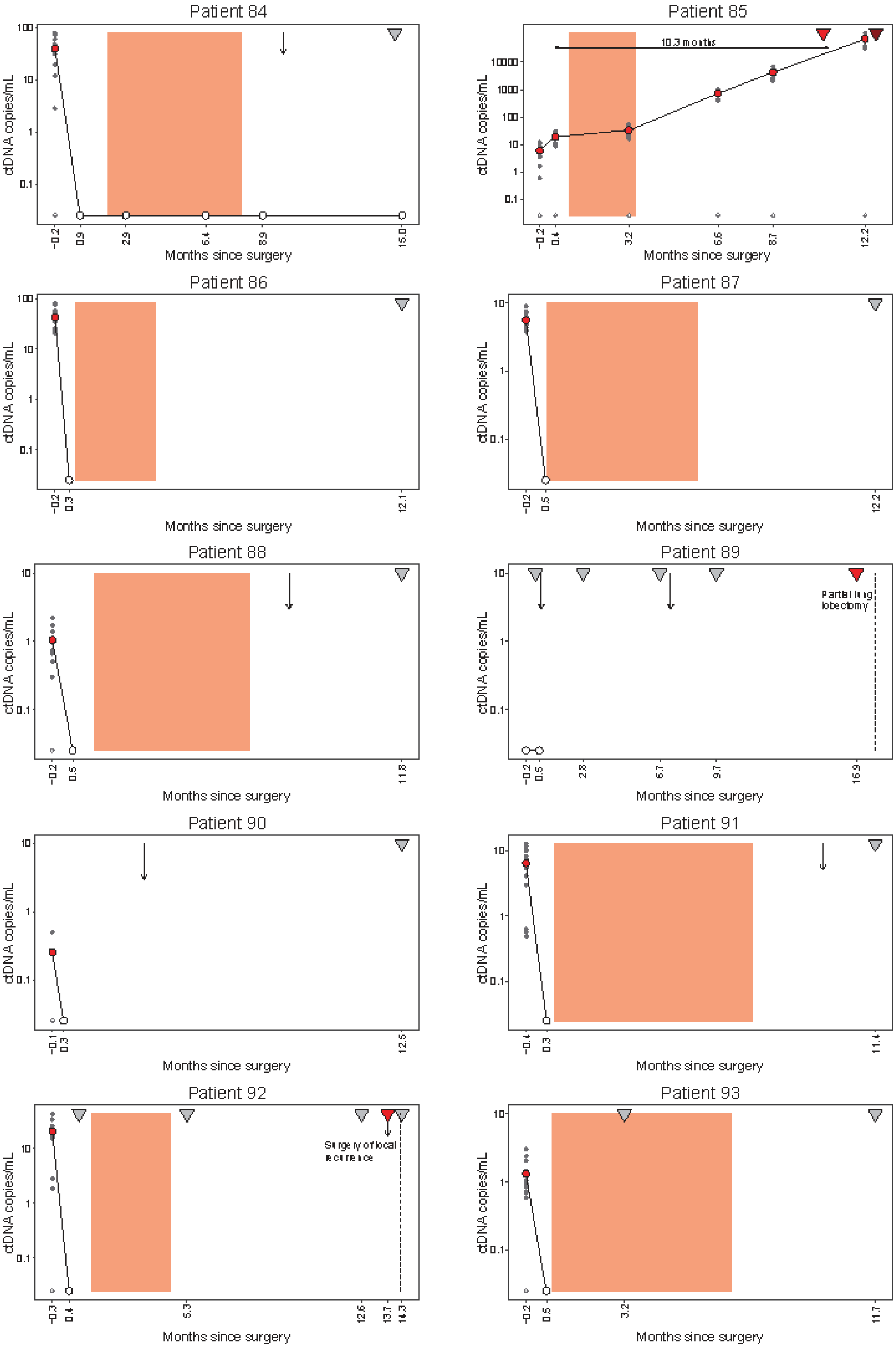


© 2019 Reinert T et al. *JAMA Oncology.*

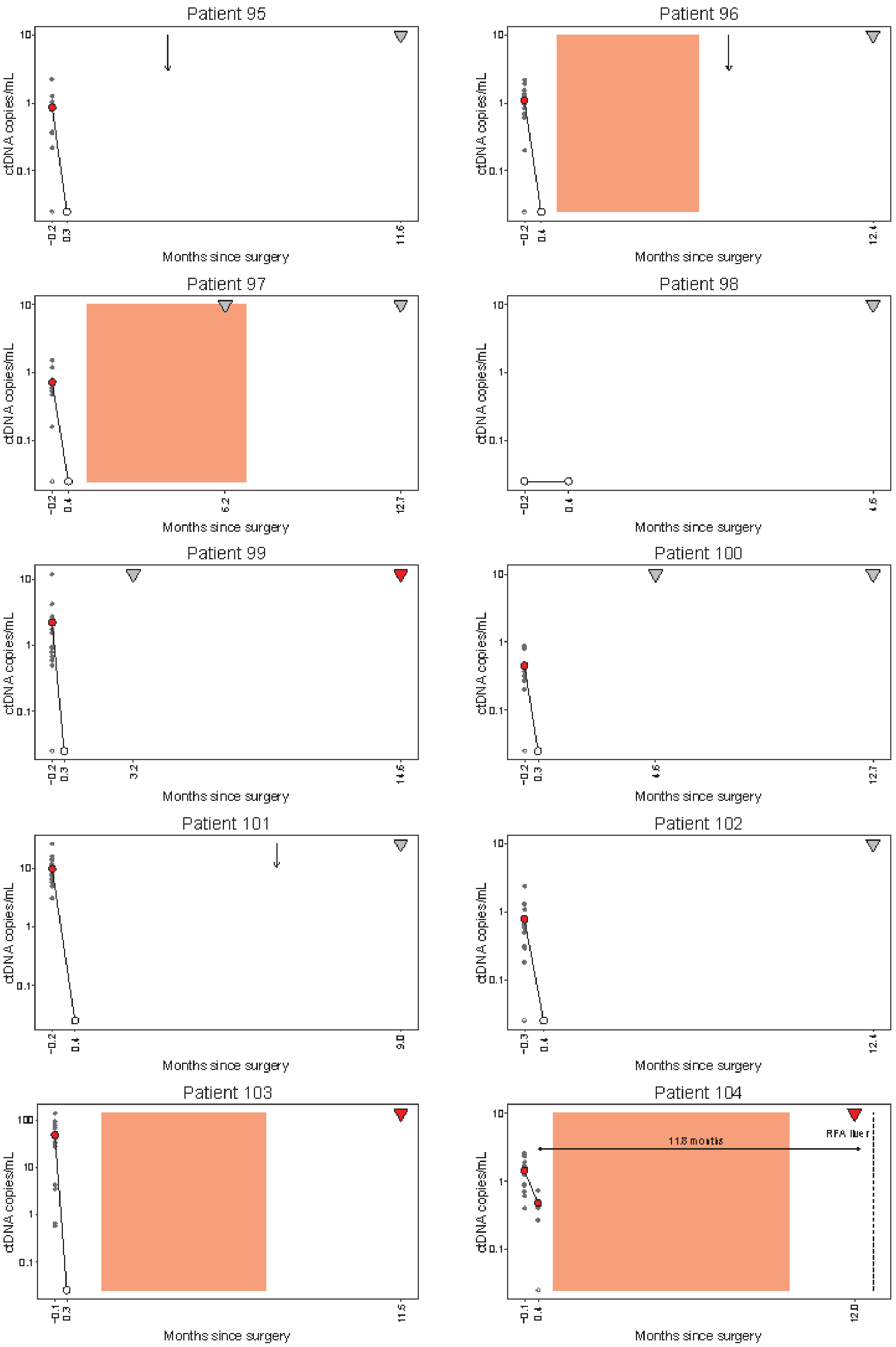


© 2019 Reinert T et al. *JAMA Oncology.*

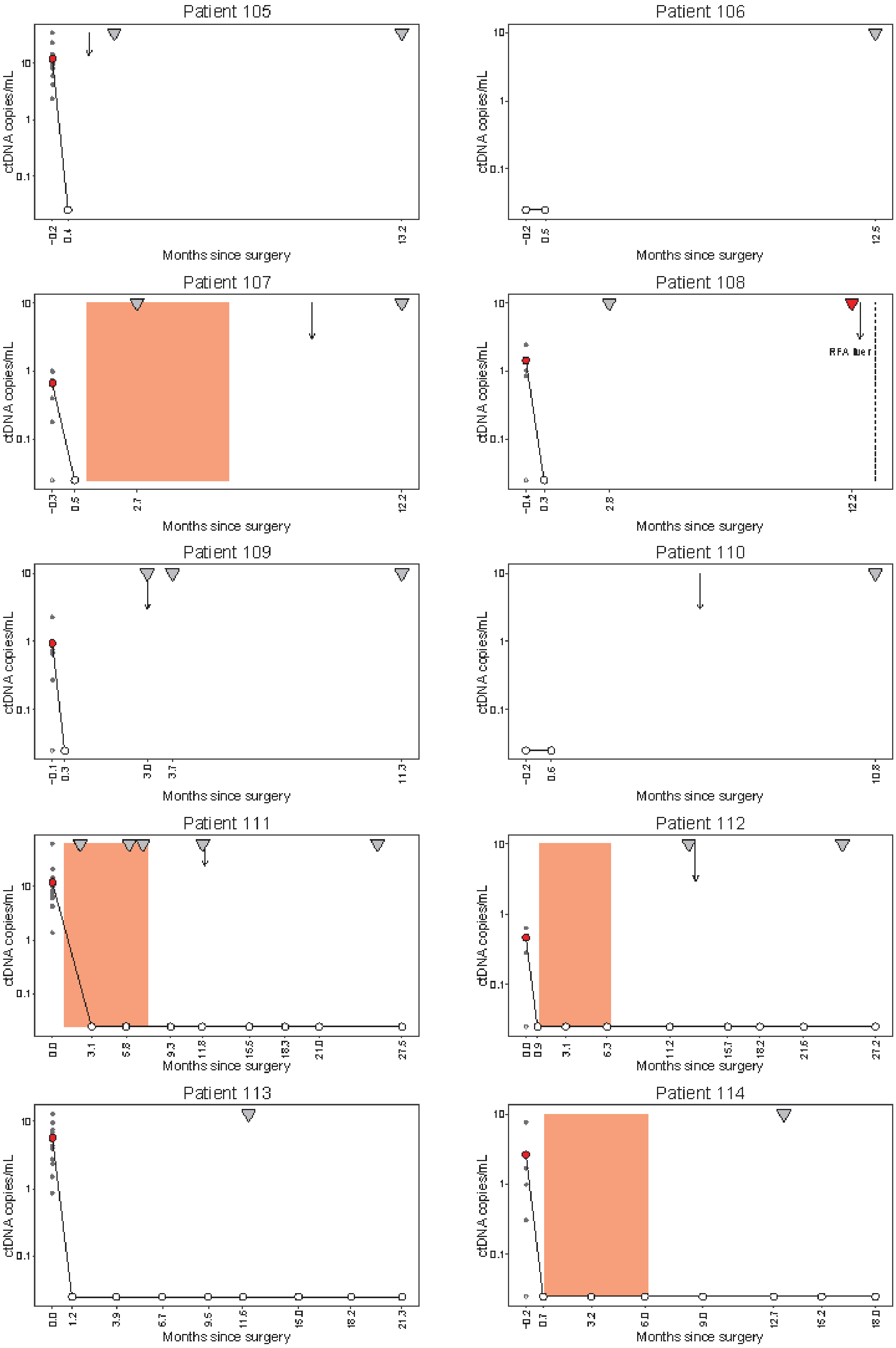


© 2019 Reinert T et al. *JAMA Oncology*.

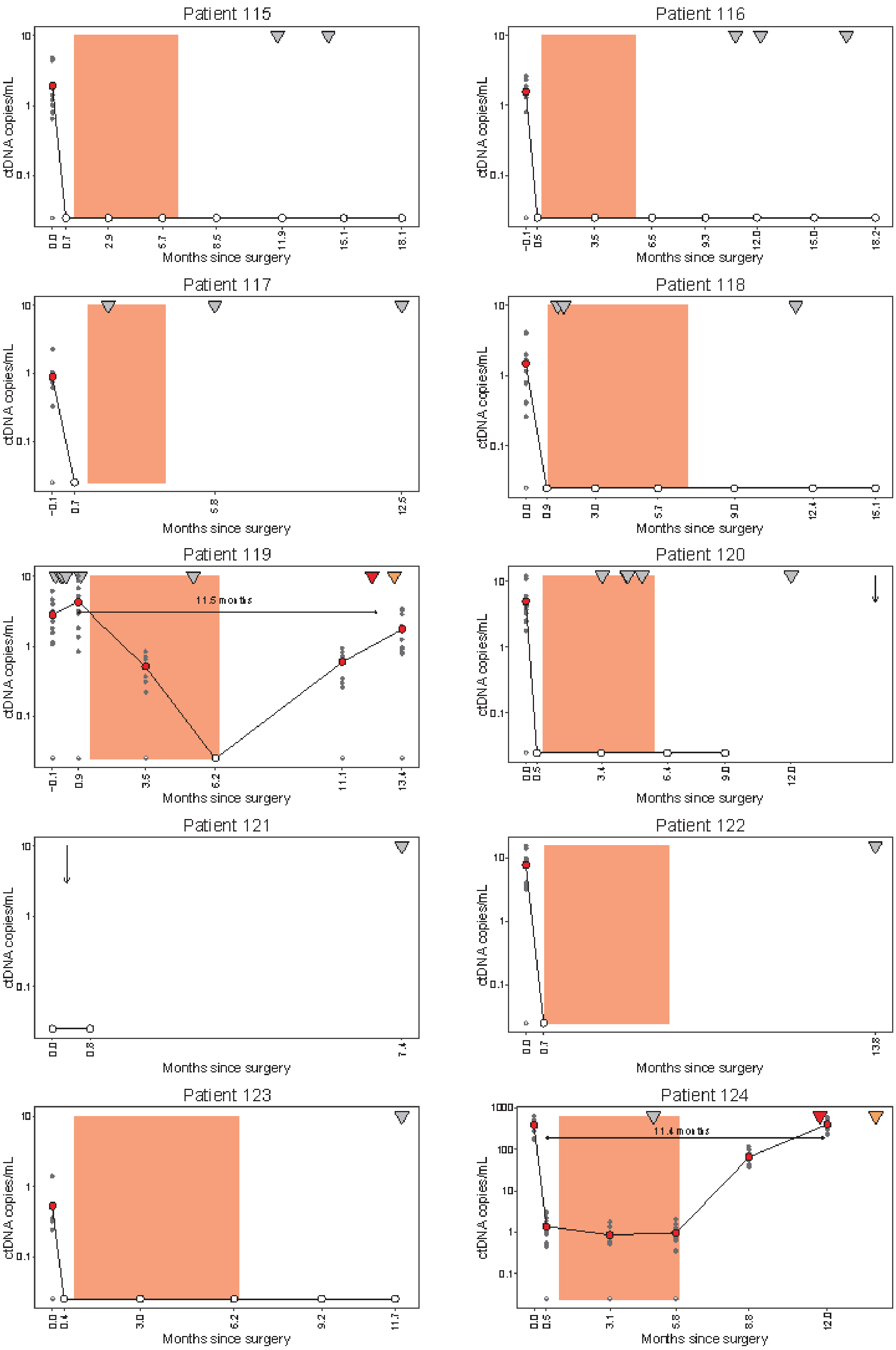


© 2019 Reinert T et al. *JAMA Oncology.*

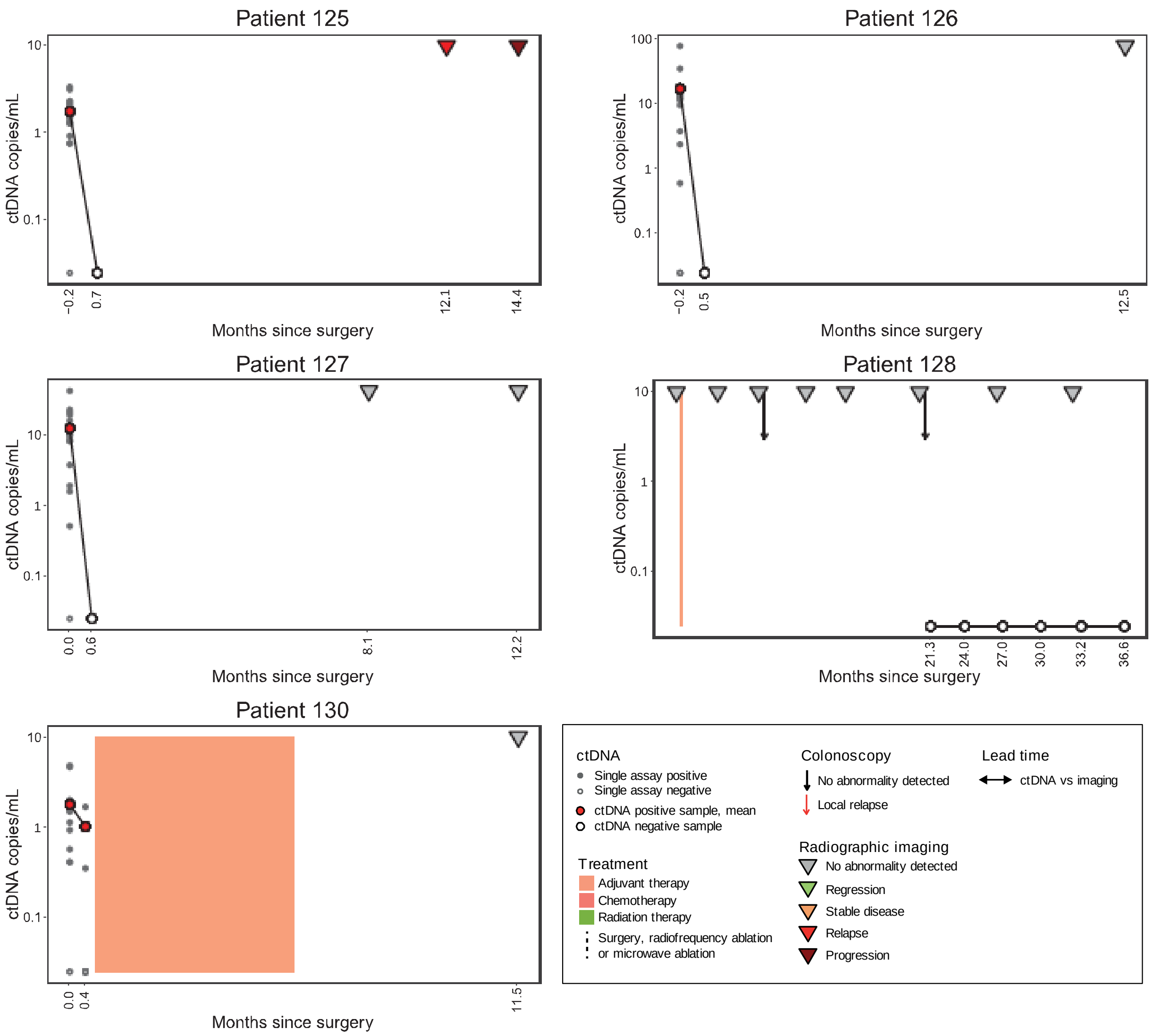


## eFigure 4. Pre-operative Detection of ctDNA and CEA in 122 Stage I-III CRC Patients.

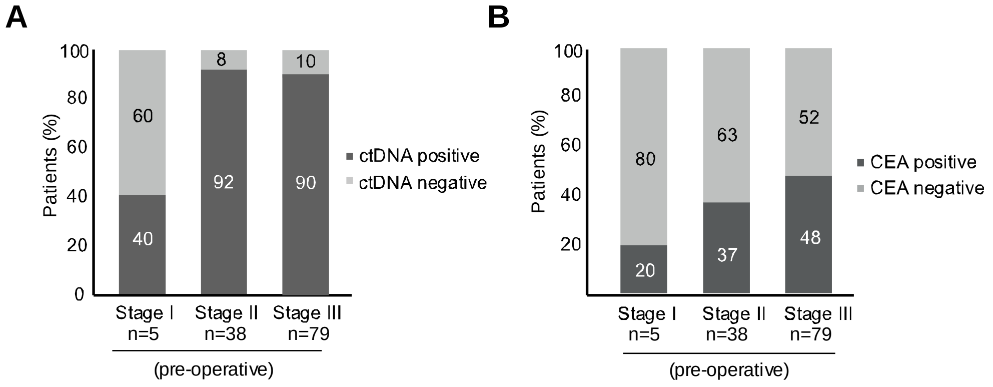


© 2019 Reinert T et al. *JAMA Oncology.*

**eFigure 5. ctDNA Profiling Results From the 94 Patients Included in the Day 30 ctDNA Analysis.** Patients are ordered by recurrence status and disease stage.

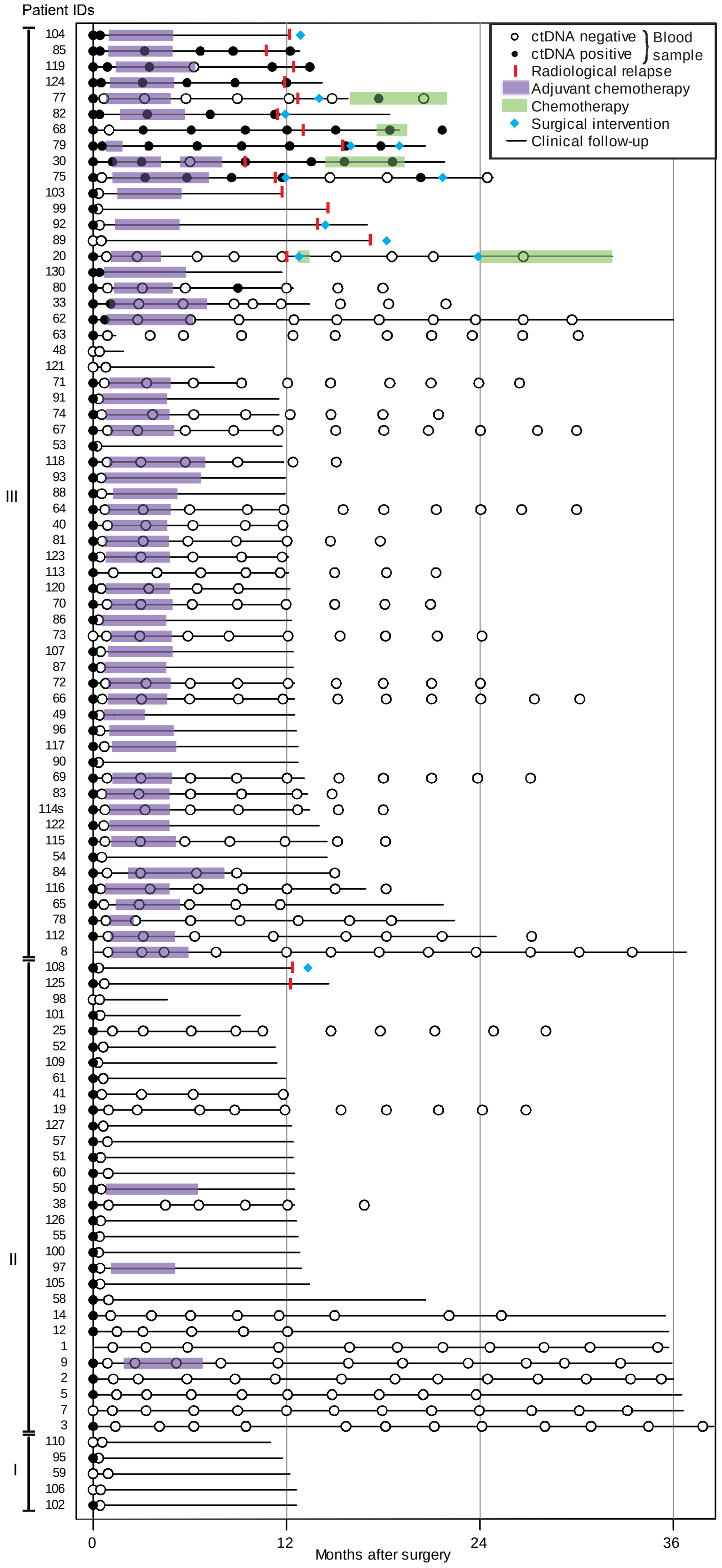


© 2019 Reinert T et al. *JAMA Oncology.*

**eFigure 6. ctDNA Profiling Results of the ACT Treated Fraction of Patients (n=52) Included in the Day 30 ctDNA Analysis.** A) Schematic overview of the ctDNA results. Patients are ordered by recurrence status and disease stage. B) Kaplan-Meier estimates (CI=95%) of recurrence free survival the 52 ACT treated patients, stratified by post-operative day 30 ctDNA status. The Kaplan Meier plot were halted when the proportion of patients in follow-up was under 10%.

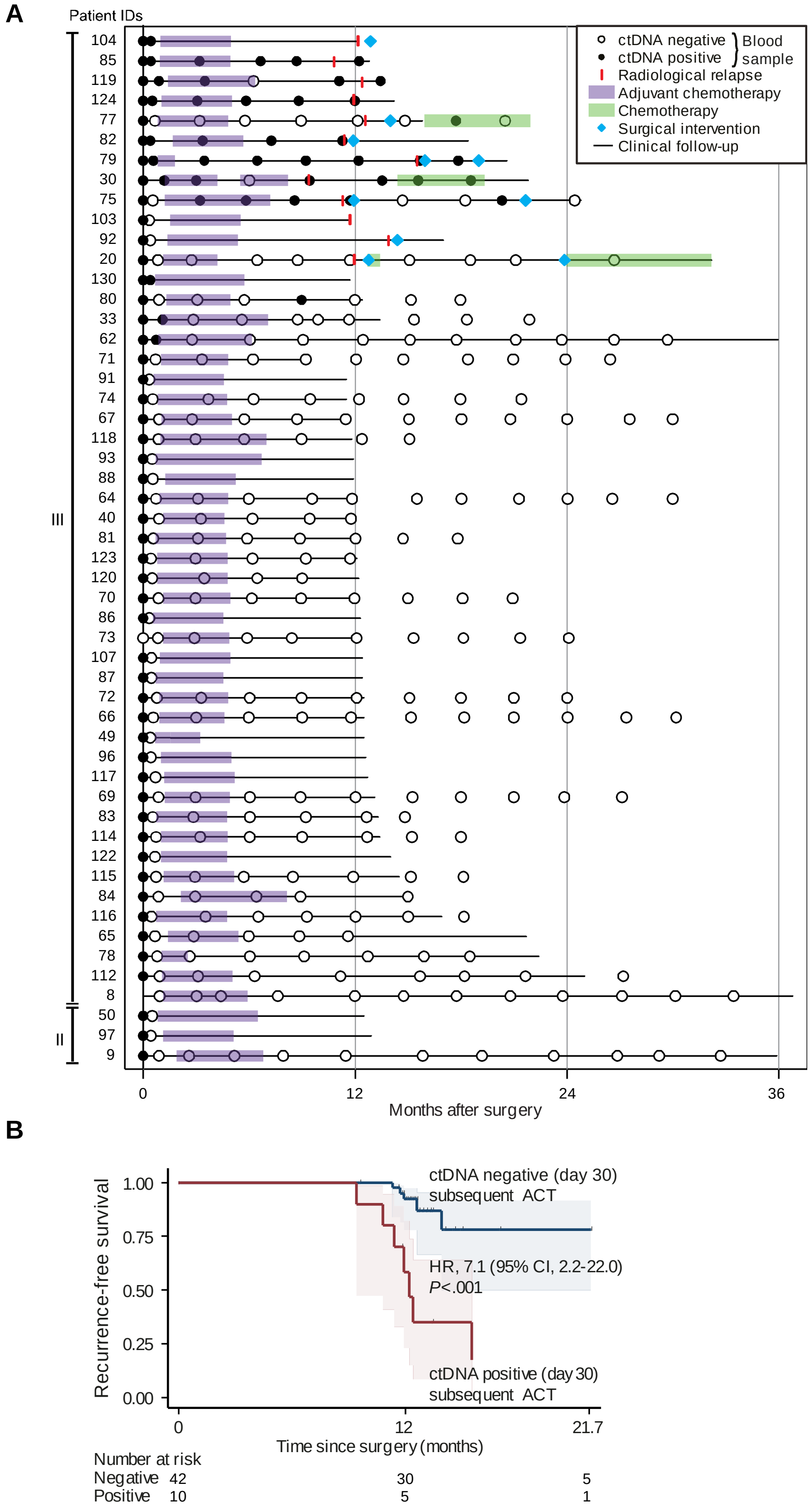


© 2019 Reinert T et al. *JAMA Oncology.*

**eFigure 7. ctDNA Profiling Results From the 75 Patients Included in the Longitudinal Post-Definitive-Treatment ctDNA Analysis.** Patients are ordered by recurrence status. Patients were considered positive if one or more plasma samples post-definitive-treatment was ctDNA-positive. Patient 80, which had a transient ctDNA-positive call at month 9 is likely false positive. From eFigure 3 it can be seen that only 2/16 mutations were called positive at month 9. All other post-operative samples from patient 80 were negative. In all other ctDNA-positive patients positivity persisted in the longitudinal samples and ctDNA status only changed to negative in case of clinical intervention.

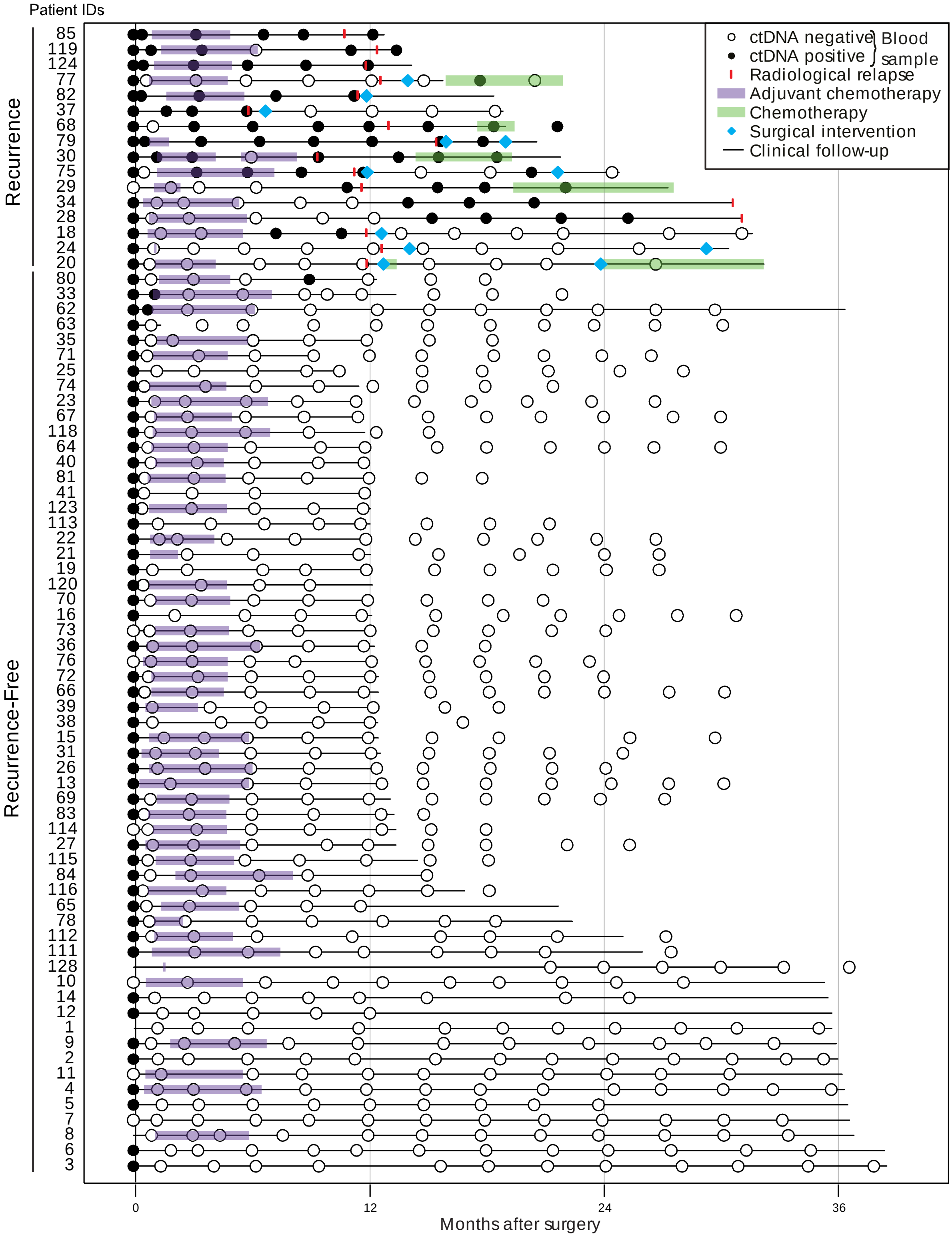


© 2019 Reinert T et al. *JAMA Oncology.*

**eFigure 8. CEA Profiling Results From the 75 Patients Included in the Post-Definitive Treatment ctDNA Surveillance Analysis.** Patients are ordered by recurrence status. Patients were considered positive if one or more surveillance samples were CEA positive.

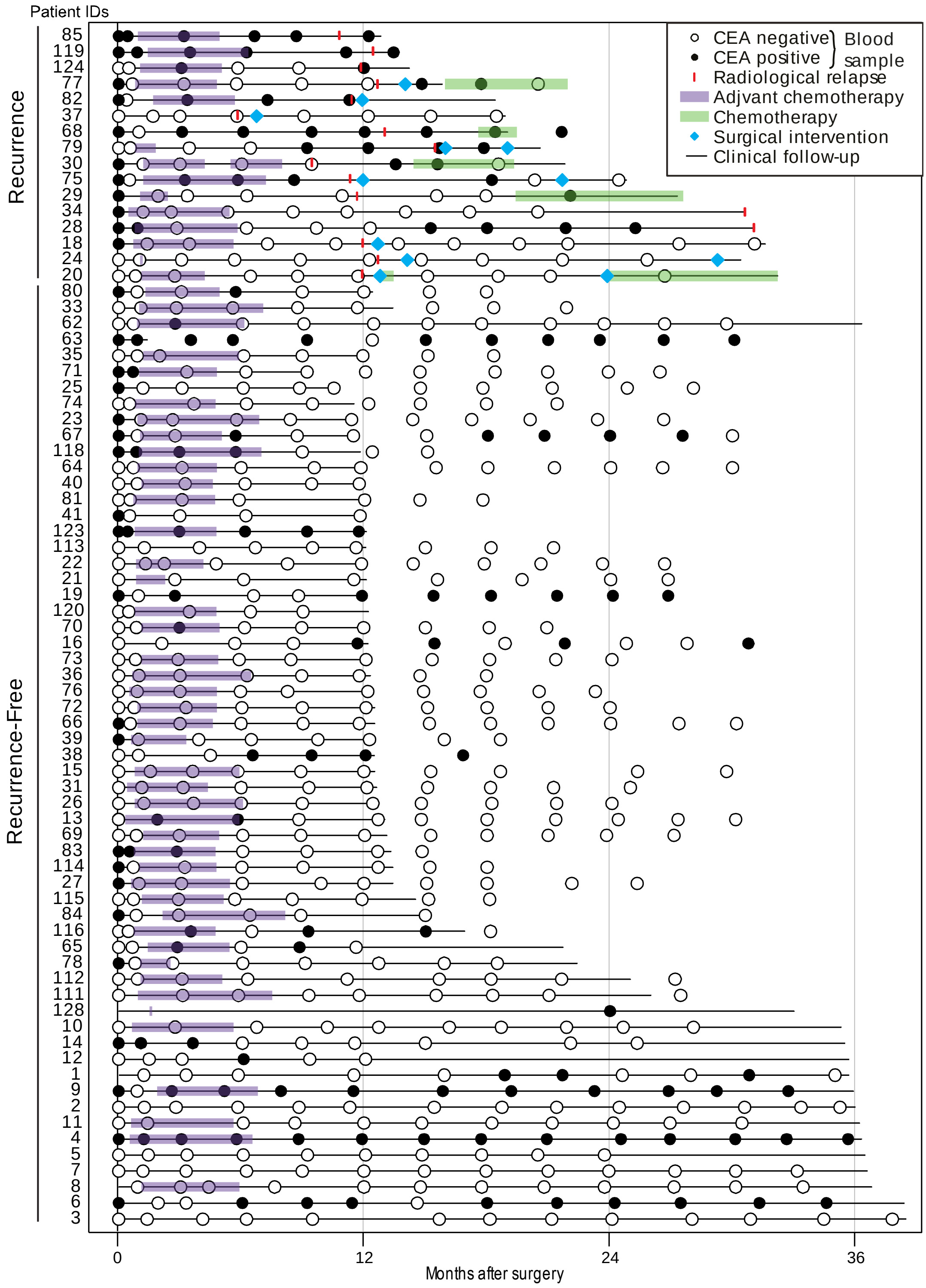


© 2019 Reinert T et al. *JAMA Oncology.*

## eFigure 9. Comparison of Time to Recurrence by CEA and Standard-of-Care CT Imaging.

The average time from surgery to relapse detection was 19 months for CEA and 13.6 months for CT imaging

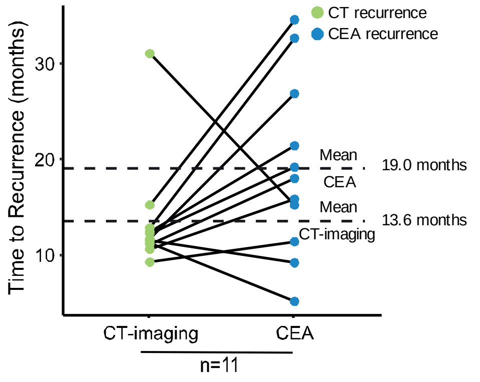


## eFigure 10. Clinical Trial Proposals for Investigating the Clinical Benefit of ctDNA-Guided Post-Operative Management of CRC Patients.

In the present study, we show that detection of circulating tumor DNA post-operatively, defines a patient subgroup with residual disease and very high risk of clinical recurrence, while ctDNA negative patients have low risk of recurrence. Based on these findings we suggest three clinical trials, aimed at investigating the clinical benefit of using ctDNA to guide the post-operative management. Trial 1: here we suggest investigating the effect of offering adjuvant chemotherapy to post-operative ctDNA-positive stage I and low risk stage II patients, who currently would not receive adjuvant chemotherapy. Trial 2: here we suggest investigating the effect of withholding adjuvant chemotherapy from ctDNA negative stage III patients, and hence likely cured patients, with the aim to spare them from unnecessary toxicity. Instead, the patients may be monitored by ctDNA based surveillance. Trial 3: here we suggest investigating the effect of differentiating the intensity of follow-up based on ctDNA-risk-stratification after adjuvant chemotherapy.

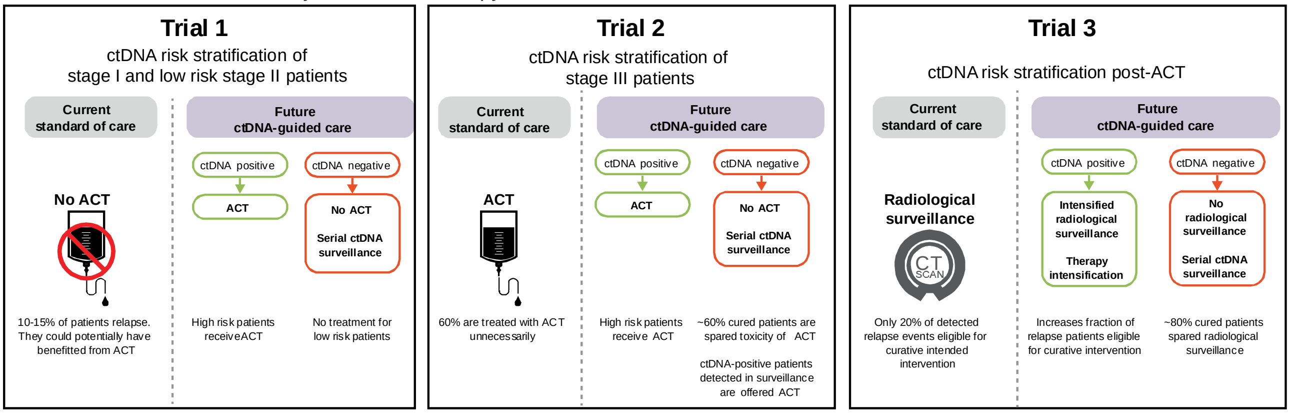


© 2019 Reinert T et al. *JAMA Oncology.*

## eReferences


1. Lamy P, Nordentoft I, Birkenkamp-Demtroder K, et al. Paired Exome Analysis Reveals Clonal Evolution and Potential Therapeutic Targets in Urothelial Carcinoma. *Cancer Res.* 2016;76(19):5894-5906.
2. Cheng DT, Mitchell TN, Zehir A, et al. Memorial Sloan Kettering-Integrated Mutation Profiling of Actionable Cancer Targets (MSK-IMPACT): A Hybridization Capture-Based Next-Generation Sequencing Clinical Assay for Solid Tumor Molecular Oncology. *J Mol Diagn.* 2015;17(3):251-264.
3. MuTect2 Pitfalls — Best Practices for Processing HTS Data 0.0 documentation. https://best-practices-for-processing-hts-data.readthedocs.io/en/latest/mutect2_pitfalls.html. Accessed October 26, 2018.
4. Obenchain V, Lawrence M, Carey V, Gogarten S, Shannon P, Morgan M. VariantAnnotation: a Bioconductor package for exploration and annotation of genetic variants. *Bioinformatics*. 2014;30(14):2076-2078.
5. Gehring JS, Fischer B, Lawrence M, Huber W. SomaticSignatures: inferring mutational signatures from single-nucleotide variants. *Bioinformatics*. 2015;31(22):3673-3675.
6. https://cancer.sanger.ac.uk/cosmic/signatures.
7. Blokzijl F, Janssen R, van Boxtel R, Cuppen E. MutationalPatterns: comprehensive genome-wide analysis of mutational processes. *Genome Med*. 2018;10(1):33.
8. Alexandrov LB, Nik-Zainal S, Wedge DC, et al. Signatures of mutational processes in human cancer. *Nature*. 2013;500(7463):415-421.
9. Abbosh C, Birkbak NJ, Wilson GA, et al. Phylogenetic ctDNA analysis depicts early-stage lung cancer evolution. *Nature*. 2017;545(7655):446-451.
10. McGranahan N, Favero F, de Bruin EC, Birkbak NJ, Szallasi Z, Swanton C. Clonal status of actionable driver events and the timing of mutational processes in cancer evolution. *Sci Transl Med*. 2015;7(283):283ra54.
11. Zhang J, Kobert K, Flouri T, Stamatakis A. PEAR: a fast and accurate Illumina Paired-End reAd mergeR. *Bioinformatics*. 2014;30(5):614-620.
12. Coombes C, Page K, Salari R, et al. Personalized detection of circulating tumor DNA antedates breast cancer metastatic recurrence [published online April 16, 2019]. *Clin Cancer Res*. doi:10.1158/1078-0432.CCR-18-3663.
13. Lash TL, Riis AH, Ostenfeld EB, et al. Associations of Statin Use With Colorectal Cancer Recurrence and Mortality in a Danish Cohort. *Am J Epidemiol*. 2017;186(6):679-687.


© 2019 Reinert T et al. *JAMA Oncology.*
